# Absent the pre-B cell receptor checkpoint, the B-1a immunoglobulin CDR-H3 repertoire normalizes by convergent selection

**DOI:** 10.3389/fimmu.2026.1733041

**Published:** 2026-03-16

**Authors:** Lucas Tostes Costa Vaz, Tessa Blackburn, Ada Elgavish, Andre Macedo Vale, Peter D. Burrows, Harry W. Schroeder, Mohamed Khass

**Affiliations:** 1 Department of Medicine, University of Alabama at Birmingham, Birmingham, AL, United States; 2 Program in Immunobiology, Carlos Chagas Filho Biophysics Institute, Federal University of Rio de Janeiro, Rio de Janeiro, Brazil; 3 Department of Microbiology, University of Alabama at Birmingham, Birmingham, AL, United States; 4 Department of Endodontics, University of Alabama at Birmingham, Birmingham, AL, United States

**Keywords:** antibody repertoire, B cell development, B-1 cells, immunoglobulin, Lambda 5, peritoneal cavity, pre B cell receptor, surrogate light chain

## Abstract

Peritoneal cavity (PerC) B cells arise via a specific set of ontogenetic and antigen-driven selection pressures. A large portion of the PerC B-1 population, including B-1a and B-1b cells, is fetal-derived and thus, due to a lack of N nucleotide addition, produces an immunoglobulin repertoire that is characteristically germline-encoded. While conventional B-2 cells depend on efficient pairing of surrogate light chain (SLC) and μ Heavy Chain (HC) to pass through the preBCR checkpoint, the extent to which the passage of the B-1 population through the preBCR checkpoint affects the composition of its repertoire has remained an open question. In this work, we used mice deficient in λ5, a key component of the SLC, to test whether the absence of preBCR selection would yield natural, germline encoded, PerC B-1 CDR-H3 repertoires similar to WT. And, if not, whether a more WT-like repertoire at the protein level would be recreated by convergent cellular evolution. We found that the absence of preBCR selection led to categorical differences in DH-specific gene segment usage, RF preference, and N addition in PerC B-1a, B-1b, and B-2 CDR-H3s when compared to WT. Despite this marked divergence in nucleotide sequence, at the translated amino acid level, the B-1a CDR-H3 repertoire generated in the absence of preBCR selection exhibited partial convergence to the WT sequence, most notably with selection for tyrosine; this phenomenon was less apparent in B-1b and B-2 cells. This convergence was not evident at the nucleotide level and did not restore the underlying VDJ architecture. These results suggest that while the initial CDR-H3 repertoire is altered genetically in the absence of preBCR selection, natural self-antigen selection and self-renewal in the B-1a compartment promote the creation of a CDR-H3 repertoire that has considerable overlap in amino acid sequence with its wild-type counterpart.

## Introduction

Immunoglobulin (Ig) genes are created by a complex series of gene segment rearrangement and modification events (1, 2), which include somatic addition, subtraction, and/or mutation of the germline sequence (2–4). Early in life, in the fetal liver (FL) and then postnatally in the bone marrow (BM), developing B cell progenitors rearrange their Variable (V_H_), Diversity (D_H_), and Joining (J_H_) gene segments, resulting in the production of an Ig μ Heavy Chain (HC). This combinatorial V_H_-D_H_-J_H_ gene segment rearrangement creates Complementarity Determining Region-3 of the heavy chain (CDR-H3), which contains the carboxy terminus of the V_H_, the D_H_, and the amino terminus of the J_H_ gene segments. In the 3D structure of Ig, CDR-H3 lies at the center of the classic antigen-binding site. This central location allows CDR-H3 to often play a critical role in the recognition and immune response to antigens (1, 2).

In the H chain, in addition to the combinatorial diversity permitted by access to arrays of V_H_, D_H,_ and J_H_ gene segments, the D→J and V→D junctions are somatically diversified by the variable loss or palindromic (P) gain of terminal gene segment nucleotides (3), and by the random addition of N nucleotides (4, 5). These mechanisms make CDR-H3 not only the focus for antigen binding, but also the focus for the initial diversification of the antibody repertoire.

**Figure 1:**
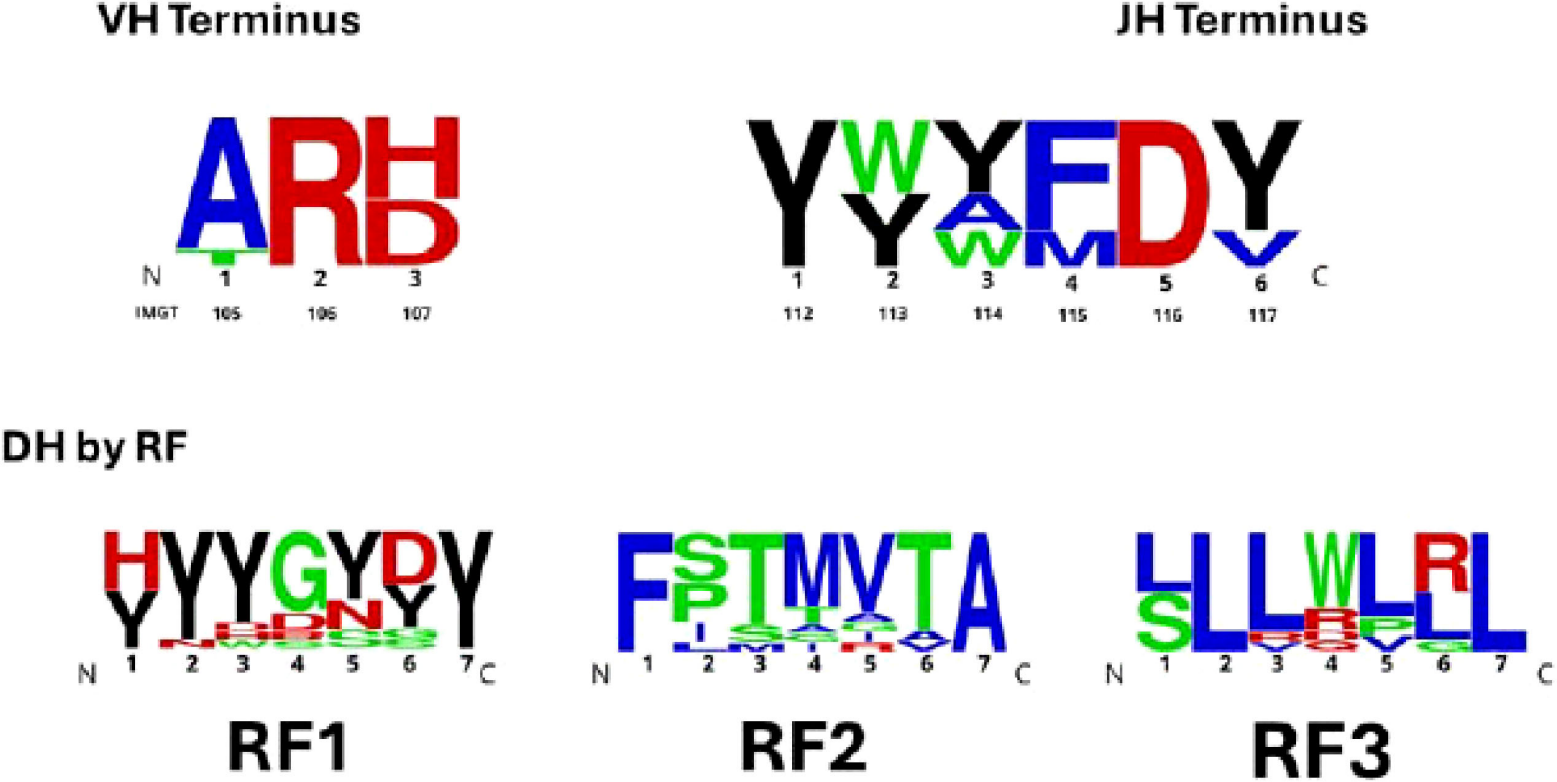
V_H_, D_H_, and J_H_ germline sequences that contribute to CDR-H3 amino acid content. Upper panel, the proximal two V_H_ amino acids (AR) from the amino (N) terminus of the CDR-H3 and the distal three J_H_ amino acids (FDY) from the carboxy (C) terminus comprise the base of the CDR-H3. The amino acids downstream of V_H_ sequences and upstream of the J_H_ sequences that contribute to the CDR-H3 base compose the CDR-H3 loop. Lower panel, amino acids encoded by each D_H_ reading frame among all thirteen (BALB/c) DH gene segments. Reading frame amino acid signatures of each of the four D_H_ families and the individual sequences of each J_H_ gene segment are shown in **Supplementary Figure 1**. With the exception of tyrosine, which is in black for emphasis, amino acids were colored according to their relative hydropathicity which ranges from red (charged) arginine to green (neutral) glycine to blue (hydrophobic) isoleucine.

Although at first glance these complex mechanisms appear to promote unrestricted, random CDR-H3 diversity, distinctive categorical preferences for CDR-H3 content in specific B cell subsets can be easily detected through comparisons of even small numbers of sequences (*e.g.*, 50 or fewer). Preferences may include biases in V_H_, D_H_, or J_H_ gene segment usage; in D_H_ reading frame (RF) usage; and in the number (*i.e.*, length) and physicochemical properties (hydrophobicity, hydrophilicity, or charge) of the encoded amino acids (5–7). Some of these categorical constraints are ontogenetically predetermined (8–12). For example, each D family of sequences exhibits its own characteristic set of evolutionarily favored amino acid signatures by RF (**Figure 1**, **Supplementary Figure 1**). Because preferential rearrangement at sites of microhomology between D_H_ and J_H_ sequences favors the use of RF1, enrichment for tyrosine (Y) often follows (**Figure 1**, **Supplementary Figure 1**).

Additional constraints based on antigen reactivity are progressively imposed by somatic mechanisms as developing B cells pass through critical selective developmental checkpoints in primary or secondary lymphoid tissues (5, 13–15). For example, Ig development begins in preB cells with the creation of a µ Heavy Chain (µHC). Together, two non-covalently bound proteins, λ5 and VpreB, form the surrogate light chain (SLC). The binding of the µHC to the SLC forms the preB cell receptor (preBCR), whose signals promote proliferation and transition into the late preB cell stage, where conventional LC V_L_ and J_L_ gene segments are rearranged. Testing of the µHC by SLC is the first checkpoint for the functionality of a nascent Ig (16). Binding is mediated through interaction between CDR-H3 and a sensing site on VpreB (17) that favors hydrophilic amino acids, most markedly tyrosine at position 101 (18) [IMGT position 109 (19, 20)]. Late preB cells from mice lacking λ5 (λ5KO) have increased use of D_H_ RF2, which lacks tyrosine. Increased use of this RF shifts CDR-H3 into a more hydrophobic range (18). Enrichment for tyrosine still occurs, but later, in the periphery, as immature B cells transition into mature, recirculating B cells (21).

Three key B cell subsets that are found particularly in the peritoneal cavity (PerC) variably demonstrate specific categorical effects of ontogenetic and antigen-driven selection. These subsets are designated B-1a, B-1b, and B-2. B-1a cells are identified by the surface expression of CD5 (22, 23). Many B-1a cell clones originate in the fetal liver. VDJ rearrangement in the fetus is marked by a paucity of N nucleotide addition, and thus enrichment for germline sequence. As a result, fetal B-1a CDR-H3 content is ontogenetically programmed to encode a high frequency of tyrosine residues (24). Other B-1a cells develop postnatally in the bone marrow (25, 26). B-1a cells characteristically produce polyreactive, low-affinity natural antibodies that bind apoptotic debris from dying cells as well as other self-antigens (27–29). These antibodies often cross-react with antigens found on microbial pathogens and are the mainstay of B cell innate immunity to many infectious agents (30–32). B-1a self-renewal capacity enables long-term maintenance of fetal-derived antigen specificities (33–35).

B-1b and B-2 cells mostly derive from postnatal progenitors in the bone marrow (25, 36–40) and, unlike B-1a cells, B-1b cells do not express cell surface CD5. B-1b-derived antibodies also bind self-antigens and provide durable, long-term protection against many microbes (41). PerC B-2 cells in this compartment are considered to be part of the conventional mature B cell repertoire. Compared to their mature B cell counterparts in blood, spleen, and bone marrow, however, PerC B2 cells exhibit fewer N additions and more hydrophobic-enriched CDR-H3 transcripts (24, 41). A common antigen receptor-mediated selection process has been proposed to shape the repertoires of all three PerC B cell subsets (24).

Given the heavily germline-encoded composition of Ig produced by fetal-derived B cells, the role of preBCR selection in shaping the B-1 repertoire has been questioned (42–46). In this work, we sought to test whether a WT-like natural B1 CDR-H3 repertoire would be preserved in the absence of preBCR selection or whether a WT repertoire would be recreated in real time by convergent selection (*i.e.*, a different nucleotide sequence starting point, but a similar protein sequence outcome). We obtained µHC nucleotide sequences from the three PerC B cell subsets from homozygous λ5-deficient (λ5KO) mice and compared their unique CDR-H3s to previously reported CDR-H3 nucleotide sequences from WT PerC B cells and from WT bone marrow immature B cells (24). Categorical differences in D_H_-specific gene segment usage, RF preference, and N addition were observed between WT and λ5KO CDR-H3s, consistent with the absence of SLC-mediated selection. Notably, however, when translated to the amino acid level, the λ5KO B-1a CDR-H3 repertoire demonstrated convergence toward a more WT-like B-1a profile, with marked enrichment for tyrosine residues. This pattern was less apparent in B-1b and B-2 cells. These results suggest that while the initial CDR-H3 repertoire is altered in the absence of preBCR selection, natural self-antigen selection and self-renewal in the PerC B-1a compartment promote the creation of a CDR-H3 repertoire that has considerable overlap in amino acid sequence with its wild-type counterpart. While not definitive, these findings are suggestive of idiotope-specific somatic selection of the protein sequence of CDR-H3 in B-1a B cells.

## Materials and methods

### Study design

In this work, we compared CDR-H3 sequences of newly obtained (PerC) B-1a, B-1b, and B-2 cells from 8-12-week-old λ5-deficient (λ5KO) mice to previously generated wild-type (WT) and λ5KO-deficient bone marrow immature B cells (fraction E) and WT peritoneal cavity (PerC) B-1a, B-1b, and B-2 cells from BALB/c mice (21, 24). We chose to include immature B cells (fraction E) in the comparison since this subset is the final subset developed in the bone marrow that later gives rise to mature B cells in the periphery. Fraction E cells were identified based on the expression of CD19, B220, AA4.1, CD43, IgM, and the absence of IgD expression, as described previously (21, 24). We defined the CDR-H3 region as comprising amino acids from positions 105 to 117, inclusive, per IMGT designation (20). Our sequence-level analysis used only unique non-repeated CDR-H3s to control for pseudo replication. We focused on CDR-H3 since this region is the major factor in B cell development and binding/neutralization of antigens. We chose to use Sanger sequencing of cloned VH7183DJCµ transcripts because we have previously generated a robust database of similar sequences from WT and mutant D_H_ mice to serve as comparisons. We used six age-matched female littermate λ5KO mice to obtain CDR-H3 sequences after sorting and then performed RT-PCR on the VH7183-DJ Cµ transcripts of peritoneal cavity subsets. In total, there were 81 fraction E, 116 B-1a, 107 B-1b, and 103 B-2 sequences from λ5KO mice, compared to a total of 255 fraction E, 151 B-1a, 118 B-1b, and 114 B-2WT mice, for which previously published CDR-H3 sequences were available (21, 24). All H chain sequences were unique. A list of these unpublished PerC B-1a, B-1b, and B-2 λ5KO sequences is provided in **Supplementary Table 1**.

Additional sets of 8-12-week-old WT and λ5KO mice were used for quantification of absolute B cell numbers and evaluation of the frequency of BrdU incorporation and apoptosis.

### Mice

All experiments and animal procedures were performed using protocols approved by the University of Alabama Institutional Animal Care and Use Committee (IACUC). BALB/c mice were bred in the UAB vivarium under specific pathogen-free conditions. λ5KO mice were originally on a C57BL/6 background (47) and had been previously backcrossed for 22 generations onto a BALB/c background. All mice were housed in the same animal facility location at UAB to control for environmental variations. In order to take advantage of our previously published CDR-H3 sequence and B cell development datasets from WT and λ5KO mice, we focused our analysis on female age-matched littermates. Animal euthanasia was performed using CO2 inhalation with a gradual displacement rate from 30% to 70% of the chamber volume per min, followed by cervical dislocation according to UAB guidelines based on federal regulations (48–50).

### Flow cytometry and cell sorting

The peritoneal cavity lavage of 8-12-week-old mice was obtained, and single-cell suspensions were prepared as previously described (7, 13, 24, 51). A BD FACS Aria II instrument (BD Biosciences, Becton, NJ) was used for sorting. Cells were washed, stained, and analyzed using the following labeled antibodies: anti-CD19 (APC-Cy7), anti-CD5 (PE) [BD Pharmingen, San Diego, CA], and anti-Mac-1 (PB) [BioLegend, San Diego, CA]. Propidium Iodide (PI) was used to exclude dead cells.

### RNA preparation, RT-PCR, and sequencing

Total RNA isolation, VH7183-specific VDJCμ RT-PCR amplification, cloning, sequencing, and analysis were performed as previously described (13, 24, 51). Sequences for each B cell subset were obtained from at least six age-matched littermate mice, and the analysis used only unique non-repeated CDR-H3 sequences to control for pseudo replication.

### Evaluation of the extent of apoptosis

The FITC Annexin V Apoptosis Detection Kit II [BD Biosciences, San Jose, CA], with the aid of propidium iodide, was used to identify Annexin V-positive cells.

### Bromodeoxyuridine labeling

The frequency of Bromodeoxyuridine (BrdU) incorporation in peritoneal cavity subsets was determined after adding BrdU (2%) to the drinking water for one week. Mice were sacrificed, peritoneal cavity lavage was collected, cells were washed and fixed, and BrdU-positive B cells were detected using a BrdU-specific antibody and the BD BrdU-flow kit [BD Biosciences] according to the manufacturer’s protocol.

### Deconstruction of CDR-H3

The BALB/c IgH locus contains 18 VH7183 family members, 13 D_H_, and 4 functional J_H_ gene segments. Among the 13 D_H_ gene segments, two belong to the DFL family (IMGT D1), nine to the DSP family (IMGT D2), one to the DST family (IMGT D3), and one to the DQ52 family (IMGT D4) (19). Using bioinformatics, our comprehensive analysis included marking the presence or absence of an identifiable D_H_ gene segment, the extent of 3’ V_H_ loss, the extent of palindromic 3’ V_H_ P junction addition, the extent of overlap between V and D, the extent of N addition between V and D, the inclusion of 5’ palindromic D_H_ P junction sequence, the loss of 5’ D_H_ sequence, the total number of D_H_ nucleotides included in the CDR-H3 sequence, the loss of 3’ D_H_ sequence, the inclusion of 3’ palindromic D_H_ (P) junction sequence, the extent of overlap between D and J, the extent of N addition between D and J, the length of non-V and/or -J sequence in sequences lacking identifiable D_H_ sequence (NDN), the inclusion of 5’ palindromic J_H_ P junction sequence, the loss of 5’ J_H_ sequence, the total loss of germline sequence, the total amount of identifiable germline sequence, the total amount of N addition, the total length of CDR-H3 in codons, and the average hydrophobicity of the CDR-H3 loop as assessed by the Kyte Doolittle hydrophobicity index as modified by Eisenbarth (52) (**Supplementary Table 2**).

### Sequence depictions

CDR-H3 sequences were visualized on a position-by-position basis using WebLogo (https://weblogo.threeplusone.com/). With the exception of tyrosine, which is in black for emphasis, amino acids were colored according to their relative hydropathicity which ranges from red (charged) arginine to green (neutral) glycine to blue (hydrophobic) isoleucine. The data used to generate the sequence logos are shown in Detailed sequence analysis file (**Supplementary Table 1**).

### Statistical analysis

The differences between peritoneal cavity B cell subsets were evaluated, when appropriate, using the two-tailed Fisher’s exact test, two-tailed Student’s t-test, or Levene’s test for the homogeneity of variance. Analysis was performed with JMP Pro version 17 (SAS Institute, Inc., Cary, NC).

## Results

### Comparison of peritoneal cavity B cell numbers in λ5KO mice and WT littermates

**Figure 2:**
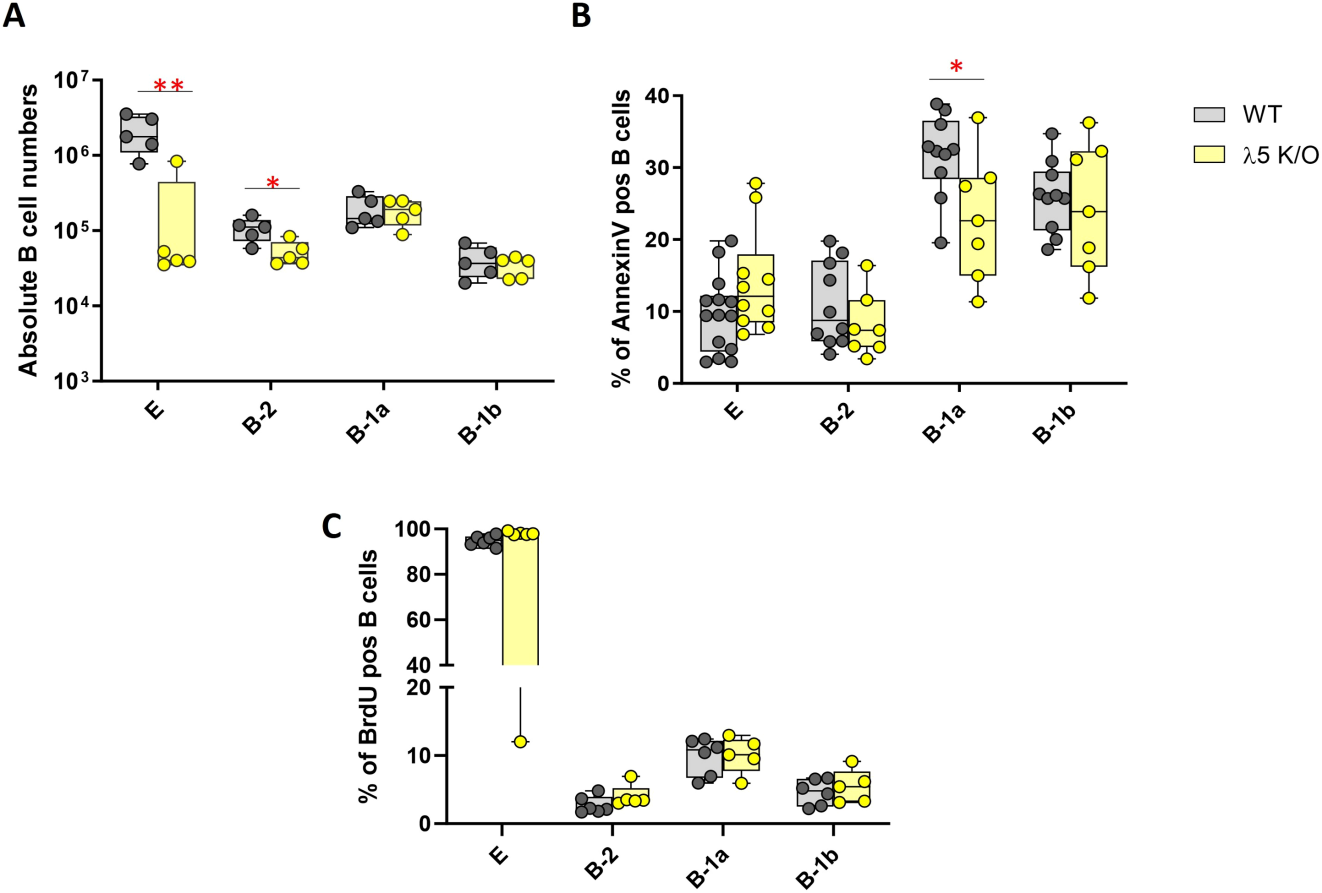
Comparison of B cell numbers and cell turnover in WT and λ5KO B cells from bone marrow fraction E and from peritoneal cavity B-1a, B-1b, and B-2 B cells. **(A)** Absolute B cell numbers. **(B)** Percent of Annexin V-positive B cells. **(C)** Percent of B cells incorporating BrdU after one week’s exposure to BrdU in the drinking water. Statistical significance is indicated by asterisks, *p<0.05 and **p<0.01.

We previously reported that the absolute number of bone marrow fraction E cells from 8-12-week-old λ5KO BALB/c mice was approximately one-tenth that of WT controls (p = 0.0076) (21) (**Figure 2A**, **Table 1**). In this work, we observed complete reconstitution of the absolute numbers of B-1a and B-1b cells of PerC from 8-12-week-old female λ5KO BALB/c mice, reaching levels comparable to WT. In contrast, the absolute numbers of B-2 cells in λ5KO mice were only half that of WT (p = 0.019*)*.

**Table 1:** Absolute B cell numbers per cubic millimeter and the percent of B cells undergoing apoptosis or cell turnover.

B subpopulation	Tissue	Assay	WT	λ5KO	p-value
Fxn. E	Bone Marrow	Absolute B cell Number (10^5^)	21.1 ± 5.1	2.0 ± 3.8	*0.0076*
		Annexin V (%)	9.6 ± 1.4	14.1 ± 2.3	
		BrdU (%)	94.8 ± 0.5	98.1 ± 0.2	
B-2	Peritoneal Cavity	Absolute B cell Number (10^5^)	10.7 ± 1.6	5.2 ± 0.9	*0.019*
		Annexin V (%)	10.9 ± 1.8	8.1 ± 1.7	
		BrdU (%)	2.7 ± 0.5	4.0 ± 0.6	
B-1a	Peritoneal Cavity	Absolute B cell Number (10^5^)	19.3 ± 4.1	18.4 ± 3.1	
		Annexin V (%)	31.7 ± 1.8	23.0 ± 3.3	*0.026*
		BrdU (%)	9.8 ± 1.1	10.0 ± 1.2	
B-1b	Peritoneal Cavity	Absolute B cell Number (10^5^)	4.1 ± 0.9	3.4 ± 0.5	
		Annexin V (%)	25.9 ± 1.6	24.4 ± 3.5	
		BrdU (%)	4.6 ± 1.9	5.4 ± 2.5	

Only statistically significant *p* values (≤ 0.05) are shown (red font).

### The frequency of apoptotic cells in the λ5-deficient peritoneal cavity population

Using Annexin V positivity as a measure of apoptosis, we observed that bone marrow fraction E cells from 8-12-week-old λ5KO mice were ~50% more likely to be undergoing apoptosis than WT controls. However, this difference did not achieve statistical significance (p=0.094) (**Figure 2B**, **Table 1**). In the PerC, by contrast, λ5KO B-2 cells were ~20% less likely to be undergoing apoptosis than WT, although again, this difference did not achieve statistical significance. Conversely, λ5KO B-1a cells were ~50% less likely to be undergoing apoptosis than WT (p=0.026). The frequency of apoptosis among λ5KO B-1b cells was indistinguishable from WT.

### Turnover and self-renewal of λ5KO peritoneal cavity B cells

Cell turnover was evaluated by measuring BrdU incorporation after one week’s exposure to drinking water containing BrdU. As expected, given that Fraction E cells are the product of recent cell division during early B cell development, almost all of the Fraction E immature B cells in both 8-12-week-old WT and λ5KO had incorporated BrdU (**Figure 2C**, **Table 1**). When comparing by PerC B cell subset, BrdU incorporation among B-1a, B-1b, and B-2 cell subsets was statistically indistinguishable between λ5KO and WT mice. Across subsets, the frequency of BrdU incorporation in B-1a cells was twice that of B-1b and three to four times higher than in B-2 cells.

### D_H_ and D_H_ reading frame utilization

**Figure 3:**
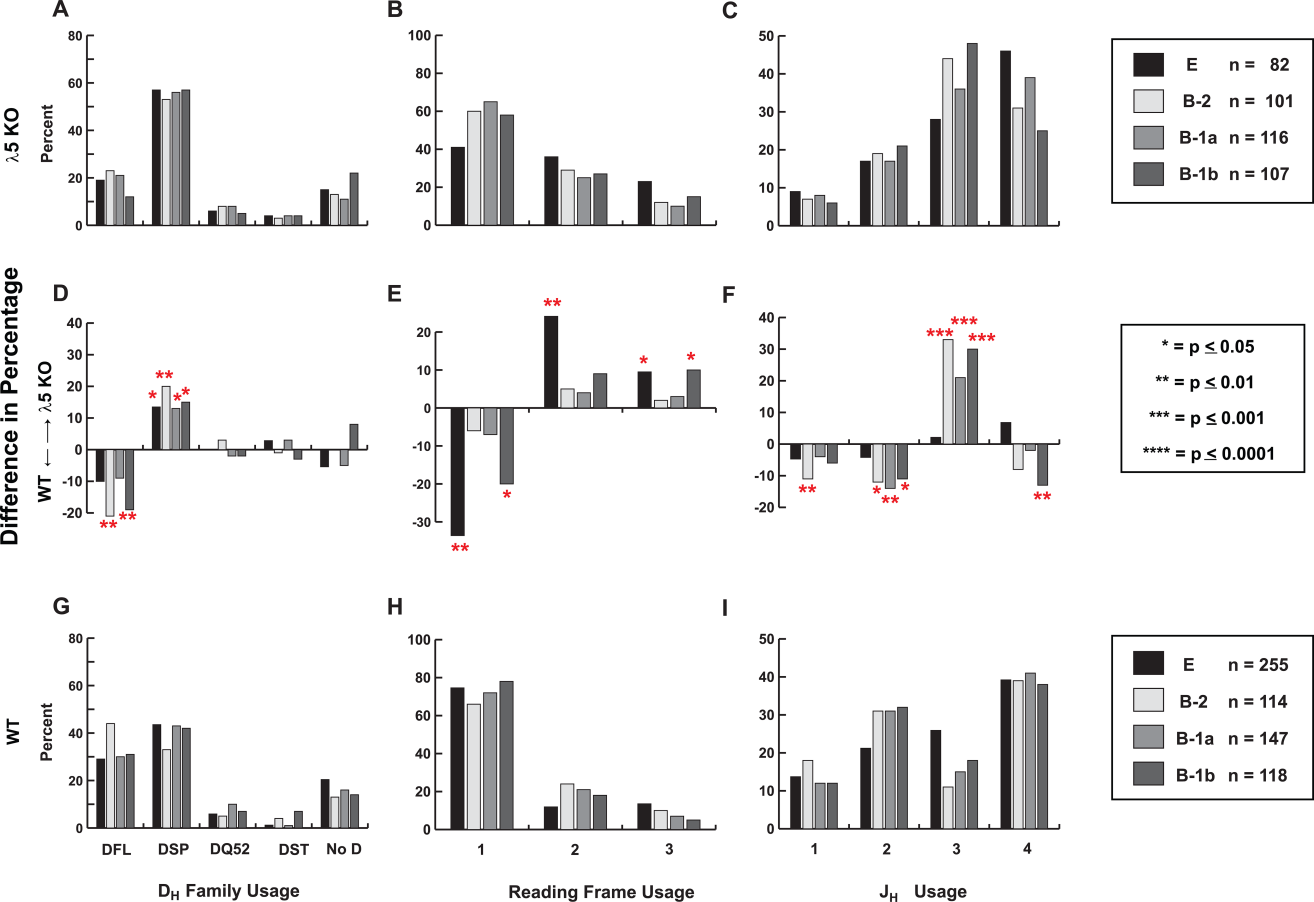
D_H_, D_H_ reading frame, and J_H_ utilization in λ5KO [top, **(A–C)**] versus WT [bottom, **(G–I**)] bone marrow fraction E and peritoneal cavity B1a, B1b, and B2 subsets. The differences in percentage utilization are illustrated in the middle panel **(D–F)**, with the p values indicated by asterisks (right box).

In 8-12-week-old WT Fraction E, the ratio of RF1 to RF2 among DFL-containing sequences was 3:1; it was 8:1 in DSP-containing sequences. In λ5KO Fraction E, abrogation of the preBCR checkpoint was associated with a one-third increase in the prevalence of DSP-containing variable domains compared to WT, with a compensatory one-third decrease in the use of DFL gene segments (**Figures 3A, D, G**). This same shift, favoring DSP over DFL usage, was maintained across all PerC B cell subsets. DQ52, DST, and sequences without an identifiable D gene segment were not significantly affected.

Despite the many termination codons in RF3, the use of RF3-encoded leucine and arginine was also increased in λ5KO fraction E cells and in B1b cells (**Figures 3B, E**). Although the use of RF1 was decreased among immature fraction E λ5KO B cells in the bone marrow, the use of RF1 in PEC λ5KO B-1a and B-1b B cells was statistically indistinguishable from WT (**Figures 3B, E, H**). In contrast, RF1 usage among B-2 cells remained significantly decreased when compared to WT.

### J_H_ utilization

In BALB/c mice, J_H_1, 2, and 4 encode one to two tyrosines at the 5’ end of the coding sequence, which is the portion that contributes to the CDR-H3 loop (**Supplementary Figure 1**). These tyrosine-encoding codons are precisely the focus of the nucleotide microhomology between the 3’ end of the D_H_ and the 5’ end of the J_H_. Microhomology-influenced rearrangement helps drive increased use of RF1 as well as enrichment for tyrosine. J_H_3, which does not share microhomology with the 3’ termini of the D_H_, encodes tryptophan rather than tyrosine at the 5’ terminus.

In WT fraction E cells, there is a stair-step pattern in the use of J_H_ gene segments (**Figure 3I**). J_H_4 is used more frequently than J_H_3, which, in turn, is more frequently used than J_H_2, which is used more frequently than J_H_1. This stair-step pattern is altered in WT B-2, B-1a, and B-1b cells. In all three WT PerC B cell subsets, the use of J_H_3 is reduced when compared to Fraction E (**Figure 3I**).

Interestingly, λ5KO fraction E demonstrates the same stair-step pattern of J_H_ utilization as WT, with J_H_4 > J_H_3 > J_H_2 >J_H_1 (**Figures 3C, F**). However, unlike WT, the use of J_H_3 was increased in λ5KO PEC B-2, B-1a, and B-1b cells, with a compensatory decrease in the use of the other J_H_ segments (**Figures 3C, F**).

### Deconstruction of the CDR-H3 repertoire as a whole

To examine in detail the effect of eliminating preBCR selection on CDR-H3 content at the nucleotide level, we deconstructed each CDR-H3 sequence and compared the various components among all CDR-H3 sequences.

Between 8-12-week-old WT and λ5KO bone marrow immature B cells (fraction E), two differences in the CDR-H3 repertoire achieved statistical significance. First, the length of the retained D_H_ sequence was shorter by two nucleotides in the λ5KO (p=0.02), and second, the λ5KO CDR-H3 repertoire was more hydrophobic (p=0.002), on average, than WT (**Supplementary Table 2**).

Among the three peritoneal B cell subsets, B-2 sequences demonstrated the fewest differences between WT and λ5KO, and B-1b demonstrated the most. The differences among all WT and λ5KO B-2 sequences were limited and subtle. λ5KO CDR-H3s had slightly more 5’ P junction sequence than WT (p=0.01), whereas WT had slightly more 3’ P junction sequence (p=0.04) and more D/J overlap (p=0.0009) than the λ5KO (**Supplementary Table 2**).

**Figure 4:**
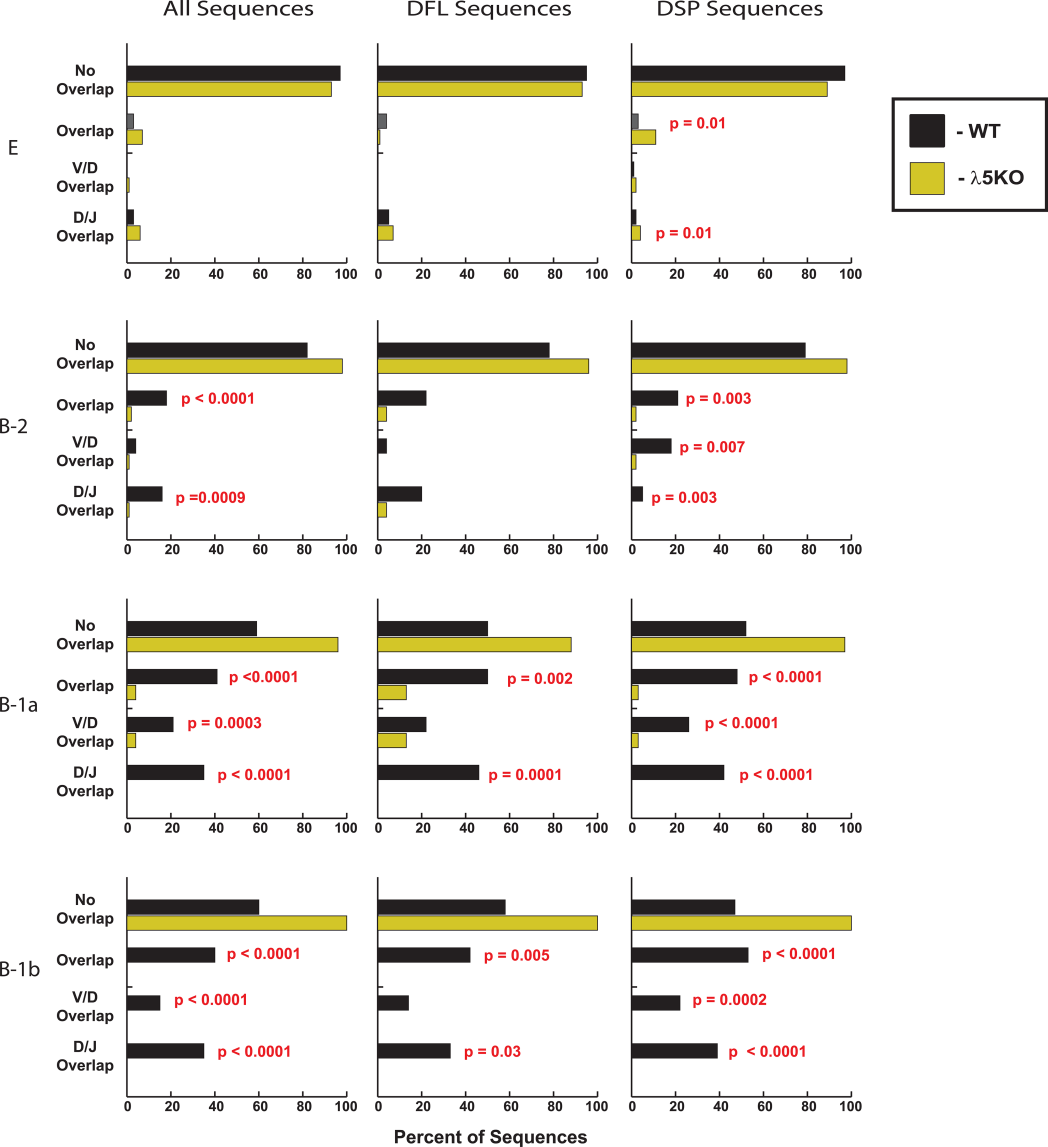
Percentage of CDR-H3 sequences with rearrangements using microhomology between either V and D, or D and J. Shown on the left is the distribution of gene segment overlap among all the sequences in the given B cell subset, regardless of the identity of the D. Shown in the middle and on the right is the distribution of gene segment overlap among CDR-H3 using DFL (middle) or DSP (right) gene segments. Only statistically significant p values (≤ 0.05) are shown (red font).

Among B-1a sequences, the λ5KO had less loss of V_H_ (p=0.01) and J_H_ (p=0.04) terminal sequence (**Supplementary Table 2**). Unexpectedly, more WT sequences retained V/D and D/J overlapping CDR-H3 sequence than λ5KO (p = 0.0003 and p ≤ 0.0001, respectively) (**Supplementary Table 2**, **Figure 4**).

**Figure 5:**
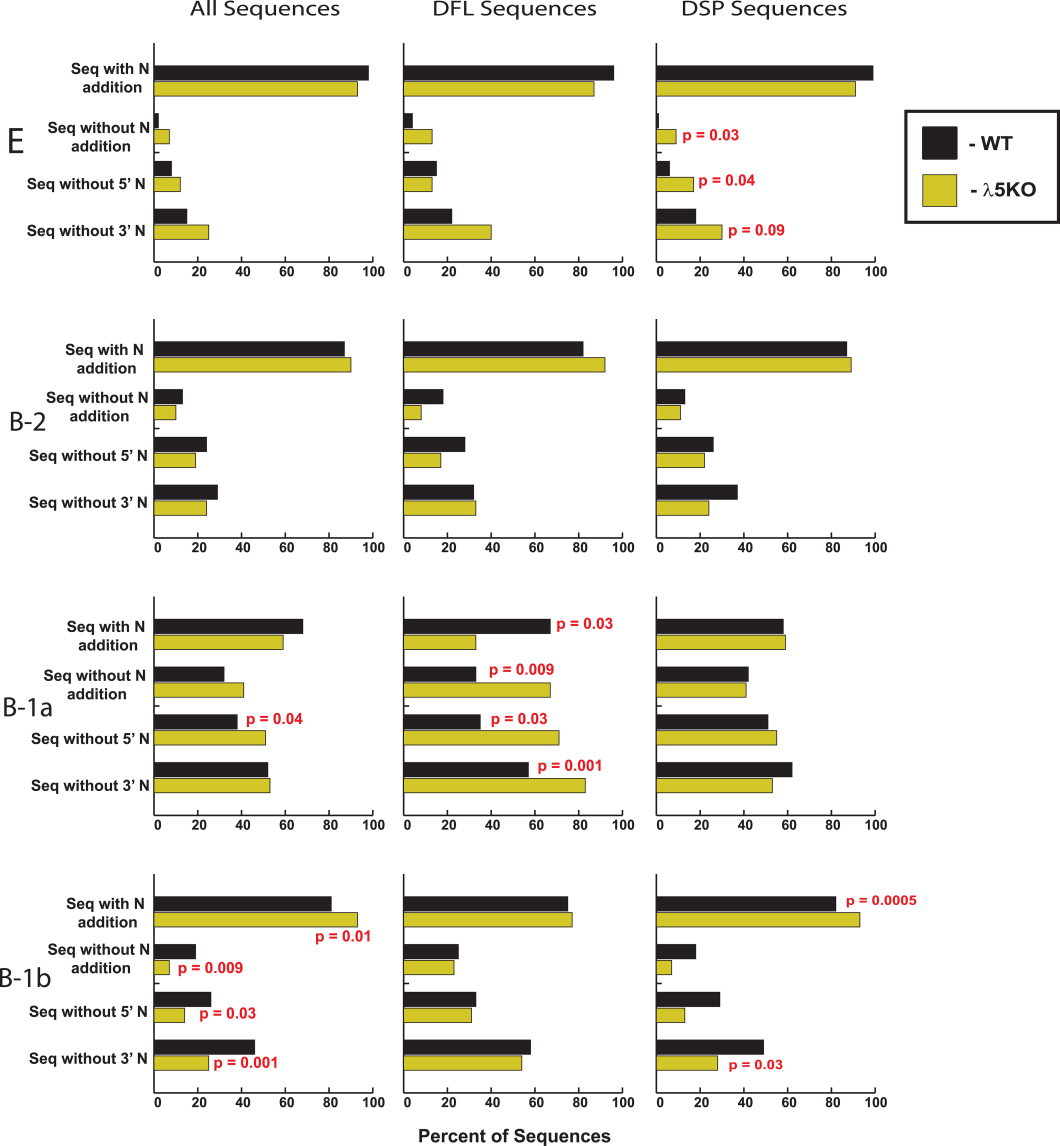
Percentage of CDR-H3 sequences that are devoid of N addition either between V and D (Seq without 5’ N), or D and J (Seq without 3’ N), or both (Seq without N addition). Sequences lacking N addition entirely are more likely to be fetal-derived. Shown on the left is the distribution of sequences lacking N addition among all the sequences in the given B cell subset, regardless of the identity of the D. Shown in the middle and on the right is the distribution of sequences lacking N addition among CDR-H3 using DFL (middle) or DSP (right) gene segments. Student`s t-test was used for statistical analysis. Only statistically significant p values (≤ 0.05) are shown (red font).

The B-1b fraction showed the greatest differences between the two mouse strains. Following the pattern established in fractions E and B-1a, B-1b λ5KO sequences retained less germline DH sequence (p=0.002) and shorter DH length than WT (p=0.0004). B-1b λ5KO sequences also retained more JH P junction sequence than WT (0.04). λ5KO B-1b sequences had less V/D and D/J overlap (p<0.0001) than WT (**Supplementary Table 2**, **Figure 4**). Unexpectedly, they also had more total N addition than WT (p=0.01) (**Supplementary Table 2**, **Figure 5**).

### Detailed comparisons of the DFL and DSP containing CDR-H3 repertoires

We separately examined and compared λ5KO DFL-containing and DSP-containing sequences to WT. We chose to include DH family-specific deconstructions because DFL and DSP-containing CDR-H3 sequences appeared to be affected differently by the lack of λ5, and hence, the absence of preBCR selection.

Statistically, there were no differences in the construction of the CDR-H3 among immature Fraction E WT and λ5KO immature B cells that contain the DFL sequence (**Supplementary Table 3**). When compared to WT, λ5KO DSP-containing CDR-H3 sequences from fraction E exhibited greater loss of germline D_H_ sequence (p=0.008), primarily at the 5’ end (p=0.009). There was enrichment for D/J overlap (p=0.01), which has the effect of maintaining tyrosine-encoding sequence at the 3’ end (carboxy terminus) of the CDR-H3 loop (**Supplementary Table 4**, **Figure 4**).

As in fraction E, B-2 λ5KO CDR-H3 sequences containing DFL codons were more similar to WT than those containing DSP (**Supplementary Table 3**). Among the DFL-containing sequences, there were no statistically significant differences between WT and λ5KO B2 CDR-H3. Among B-1a, λ5KO DFL CDR-H3 sequences exhibited greater 3’ terminal D_H_ sequence loss (p=0.04), less 5’ terminal J_H_ sequence loss (p=0.02), less D/J overlap (p=0.0001) (**Figure 4**), and less N addition (p=0.03) (**Supplementary Table 3**, **Figure 5**). Among B-1b, λ5KO DFL CDR-H3 sequences, there was more D_H_ sequence loss leading to shorter DH length (p=0.04), less total identifiable germline sequence (p=0.03) (**Supplementary Table 3**), and fewer D/J overlap sequences (p=0.03) (**Figure 4**).

Among DSP-containing CDR-H3, λ5KO B-2 sequences had slightly less 3’ D P junction sequence (p=0.04) (**Supplementary Table 4**), and fewer sequences with D/J overlap (p=0.003) (**Figure 4**). In the B-1a population, there were fewer λ5KO sequences with V/D and D/J overlap (p<0.0001) (**Figure 4**), and slightly more J_H_ P junction sequences (p=0.02) (**Supplementary Table 4**). In the B-1b population, there was more loss of 3’D_H_ sequence in the λ5KO (p=0.02) that led to a significant decrease in DH length (p=0.04), as well as more retention of J_H_ P junction sequence (p=0.04) (**Supplementary Table 4**). There were fewer sequences with V/D and D/J overlap (p=0.0002 and p ≤ 0.0001, respectively) (**Figure 4**) and more sequences with N addition between V and D (p=0.04) and between D and J (p=0.0004) (**Supplementary Table 4**).

### Distribution of amino acids at the amino and carboxy termini of CDR-H3

**Figure 6:**
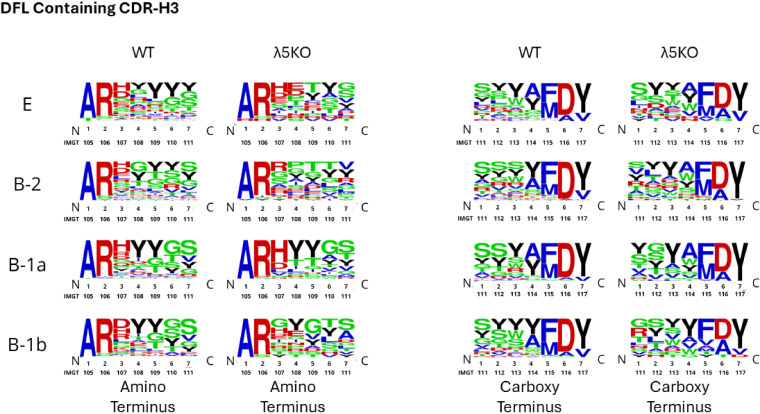
Relative amino acid utilization on a position-by-position basis at the amino and carboxy termini of WT and Λ5KO CDR-H3 sequences using DFL gene segments. Color coding is as described in **Figure 1**.

**Figure 7:**
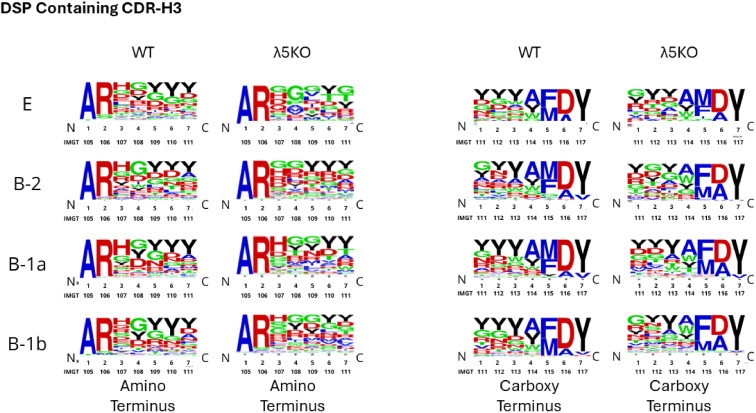
Relative amino acid utilization on a position-by-position basis at the amino and carboxy termini of WT and Λ5KO CDR-H3 sequences using DSP gene segments. Color coding is as described in **Figure 1**.

Categorical selection of the amino acid content of CDR-H3 can be visualized by examining the distribution of amino acids on a position-by-position basis. Because CDR-H3 length varies, we evaluated the amino acid distribution of the first seven amino acids after the cysteine that terminates the Framework Region 3 (FR3) of the V_H_ component of the V domain, and of the last seven amino acids before the phenylalanine that marks the beginning of FR4, which is encoded by the J_H_ gene segment. The contribution of V/D overlap and 5’ N addition is greatest at positions 3 and 4 after FR3 (the amino terminus of CDR-H3). Similarly, the contribution of D/J overlap and 3’ N addition is greatest at positions 1, 2, and 3 of the carboxy terminus of CDR-H3. To facilitate further comparison, this analysis was conducted separately on CDR-H3 sequences using DFL and DSP gene segments. The results are shown in **Figures 6**, **7**, respectively.

WT BALB/c shows a pattern of enrichment for tyrosine at positions 4–7 on the amino terminus and for serine and tyrosine at positions 1–3 on the carboxy terminus of DFL-containing CDR-H3 sequences from Fraction E immature B cells (**Figure 6**). In the absence of preBCR selection, λ5KO Fraction E cells have fewer tyrosines than WT at the amino terminus. However, the carboxy terminus retains enrichment for serine and tyrosine (**Figure 6**). Serine is mostly D_H_ encoded, whereas tyrosine can be contributed by either RF1 terminal D_H_ codons or J_H_ codons (**Supplementary Figure 1**).

At the amino terminus of the DFL sequences, selection for tyrosine varies in intensity by B cell subset. The B-1a subset from λ5KO mice recapitulates a WT-like repertoire. Recapitulation of the WT repertoire in the λ5KO B-1b subset is intermediate, and it is anemic in λ5KO B-2 (**Figure 6**). The similarity of λ5KO to WT in the carboxy terminus showed a similar pattern of B-1a > B-1b > B-2, although the differences were less pronounced (**Figure 6**).

Among DSP-containing sequences, selection for tyrosine among PerC sequences when compared to Fraction E was also apparent. Analysis of both the amino and the carboxy termini revealed that λ5KO B-1a sequences were most likely to match WT, followed by λ5KO B-1b and then by λ5KO B-2 (**Figure 7**). PerC D_H_ DSP-containing sequences appear to have converged more to the WT standard than those containing D_H_ DFL sequences.

## Discussion

B-1 cells, both B-1a and B-1b, in the peritoneal cavity are self-renewing and selected, at least in part, by self-antigen (27–29). Either by serendipity or natural selection, many natural antibodies secreted by B-1 cells offer innate protection against a variety of microorganisms. The B-1a subset is heavily enriched for immunoglobulins with sequences common in fetal life. As noted above, fetal antibodies lack N addition and often undergo rearrangement at sites of gene segment microhomology. Thus, fetal VDJ rearrangement promotes the use of RF1 germline sequence, which suggests less reliance on surrogate light chain selection. This led Hardy’s group to question the role of preBCR signaling in influencing the composition of the PerC B cell repertoire (53, 54).

The cellular self-renewal capacities of B-1 cells and the effects of self-antigen selection on the development of the natural antibody repertoire led to the hypothesis that a WT-like natural antibody repertoire, or even, at a minimum, the fetal component of the natural antibody repertoire, would still be generated in the absence of preBCR selection. For example, selection by self-antigen could promote the survival of fetal-derived B-1a B cells with their characteristic germline-enriched sequence. Alternatively, a stereotypical repertoire could accumulate over time by convergent selection for sequences that had survived the absence of preBCR selection, and thus differed by nucleotide composition, yet still expressed the effects of categorical selection of amino acid content, including those encoding specific structural idiotopes (55). This could be considered convergent evolution in real time.

To test these hypotheses, we began by comparing B cell homeostasis in the peritoneal cavity of 8 to 12-week-old homozygous WT and λ5KO mice. As reported previously (21), abrogation of preBCR formation leads to a 90% reduction in the number of Fraction E B cells. In the peritoneal cavity, there is partial reconstitution of B-2 cell numbers with only a 50% reduction. However, as predicted, the numbers of B-1a and B-1b cells are equivalent to wild-type controls. There is no difference in B cell replication, especially in the B-1a subset, where approximately one in ten cells is the product of recent cell division in both mutant and WT mice. Our findings are consistent with an earlier report of pulse chase BrdU incorporation, where B1a cells of λ5KO mice had BrdU incorporation activity equivalent to WT (56). The changes in cell numbers follow the frequency of apoptosis, which is enhanced in λ5KO Fraction E, less prominent in B-2, equivalent to WT in B-1b, and significantly decreased in λ5KO B-1a. The decreased frequency of apoptosis in B-1a may explain why B-1a cell numbers achieve equivalence to WT, even though fewer cells are being produced.

To test the extent of overlap with fetal-like sequence and to seek evidence of possible convergent selection, we then sampled VDJCµ transcripts from PerC λ5KO B-1a, B-1b, and B-2 cells and compared them to their WT counterparts and to the immature B cell population (Fraction E) developing in the bone marrow. Comparison of these CDR-H3 repertoires as a whole revealed marked differences in the utilization of D_H_ DFL and DSP gene segment family sequences by reading frame. Thus, we also compared the repertoires of sequences containing identifiable DFL and DSP sequences to each other.

Examination of the various repertoires altogether disclosed a number of deviations from the WT standard that could be ascribed to the survival of cells bearing immunoglobulins that are normally eliminated at the preBCR checkpoint. The most striking differences reflected the increased survival of B cells using DSP gene segments in RF2 and RF3 and the increased use of J_H_3. Normally, in WT, there is a 3:1 ratio of RF1 to RF2 in DFL-containing CDR-H3 versus an 8:1 ratio in DSP-containing sequences. The DSP RF2 germline sequence is enriched for hydrophobic amino acids, whereas DFL RF2 contains threonine at its center. Threonine contains a side chain that includes a hydroxyl group, which makes it polar but uncharged and thus more likely to interact with the CDR-H3 sensing site in VpreB and create a functional preBCR. These sequence differences likely explain the greater negative selection for DSP RF2 sequences by preBCR selection. In the absence of the preBCR, immunoglobulin H chains expressing DSP-containing CDR-H3 are allowed to populate the immature B cell population, which is thus depleted of tyrosine and enriched for hydrophobic amino acids. DSP gene segments are shorter than DFL, which explains the decrease in average size and the decrease in the average number of amino acids in the λ5KO CDR-H3 sequences.

In the case of J gene segments, tryptophan-encoding J_H_3 is the only J_H_ that lacks tyrosine, and its usage is increased in the λ5KO PEC B-1a, B-1b, and B-2 B-cell subsets. However, in spite of the increased use of JH3, the final B-1a CDR-H3 repertoire recapitulates enrichment for tyrosine. Together, this further underscores the role of DH and junctional processing in permitting convergence to the WT repertoire pattern in B-1a B cells when preBCR function is disabled.

To reveal the mechanism of genetic control of the peritoneal cavity repertoire in the absence of preBCR selection, we next deconstructed the CDR-H3 sequences, with a focus on features that characterize the fetal repertoire. These include the presence or absence of N nucleotides at the V/D and/or D/J junctions and rearrangement at sites of sequence microhomology.

As noted above, the absence of N addition normally leads to enrichment for sequences with V/D or D/J overlap. Microhomology-influenced gene rearrangement helps limit the diversity of the repertoire and enriches for D_H_ RF1 germline sequence and J_H_ sequences with terminal tyrosines, which in turn enriches for tyrosine at the 3’ end of the CDR-H3 loop. However, unlike in WT, enrichment for N-less CDR-H3s did not occur in λ5KO B-1a or B-1b cells, or even in B-2 cells. Specifically, V/D and D/J overlap in WT B-1a, B-1b, and B-2 CDR-H3 sequences was a frequent finding (**Figure 4**). In contrast, λ5KO B-1a, B-1b, and B-2 cells were virtually devoid of CDR-H3s containing microhomology overlaps, and this was irrespective of the identity of the D_H_.

Among B-1a cells from λ5KO mice, DFL-containing sequences were more likely to lack N addition than WT sequences, and conversely, among B-1b B cells, DSP sequences were less likely to lack N addition than WT sequences (**Figure 5**). Thus, for both the repertoire as a whole and for the repertoires expressed by individual D_H_ families, CDR-H3 sequences from λ5KO mice that lacked N addition had a repertoire that differed from WT at the nucleotide level. This argues against the hypothesis that the fetal component of the B-1 repertoire would remain unchanged in the absence of preBCR selection.

We then examined these sequences at the amino acid level. We compared the amino and carboxy termini of CDR-H3 among sequences that used DFL or DSP sequences. We found that, despite the differences at the nucleotide level, λ5KO B-1a cells had created a repertoire similar to that of WT at the amino acid level. Evidence of convergent selection in the B-1b and B-2 repertoires, in that order, was noted, but was less common than that found among the B-1a repertoire. This mirrors the results of our previous analysis of the anti-phosphorylcholine antibody repertoire in mice with altered D_H_ gene segments (55). To summarize, we found evidence that selection pressure on the composition of the antigen-binding site of immunoglobulin from B-1a cells at the amino acid level was able to recreate evolutionarily selected and preferred WT-like antibody amino acid sequences, and presumably idiotope structures, despite an inability to create the normally preferred germline-encoded nucleotide sequence of their CDR-H3 repertoire.

The ability of convergent selection to preserve the dual roles of natural antibodies in λ5KO mice at tissue and organismal levels remains an unanswered question. These roles include the clean-up of cellular debris derived from dying cells and protection against microbes by binding conserved microbial antigens. Moreover, it should be noted that although the system as a whole appears to be closing in on recapitulating a WT-like B-1a repertoire, the self-antigen and microbial antigen reactivities of this altered repertoire at the secreted immunoglobulin level remain undefined and are a focus of future studies.

In conclusion, our data support the view that the fetal component of the natural antibody repertoire expressed in the peritoneal cavity survives and prospers in the absence of preBCR selection. This work emphasizes the importance of the preB cell receptor in regulating the composition of the antibody repertoire, even in an anatomic location where self-renewal and self-antigen selection would be expected to promote the survival and growth of a WT-like repertoire. In the final analysis, the data instead favor the view that a stereotypical natural antibody repertoire will accumulate in real time by selection for convergent sequences that may differ by nucleotide composition but recapitulate stereotypical amino acid content, and thus the structure, of the antigen-binding site.

## Data Availability

The original contributions presented in the study are included in the article/**Supplementary Material**, further inquiries can be directed to the corresponding author/s.

## Supplementary material

Supplementary Figure 1 D_H_ gene segments by family and reading frame, and J_H_ gene segment sequences. (Top) Expression of the individual amino acids in each reading frame of each D_H_ family. Numbering begins at the amino-terminal end of the D_H_. There are two members of the DFL family, nine of the DSP family, and one of the DST and DQ52 families. (Bottom) Amino acid sequences of the four J_H_ gene segments in BALB/c mice. The amino acids upstream of the base can contribute to the CDR-H3 loop. Green indicates neutral or hydrophilic amino acids, blue hydrophobic amino acids, red charged amino acids, and black indicates tyrosine. Termination codons in RF1 and RF3 are shown by a white X.

Supplementary Table 1 CDR-H3 nucleotide and translated amino acid sequences of PerC B-1a, B-1b, and B-2 transcripts from 8-12-week-old λ5KO BALB/c mice. Sequences from WT bone marrow Fraction E; from WT PerC B-1a, B-1b, and B-2; and from λ5KO bone marrow Fraction E have been previously published (11, 21, 24).

Supplementary Table 2 Deconstruction of all CDR-H3 Sequences. The contribution of retained and lost germline sequence, N addition, P junction addition, and sequence overlap by D_H_ and B cell subset is presented by the average and standard error of the mean for each set of sequences. Only statistically significant p values (≤ 0.05) are shown (red font).

Supplementary Table 3 Deconstruction of DFL CDR-H3 Sequences. The contribution of retained and lost germline sequence, N addition, P junction addition, and sequence overlap in DFL CDR-H3 sequences in B cell subsets is presented by the average and standard error of the mean for each set of sequences. Only statistically significant p values (≤ 0.05) are shown (red font).

Supplementary Table 4 Deconstruction of DSP CDR-H3 Sequences. The contribution of retained and lost germline sequence, N addition, P junction addition, and sequence overlap in DSP CDR-H3 sequences in B cell subsets is presented by the average and standard error of the mean for each set of sequences. Only statistically significant p values (≤ 0.05) are shown (red font).
