# Supplementary figures and images for: Absent the pre-B cell receptor checkpoint, the B-1a immunoglobulin CDR-H3 repertoire normalizes by convergent selection
Source: Front Immunol. 2026 Mar 16;17:1733041. doi: 10.3389/fimmu.2026.1733041 (PMC13033528; doi:10.3389/fimmu.2026.1733041)

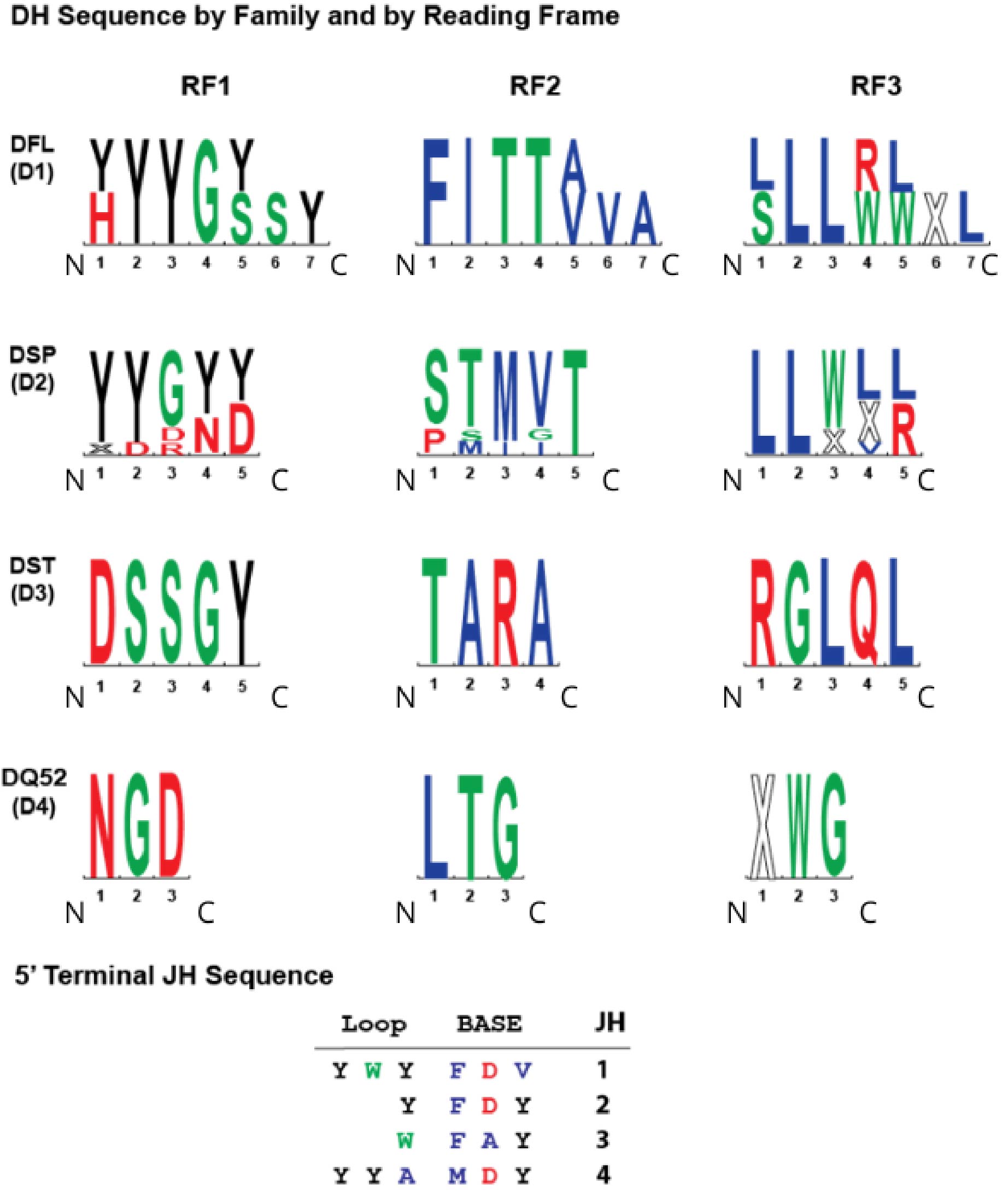

Supplement: Supplementary Figure 1 — DH gene segments by family and reading frame, and JH gene segment sequences. (Top) Expression of the individual amino acids in each reading frame of each DH family. Numbering begins at the amino-terminal end of the DH. There are two members of the DFL family, nine of the DSP family, and one of the DST and DQ52 families. (Bottom) Amino acid sequences of the four JH gene segments in BALB/c mice. The amino acids upstream of the base can contribute to the CDR-H3 loop. Green indicates neutral or hydrophilic amino acids, blue hydrophobic amino acids, red charged amino acids, and black indicates tyrosine. Termination codons in RF1 and RF3 are shown by a white X. [file Image1.jpeg]
