# Supplementary material for: Absent the pre-B cell receptor checkpoint, the B-1a immunoglobulin CDR-H3 repertoire normalizes by convergent selection
Source: Front Immunol. 2026 Mar 16;17:1733041. doi: 10.3389/fimmu.2026.1733041 (PMC13033528; doi:10.3389/fimmu.2026.1733041)
Supplement: Supplementary file 6 [file Table5.docx]

**Analysis of amino acid motifs by CDR-H3 position.**

# Table of Contents

Contents

[I. Table of Contents 1](#_Toc167653280)

[II. Introduction 3](#_Toc167653281)

[A. Hydrophobicity 3](#_Toc167653282)

[III. Sequences 3](#_Toc167653283)

[A. All CDR-H3 3](#_Toc167653284)

[1. Raw Sequences 3](#_Toc167653285)

[2. Counting from the amino terminus end. 27](#_Toc167653286)

[3. Counting from the carboxy terminus end. 48](#_Toc167653287)

[B. DFL-Containing CDR-H3 69](#_Toc167653288)

[1. Raw Sequences 69](#_Toc167653289)

[2. Counting from the amino terminus end. 75](#_Toc167653290)

[3. Counting from the carboxy terminus end. 81](#_Toc167653291)

[C. DSP-Containing CDR-H3 87](#_Toc167653292)

[1. Raw Sequences 87](#_Toc167653293)

[2. Counting from the amino terminus end. 98](#_Toc167653294)

[3. Counting from the carboxy terminus end. 108](#_Toc167653295)

[IV. Figures 118](#_Toc167653296)

[A. All Sequences 118](#_Toc167653297)

[1. Counting from the amino terminus end. 118](#_Toc167653298)

[2. Counting from the Carboxy terminus end. 121](#_Toc167653299)

[B. DFL-Containing Sequences 123](#_Toc167653300)

[1. Counting from the amino terminus end. 123](#_Toc167653301)

[2. Counting from the Carboxy terminus end. 126](#_Toc167653302)

[C. DSP-Containing Sequences 128](#_Toc167653303)

[1. Counting from the amino terminus end. 128](#_Toc167653304)

[2. Counting from the Carboxy terminus end. 130](#_Toc167653305)

# Introduction

**Positions are counted from either the amino or carboxy termini. Sequences are from 8 week old female wild-type and λ5ko BALB/c mice. They are obtained from sorted bone marrow and peritoneal cavity B cells. Motifs are graphically analyzed using WebLogo from a Berkeley server.**

## Hydrophobicity

**
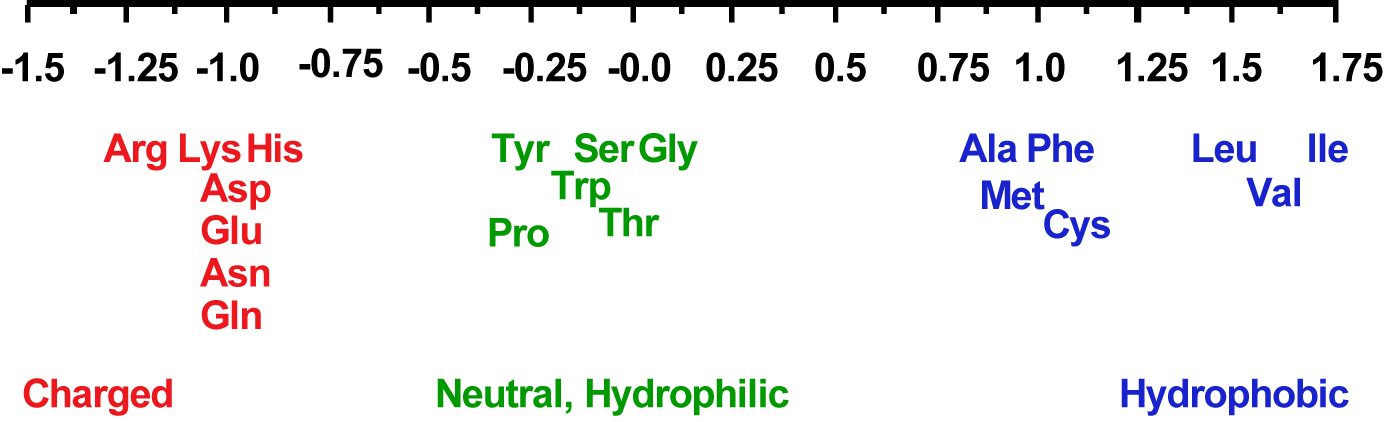
**

**The Kyte-Doolittle hydrophobicity index is used to determine hydrophobicity, as shown above. Colors, except for Tyrosine, are as shown above. Tyrosine is shown in black.**

**WebLogo Parameters**

**RKHDENQ charged, red**

**SGWPT neutral, green**

**AFLIMVC hydrophobic, blue**

# Sequences

## All CDR-H3

### Raw Sequences

##### WT

###### WT Bone Marrow Fraction E

| **TRLLFTADV** |
| --- |
| **ASYYDFDY** |
| **ARHAGRDY** |
| **TRDFHYFAY** |
| **TRDFHYFAY** |
| **ARRGITGYFDY** |
| **ARPSITGAMDY** |
| **TSTTVVAP** |
| **ARHEGLLRPFAY** |
| **ARQNYYEDY** |
| **ASHRYGGFAY** |
| **ARQENYPYAMDY** |
| **ARSLRYAMDY** |
| **ARALLHWYFDV** |
| **ARDHRYGYAMDY** |
| **ARHYYGSSFDY** |
| **ARHALYYGYAMDY** |
| **ARGGVRPFDY** |
| **ARQGLRLFDY** |
| **ARHDYYGSFSMDY** |
| **ARHAYYYGSSPPDY** |
| **ARRVYYYGSSYFDY** |
| **ARTPLITKTPPLFDY** |
| **AREGFTTVVAKMDY** |
| **ATLLRPPFAY** |
| **ARQEGGKGYFDY** |
| **ARHYYGSSYWFDY** |
| **ARHDYYGSSYAMDY** |
| **ARDYYGSPAWFAY** |
| **AGDYYGSSYAMDY** |
| **ARYHGSSYAMDY** |
| **ARFYYYGPYWYFDV** |
| **ARDCSSYGFAY** |
| **ARLLYGSSYGGDY** |
| **ARPSKFITTVVARGFAY** |
| **ARHHYSTATAMDY** |
| **AREATATAMDY** |
| **ARHHYYGSINWYFDV** |
| **ARQSYYYGSSPAWFAY** |
| **ARHGPIYYGSTPWNFDV** |
| **ARDPYGSSYGAMDY** |
| **ARHGSSPYAMDY** |
| **ANYYGSSPGGFAY** |
| **ARSHYYGSSLYAMDY** |
| **ARTGSSFYAMDY** |
| **ARGGYGSSLLYFGV** |
| **ARGYYGYWYFDV** |
| **ARGRVHYYGCYAMDY** |
| **AREFITTVVADWYFDV** |
| **ARGTTATYYFDY** |
| **ARVLRPSDYFDY** |
| **ARHSLLREGLAMDY** |
| **ARDPNYYGSSYPPWFAY** |
| **ARQGHYYGSSWDWYFDV** |
| **AKPYYGSRDYYAMDY** |
| **ARDYYGSRGYYAMDY** |
| **ARANYYGSSYNWYFDV** |
| **TRSYYGSSPYWYFDV** |
| **ARGLYYGSSYLYAMDY** |
| **ASPHYYGRYYAMDY** |
| **ARHYYGYGDAMDY** |
| **TLHYYGYVCAMDY** |
| **ARDRITTARDWYFDV** |
| **AREALRSSDYAMDY** |
| **ARREDYYGSQRDYYAMDY** |
| **ARSRKIDYYGSRAPYWYFDV** |
| **ASGNYYGSSYDYYAMDY** |
| **ARDRRYGSSYVGYAMDY** |
| **ARDPYYGSSYVDWYFDV** |
| **ARHPHYYGSSSLYWYFDV** |
| **ARSPYGSSPELYYLDY** |
| **ARDIYYYGSSSSYYAMDY** |
| **AREGSYYGYVNWYFDV** |
| **ARETSYYYGSSYSLYYAMDY** |
| **ASWGRAY** |
| **AELGQDY** |
| **ARHGTGTWAY** |
| **ARHENWEPFAY** |
| **ARHSNWDGFDY** |
| **ARRNWAYFDV** |
| **ARLPICGRDYFDY** |
| **ARGAGTGFAY** |
| **ARHGVGRYFDY** |
| **ARDTWEGAMDY** |
| **ARQRESGITWFAY** |
| **TKQRTGTGAMDY** |
| **ARGSGTGAMDY** |
| **ARHDWDLYAMDY** |
| **ARQGIYYAMDY** |
| **ASLLLF** |
| **ARLDYYLDY** |
| **ARDCSYYGPFAY** |
| **ARGIYYGNYPN** |
| **ARPLDGYYAY** |
| **AIHYDPFAY** |
| **ASREVYDYFAY** |
| **ARGKGYYCAMDY** |
| **ARGPDSAFAY** |
| **ARSMMVTIDY** |
| **ASPSMITTAY** |
| **AREANLLPYAMDY** |
| **ARLDGYYGFY** |
| **ARTDGFGFAY** |
| **ARHEGNPFAY** |
| **ARERDYYGNSFAY** |
| **ARHEGGNWFAY** |
| **ARHGGYDGTY** |
| **GNDYGFAY** |
| **TRDYYESAMDH** |
| **ARHGYGNYEAY** |
| **ARPEISTMITTSDY** |
| **ARHERWSYYFDY** |
| **ARHEGWLRDFDY** |
| **AREGWLHAMDY** |
| **ARDRRLLLFAY** |
| **ARLGWLRRRNY** |
| **AGTDYRYDGFAY** |
| **ARSDGYYDFDV** |
| **ARQDGNRGFAY** |
| **ARHQIYYGNPWFAY** |
| **ASIYYGNYAVDY** |
| **ASLYGSHAMDY** |
| **ARDRYGYDGFAY** |
| **AREYGYAWFAY** |
| **ARPHDGYYYFDY** |
| **ARRGLIYDGYPWFAY** |
| **ARQRLYYDYDVFDY** |
| **ARHDYRSSWFAY** |
| **AARAYRYDYFDY** |
| **ARCTRAMDY** |
| **ARSMITTGFAY** |
| **ARILMITYAMDY** |
| **APPTIGTTQFAY** |
| **ARQGWLLRAMDY** |
| **TRSVWSYHAMDY** |
| **ARHGRYSYAMDN** |
| **AREDYRYEGAMDY** |
| **ARRDGYSWYFDV** |
| **ARDYDGYLYAMDY** |
| **ARHDGNYPWFAY** |
| **ASRTDYGNTGAMDY** |
| **ARLHYGNPPYFDY** |
| **ARHIGNHWYFDV** |
| **ASLYYGNYEGVDY** |
| **ASGNPAWFAY** |
| **ARGGDYGNFYAMDY** |
| **ARQVGIYYGYDNAMDY** |
| **ARGNYGYLYAMDY** |
| **ARHDGYGYAMDY** |
| **ARHDYDYDEWFAY** |
| **ARYDYDWYFDV** |
| **ARGYDYDAWFAY** |
| **ARHSYYDYDAAMDY** |
| **ARVYDYADYFDY** |
| **ARERAYYGNYEGVAY** |
| **AREKYGNFEYFDV** |
| **ARDNYRYDYAMDY** |
| **ARLPRYDNAMDY** |
| **ARQGYDGAMDY** |
| **ARERGPTMINYYAMDY** |
| **ARNDATMITGYAMDY** |
| **ARGSTMITLYAMDY** |
| **ARHGRRDAMDY** |
| **ARGLRGFAMDY** |
| **ARAYDGYGGWYFDV** |
| **ARAIDGYYEAWFAY** |
| **ARHDYGNYNWYFDV** |
| **ARRGNYPWYFDV** |
| **ARHEGDYGNSFPWSAY** |
| **ASYGNYSYYFDY** |
| **ARHGNYPAWFAY** |
| **ARQGDGNYVSWFAY** |
| **ARHGRYYGNYVYAMDY** |
| **ARSVYYGNYEWYFDV** |
| **ARHSPGTAHYAMDY** |
| **ARHPPYGYDGAHFDY** |
| **ARCGYDGWYFDV** |
| **TRVGDGYEGYAMDY** |
| **ARDRGYDEVWFAY** |
| **ASLIYYDYDVAWFAY** |
| **ARDKDYRYDDYAMDY** |
| **ARWNRYDVKGSDY** |
| **ARGQXYRYPYXAMDY** |
| **ARWRYDVGYFDV** |
| **ARRGITKGYAMDY** |
| **AKWLLRGYAMDY** |
| **AREVRRDGAFDY** |
| **ARDGYYDPAWFAY** |
| **ARPDGYYVDWYFDV** |
| **ARQRGYDERHYFDY** |
| **ARFGYPRYWYFDV** |
| **ARLYYGYVDYYAMDY** |
| **ARYYDYDDYYAMDY** |
| **ARDGDYDGGSLMDY** |
| **ARGRAYGNYDYYAMDY** |
| **ARIWYPGDYYAMDY** |
| **ARGGSYYGNPYYYAMDY** |
| **AREKGNYGFFYAMDY** |
| **ASHYGYDEGYYATDY** |
| **ASPAYYGNYTSPYYAMDY** |
| **ARSPSSMDY** |
| **ARQRQLGLFDY** |
| **ARHSSGPAWFAY** |
| **QMIH** |
| **ARDLAPRY** |
| **ARDPGKLYY** |
| **TRGYH** |
| **ARHDRAY** |
| **ARHAAY** |
| **ARTPSLAY** |
| **ARGPWDY** |
| **ARHLPPDY** |
| **ARTEDY** |
| **ASGLVSPSDY** |
| **ARQRDGLAY** |
| **ARQRGLDY** |
| **ARKRVALDY** |
| **ARDCFFAY** |
| **ARHMDY** |
| **ARPESFDY** |
| **ARWQPRAGMDY** |
| **ARKVFDY** |
| **ARQNSRGFAY** |
| **ARGEGFAY** |
| **VRGWGFAY** |
| **ARGEVGFAY** |
| **ARGFTMDY** |
| **ARPADEGYFDY** |
| **ARYFDY** |
| **ARRAMDY** |
| **ARKYFDV** |
| **ARTDDWFAY** |
| **ARHEGWFAY** |
| **AKGGYWFAY** |
| **ARGGSDATDY** |
| **ARDGVLFPWFAY** |
| **ARHCPYYFDY** |
| **ARQGGYAMDY** |
| **ARGGYPYAMDY** |
| **ARLGAWFAY** |
| **ARRRGYGWYFDV** |
| **ASDRYAMDY** |
| **ARQDWPYAMDY** |
| **ARHRGYSYAMDY** |
| **ASQGGGYYFDY** |
| **ARSPGVRRGYYFDY** |
| **ARHEGVPYAMDY** |
| **AREGVNPAWFAY** |
| **ARKTIPYAMDY** |
| **ARSPLYAMDY** |
| **AGHWYFDV** |
| **ARHGGGEYYFDY** |
| **ARHAYYAMDY** |
| **ARPDYYAMDY** |
| **ARLGDYYAMDY** |

###### WT Peritoneal Cavity B-2

| ARLTTDY |
| --- |
| ARSLGGYSPFDY |
| ARHYYGRDY |
| ARITTVVFDY |
| ASLLLRLRLDY |
| AREANYGSSYDY |
| ARSPYYGSSPDY |
| ARYYYGSSFDY |
| ARLYGSYFDY |
| ARDTTVVAFDY |
| AREGLRLPYFDV |
| AREGLRLRAMDY |
| ARELLRSYAMDY |
| ARHGDYYGSSDFDY |
| ARDSSPFDY |
| ARHYGSSYFDY |
| ARHYYGYWYFDV |
| AVANWAY |
| ARVTTATEAMDY |
| ARHTVVATEDY |
| ARHEGSSSGFAC |
| ARRDYYYYGSSYAMDY |
| ARDYYGSSWYFDV |
| ARDHYYGSSYAMDY |
| ARVYYYGSRRAMDY |
| ARRGSSGAMDY |
| ARCGYYGSPYYFDY |
| ARFYYGSSWYFDV |
| AYYYGSSCAMDY |
| ARSTARNYAMDY |
| ARGRTTAYYYAMDY |
| ARHPYITTVVAEYFDV |
| ARLNSLITTVVANAMDY |
| ASDYGSSHWYFDV |
| ARDGTYYYGSSSYYFDY |
| ASPAYYYGSRGNAMDY |
| ARPGSSYWYFDV |
| ASYGSSYWYFDV |
| ARDYGSSYVYFDY |
| ARSGYGSSYVNFDV |
| ARRGTVVATRYFDY |
| ARGTTVVAHWYFDV |
| ARQGIRRNHSRSFRYFDV |
| ARRKPYGSSYVGAMDY |
| ARNYGSSYVPYFDY |
| ARQGITTVVATRDAMDY |
| ARGITTVVATMYYFDY |
| ARQGEGSSYLRWYFDV |
| ARHWNYGSSYVGGYFDY |
| ARMVATSGRSYFDY |
| ARHPNWDFDY |
| ARDSGTFDY |
| ARQGDWYFDY |
| ARRDWDVKAMDY |
| ARGGTHYAMDY |
| ATNWRTNWRTWFAY |
| ARPDGAY |
| ASYYGNY |
| ARAYYGTAY |
| ARHDYAY |
| AREGMIAMDY |
| ARAIEEAMDY |
| ARHDYRYAWFAY |
| ARGGNYDGDY |
| ARQGYGNWYFDV |
| ARHWDGNYAMDY |
| ARYGYDYFDY |
| ARYGNYGLDY |
| ARHGLYDYDGFAY |
| ARHDGYYAMDY |
| ARRGSTMMEWYFDV |
| ARLMTTTVMDY |
| ARGLRPHFDY |
| ARGVLRYFDV |
| ANLLWLEYAMDY |
| ARHEDYRYDDGFDY |
| ARERNDYDDAMDY |
| ARQRDYAYAMDY |
| ARPWDYGNYYAMDY |
| ARHYGNYYAMDY |
| ARRGDYGNFAWFAY |
| ARVDYDAWFAY |
| ARGNYYWSLYAMDY |
| AAMITTAWFAY |
| ARHGRLPYYAMDY |
| ARQGDGNYGWYFDV |
| ARHGGNYGYAMDY |
| ARYDYDVGYFDV |
| ARGIGNYVRAMDY |
| ARREITGDYAMDY |
| ARRGYDRAYAMDY |
| ARDGYDPYYAMDY |
| ARHGYDDAAWFAY |
| ARRWLLRGNYAMDY |
| ARQGLFDY |
| ASLDSSGYLAY |
| ARQDSSGYDYFDY |
| ARVGLPFYAMDY |
| ARASSGYVDYAMDY |
| ARDGLDY |
| ARGGGFDY |
| ARPLSPSQAMDY |
| ARPSTRSQYFDV |
| ARQRGAMDY |
| ARSQLYFDY |
| ARPAMDY |
| ARKKSDAMDY |
| ARHPYYFDY |
| ARHSGRWYFDV |
| ARGPVYAMDY |
| ARNWYFDV |
| ARHAGSDWYFDV |
| ARGYYAMDY |
| ARDPYPYYYAMDY |

###### WT Peritoneal Cavity B-1a

| ARHKSP |
| --- |
| ATTGF |
| ARWDY |
| ARAGSRPDY |
| ARDDY |
| ARGFDY |
| ARGFDY |
| ANLFAY |
| ARHLFAY |
| ARQRGTGFAY |
| ARGFAY |
| ATGFAY |
| ASIRAMDY |
| AGAMDY |
| ANWDY |
| ASNYFDY |
| AREDAMDY |
| ARSARYFDV |
| ARLTGAY |
| ARAWFAY |
| ARGEGLRFAY |
| ATWYFDV |
| ARSKQYYFDY |
| ARTGFDY |
| ARLTGFAY |
| ARRRYWYFDV |
| ARHHGLAY |
| ARHYGYDY |
| ARSARADY |
| ARHDYDY |
| ARHDYDY |
| ARRGSNWDQDY |
| ARRNYYAMDY |
| ARHDYYAMDY |
| ARHNPYGNSDY |
| ARSYYGVFDY |
| ARWRDYFDY |
| ARSWDYFDY |
| ARHRTTAFDY |
| ARHGNYDY |
| ARGNFAY |
| ARLTSAY |
| ASGTYFDV |
| ARGLRFAY |
| ARLVWYYFDY |
| ARQLGRAMDY |
| AIYYYGSSYY |
| ARPLLHYYGWFAY |
| ARYDYDFDY |
| AIYYGYAMDY |
| ARHDGYYFDY |
| ARRLFTTVAFDY |
| ARHGNAMDY |
| ALSTMITPFDY |
| ARDTGSYYFDY |
| ARTGTYYFDY |
| ARHYYGYVDY |
| ARSELLRLAGSY |
| ARHGEVRWFAY |
| ARLGYWYFDV |
| ARQDGYYFDY |
| ARSGRGFAY |
| ARGYGSRFDY |
| ARQSYGSLFDY |
| ARRYGNSRMDY |
| ARQDRYDAMDY |
| ARYRYDAMDY |
| ARHGNYAMDY |
| ARGDGLTWFAY |
| ARHDYDYFDY |
| ARQRGNAWFAY |
| ARHDGYYAMDY |
| ARDWDNNYFDY |
| ARQGGWGIITVRNYFDY |
| ARWDTTVVAMDY |
| ARHTTVVAMDY |
| ARHGNWYFDV |
| ARHGNYAMDY |
| ARRGTNYAMDY |
| ARHDGSSPLDY |
| ARHYYGYWYFDV |
| ARPNYYGSSYFAY |
| ARYYGSSYFDY |
| ARGAGSYGSSPFDY |
| ARDREYGNYWYFDV |
| ARHYYRYNWYFDV |
| ARHYYRYDWYFDV |
| ARDYYRYDYAMDY |
| ARHGNYWYFDV |
| ARYDYDYAMDY |
| ARGGYGNYDWFAY |
| ARHDGYYYAMDY |
| ARNYGNYAWFAY |
| ARGDGYDYAMDY |
| ARYDYAMDY |
| ARPGNYYAMDY |
| ARDRGLYYGYDGAMDY |
| ARWYGNYYAMDY |
| ARHAYYGNYYAMDY |
| ARGYDGYYYAMDY |
| ARSGYPYAMDY |
| ARHAARATYYFDY |
| ARHYDYAMDY |
| ARHGYDYAMDY |
| ARAVRNYFDY |
| ARHGNYWYFDV |
| ARHGNYWYFGV |
| ARHGNYYAMDY |
| ARSTMITYYAMDY |
| ARRISTMITTGAMDY |
| ARVGLRRKDFDY |
| ARHYYGSSYYFDY |
| ARRYYYGSSYAMDY |
| ARYYYGSSYYFDY |
| ARFYYGSSYAMDY |
| ARDFIYYYGSSYAMDY |
| ARGKRNPSRAMNY |
| ARWAKYGNYEDAMDY |
| ARLYYGNYYWYFDV |
| ARHDGNLFYAMDY |
| ARHYYGSSYYFDY |
| ARHYYGSSYYFDY |
| TRDYYGSSWYFDV |
| ARDYYGSSWYFDV |
| ARHYYGSSWYFDV |
| ARHYYGSSYAMDY |
| ARHYGSSYAMDY |
| ARHYRYDYYAMDY |
| AMTTATGYYFDY |
| AVITTVVAGAMDY |
| ARKLGPGNYYAMDY |
| ARPYYYGSSYWYFDV |
| ARQAYYGSSYWYFDV |
| ARGYGSSYSYFDY |
| ARGYYGSSLYAMDY |
| ARYGNSRYWYFDV |
| AGQKYGNPYYYAMDY |
| AREDYDYFYYYAMDY |
| ARHYDGYYGYYAMDY |
| ARHYYGSSYYAMDY |
| ARRITTVVATRAMDY |
| ARRITTVVATRAMDY |
| ARRITTVVATRAMDY |
| ARHGTTVVIYYAMDY |
| ARLITTVVATRAMDY |
| ARQDWLLWLRRGVSYFDY |
| ARDRGSRTSYYFDY |
| ARLVYYYGSSYYYAMDY |
| ARGYGNYVGYYAMDY |
| ARHYYGSSYYYAMDY |
| ARGYYDVWGYYAMDY |

###### WT Peritoneal Cavity B-1b

| ARHRTY |
| --- |
| AKGVLNY |
| ARLDY |
| AEGFAY |
| ARSRSFDY |
| ASFDY |
| ARHRFAY |
| ARHPGFAY |
| ARHRPYFDY |
| ALPRAMDY |
| ARYDGY |
| ASWDY |
| ARHAMDY |
| ARDGTY |
| ARLAWFAY |
| ARFFPGGYYFDY |
| ARRESYYAMDY |
| ARRNWYLDY |
| ARLNWDGDY |
| ARRGDYYAMDY |
| ARPGSDYYAMDY |
| ARPGHYYAMDY |
| ARPGSSYFDY |
| ARRELGTMDY |
| AGDRGYDYFDY |
| ARRGNGYFAY |
| ARQGRYDAMDY |
| ARSYYGNFDY |
| ARHDGYFDY |
| ARHRGRTMITFAY |
| ARDGPTGTVSSY |
| ARLGRGFAY |
| ARWYGNYFDY |
| ARSYRYAMDY |
| ARFGSYGNYFDY |
| ARPGVYGNPMDY |
| ARGGIYRYYFDY |
| ASLYGYHFDY |
| ARVYDYYAMDY |
| ARHGYYFDY |
| ARDGYSFAY |
| ARGGNWDGYFDV |
| ARITTAYFDY |
| ARQGITTVVADY |
| ARRTMIRAMDY |
| ARGTGWYFDV |
| ARHELRLRFDY |
| AKDSSGYYFDY |
| ARPKGWAMDY |
| ARRENYGHAMDY |
| ARRYYGSSFAY |
| ARGDYGSTGGY |
| ARFYYGSSFDY |
| ARQGRYDGFDY |
| ARGYRYDWFAY |
| AIYYGNYWFAY |
| ARGGNYGFDY |
| ARQDRYVAMDY |
| ARSGYYPMDY |
| ASGNYAMDY |
| ARLYGNYYFDY |
| ARSGYYLMDY |
| ARHRYDAMDY |
| ASNYYFDY |
| ARHEAMVTAWFAY |
| AREVRQAMDY |
| AREGSGIYAMDY |
| ARDHYYGSTWFAY |
| ARRYGSSYFDY |
| ARDYGSSYFDY |
| ARHYYGSSYFDY |
| ARPLLSGSGGFAY |
| ARRDRSYWYFDV |
| ARQGNYDWFAY |
| AREDYDYAMDY |
| ARHGYDWYFDV |
| ARDGAYYRYDGAMDY |
| ARAVYDYPAWFAY |
| ARSVYGNFYAMDY |
| ARQGAARATYYFDY |
| ARHWATGGFDY |
| TMITTYAMDY |
| TMITTYAMDH |
| ARRLEYFDY |
| ARKFTTVDWYFDV |
| ARRGWLRDYAMDY |
| ARHSSGYYYAMDY |
| ARDQDYGSSYWFAY |
| ARDYYGSSWYFDV |
| ARQTYYYGSYWYFDV |
| ANYYGSYWYFDV |
| ARKDYGSVYAMDY |
| ASSSYAMDY |
| ARGAYYGLYYAMDY |
| ARQDGNYGGYFDV |
| ARRGYRSGYYAMDY |
| ARELYGNYYYAMDY |
| ARELYGNYYYAMDN |
| ARHDGYYGSWFAY |
| ARHATVVAYYFDY |
| ARLTTVVATPFDY |
| ARDAMITTGNYFDY |
| ARHYYGYVGYFDY |
| AGDYYGSSYWYFDV |
| ARDYYGSSYWYFDV |
| ARDYGSSYWYFDV |
| ARHPHYYGSSYYAMDY |
| ARPYGSSSYAMDY |
| ARFYDGYYVGYAMDY |
| ARHGYYDYWYFDV |
| SRHYGNYVEYAMDY |
| ARSITTVVDYYAMDY |
| AGLITTVVATRAMDY |
| ARLITTVVATRAMDY |
| ARRGSYDYAMDY |
| ARPYGSSGYWYFDV |
| ARDRGSSQRDYAMDY |
| ARDYGSSYVYYYAMDY |

##### λ5ko

###### λ5ko Bone Marrow Fraction E

| AREEENSFHFDY |
| --- |
| ARLTTVVDY |
| ARHEILRAMDY |
| ARHEVADY |
| ARVPDYYDYAMDY |
| AHITTATWFAY |
| ARHDYYGSSYFAY |
| ARSFYYGSSWFAY |
| ARQRHSLLRLYWYFDV |
| ARAITTVVDAWFAY |
| AREDYGSSYVNFGV |
| ARDPCYGSSGYAMDY |
| ARDGTTAVSYGYFDV |
| ARHNRGSSSYYAMDY |
| RGSSPSYWYFDV |
| ARHGGRDFAY |
| SRRLTGTHYNAMDY |
| ARRLTGTHYYAMDY |
| SRRLTGTHYYAMDY |
| SRRLTGAHYYAMDY |
| ARHLRDGY |
| ARHPTRDY |
| ARDGNY |
| AREGAMIGY |
| ARQVITT |
| ARHLLYFDY |
| ARQGPYYGNLDY |
| ARLDDYAYY |
| ARIFYDGYCYY |
| ARQGMVTTDY |
| ARGGPTMITFAY |
| ARDGDYGNYGAY |
| ARDPGGNYPSY |
| ARHGVRDYLDY |
| ARSMVTTFDY |
| ARHEGVRVRLLAMDY |
| ARQGYDYAWFAY |
| ARHGDYGAMDY |
| ARGGIYGKRAMDY |
| ARSPLYDAMDY |
| ARDALYGYDVIAY |
| ARDPTIGTAWFAY |
| AFMVTEGFAY |
| ARLGITSAMDY |
| AGVMITTAMDY |
| ARGVRDSMDY |
| ARGWSGYAMDY |
| ARRDGGLLLWFAY |
| ASHDGGFAY |
| ARGAYYRYDGAMDY |
| ARTPYGNYGAMDY |
| ARTADLGDYPYAMDY |
| ARPMMVTHWYFDV |
| ARGAMITTAWFAY |
| ARGRGLRRDWFAY |
| ARDGGLRRWYFDV |
| ARHRGLRRPPAMDY |
| ARRGLRYYYAMDY |
| ARRGLPYYYAMDY |
| ARAVRAYYAMDY |
| ARDGRDYDRAAWFAY |
| ARGGTPYYYAMDY |
| ARQEVTTGYYAMDY |
| ARQGVTTGYYAMDY |
| ARQGVTTGCYAMDY |
| ARSMVTGGYYAMDY |
| ARHPARARAMDY |
| ARHGGLLYAMDY |
| ARGSRYYYAMDY |
| ARPIPPG |
| ATLTPY |
| ARVFIGFDY |
| ASMDY |
| ARRLSTWGFAY |
| ARHKAYFDY |
| ARLPSSWFAY |
| ARQDWYFDV |
| ARDNYAMDY |
| ARHGGGNWYFDV |
| ARRFLYYAMDY |
| ARQPNYYAMDY |

###### λ5ko Peritoneal Cavity B-2

| ARHRTY |
| --- |
| AKGVLNY |
| ARLDY |
| AEGFAY |
| ARSRSFDY |
| ASFDY |
| ARHRFAY |
| ARHPGFAY |
| ARHRPYFDY |
| ALPRAMDY |
| ARYDGY |
| ASWDY |
| ARHAMDY |
| ARDGTY |
| ARLAWFAY |
| ARFFPGGYYFDY |
| ARRESYYAMDY |
| ARRNWYLDY |
| ARLNWDGDY |
| ARRGDYYAMDY |
| ARPGSDYYAMDY |
| ARPGHYYAMDY |
| ARPGSSYFDY |
| ARRELGTMDY |
| AGDRGYDYFDY |
| ARRGNGYFAY |
| ARQGRYDAMDY |
| ARSYYGNFDY |
| ARHDGYFDY |
| ARHRGRTMITFAY |
| ARDGPTGTVSSY |
| ARLGRGFAY |
| ARWYGNYFDY |
| ARSYRYAMDY |
| ARFGSYGNYFDY |
| ARPGVYGNPMDY |
| ARGGIYRYYFDY |
| ASLYGYHFDY |
| ARVYDYYAMDY |
| ARHGYYFDY |
| ARDGYSFAY |
| ARGGNWDGYFDV |
| ARITTAYFDY |
| ARQGITTVVADY |
| ARRTMIRAMDY |
| ARGTGWYFDV |
| ARHELRLRFDY |
| AKDSSGYYFDY |
| ARPKGWAMDY |
| ARRENYGHAMDY |
| ARRYYGSSFAY |
| ARGDYGSTGGY |
| ARFYYGSSFDY |
| ARQGRYDGFDY |
| ARGYRYDWFAY |
| AIYYGNYWFAY |
| ARGGNYGFDY |
| ARQDRYVAMDY |
| ARSGYYPMDY |
| ASGNYAMDY |
| ARLYGNYYFDY |
| ARSGYYLMDY |
| ARHRYDAMDY |
| ASNYYFDY |
| ARHEAMVTAWFAY |
| AREVRQAMDY |
| AREGSGIYAMDY |
| ARDHYYGSTWFAY |
| ARRYGSSYFDY |
| ARDYGSSYFDY |
| ARHYYGSSYFDY |
| ARPLLSGSGGFAY |
| ARRDRSYWYFDV |
| ARQGNYDWFAY |
| AREDYDYAMDY |
| ARHGYDWYFDV |
| ARDGAYYRYDGAMDY |
| ARAVYDYPAWFAY |
| ARSVYGNFYAMDY |
| ARQGAARATYYFDY |
| ARHWATGGFDY |
| TMITTYAMDY |
| TMITTYAMDH |
| ARRLEYFDY |
| ARKFTTVDWYFDV |
| ARRGWLRDYAMDY |
| ARHSSGYYYAMDY |
| ARDQDYGSSYWFAY |
| ARDYYGSSWYFDV |
| ARQTYYYGSYWYFDV |
| ANYYGSYWYFDV |
| ARKDYGSVYAMDY |
| ASSSYAMDY |
| ARGAYYGLYYAMDY |
| ARQDGNYGGYFDV |
| ARRGYRSGYYAMDY |
| ARELYGNYYYAMDY |
| ARELYGNYYYAMDN |
| ARHDGYYGSWFAY |
| ARHATVVAYYFDY |
| ARLTTVVATPFDY |
| ARDAMITTGNYFDY |
| ARHYYGYVGYFDY |
| AGDYYGSSYWYFDV |
| ARDYYGSSYWYFDV |
| ARDYGSSYWYFDV |
| ARHPHYYGSSYYAMDY |
| ARPYGSSSYAMDY |
| ARFYDGYYVGYAMDY |
| ARHGYYDYWYFDV |
| SRHYGNYVEYAMDY |
| ARSITTVVDYYAMDY |
| AGLITTVVATRAMDY |
| ARLITTVVATRAMDY |
| ARRGSYDYAMDY |
| ARPYGSSGYWYFDV |
| ARDRGSSQRDYAMDY |
| ARDYGSSYVYYYAMDY |

###### λ5ko Peritoneal Cavity B-1a

| ARHFRV |
| --- |
| ASGLF |
| ARHGKFDY |
| ARQLY |
| GFAY |
| ARQRERGFAY |
| ARHRYFDY |
| ARQRPWFAY |
| ASGYAMDY |
| ARHGNYAMDY |
| ARSTMETY |
| ARLTGFAY |
| ARLYYAMDY |
| ARHGNYY |
| ARLNWEIFY |
| ARGGYWYFDV |
| ARHDYYAMDY |
| ARLYYYAMDY |
| ASVYYAMDY |
| ARHGNFAY |
| ARHYFYAMDY |
| ARLMITTPY |
| ARDIGTFDY |
| ASLGTGAY |
| ARLLWFAY |
| ARWLLRAY |
| ARLGRYFDV |
| ARELGRRFAY |
| ARQRGSSGYEAY |
| ARDLHYYGYFDY |
| ARHGNAMDY |
| ARGYYDYDFDY |
| ASPWGDYAFAY |
| ARGDGYLLDY |
| ARGSYYGNYVAY |
| ARHWDYAMDY |
| ARHITTVVVDY |
| ARHRGITTVVFAY |
| ARDSMVTTMDY |
| ARHMVTTFAY |
| ARSTPSMVIYFDY |
| MVTTFAY |
| ARTIGTTFAY |
| ARQLTGTWYFDV |
| ARSQLGLRFAY |
| ARHGLRRFAY |
| ARPRPFAY |
| ARWLLTMDY |
| ARLGPAWFAY |
| ARDYYGYAMDY |
| ARHYYGSAMDY |
| ARHYYGSSFDY |
| ARDYYGYWFAY |
| ARDYYGSSFDY |
| ARDGYYAMDY |
| ARGGIYGKRAMDY |
| ARRYGKRAMDY |
| ARHDYDWFAY |
| ARQRGNYAFAY |
| ARDDGYYAMDY |
| ARETNYYFLYAMDY |
| ARSMIYYDYSWFAY |
| ARHIITTVVGFDY |
| ARPEMVTAWFAY |
| ARHVVTTAMDY |
| ARHRGIYAMDY |
| ARSMVTAWFAY |
| ARHARLRQGFAY |
| ARHGRLRRAFAY |
| ARLGRYYAMDY |
| ARQEEDSSGYYAMDY |
| ARHGNYYAMDY |
| ARHYGYDYAMDY |
| ARDGNYWYFDV |
| ARVYDGYGAWFAY |
| ARHANDYDYAMDY |
| ARLHYDYDYAMDY |
| ARDRYDYAMDY |
| ARYRYDYAMDY |
| ARHRYDYAMDY |
| ARHYRYYYAMDY |
| ARHGNYWYFDV |
| ARHGYDWYFDV |
| ARHGNYWYFDV |
| ARPPYYDYLAWFAY |
| ATYYGNYYAMDY |
| ARGDGYYYAMDY |
| ARYGYDWCFDV |
| ARDGNFAWFAY |
| ARHTTATYYFDY |
| ARHTTATAWFAY |
| ARLTTVVAWFAY |
| ARVTTVVATFAY |
| ARHTMVTTYYFDY |
| ARQTIGTTRAMDY |
| ARSTMITTYYFDY |
| ARHTRVRYYAMDY |
| ARHYGSSYYFDY |
| ARHYYGSSYYFDY |
| ARHPPYYYGSSYAMDY |
| ARHYYYGSSYAMDY |
| ARHYYGSSYAMDY |
| ARYAFAYYRYDGGYFDY |
| ARHERYDEAWFAY |
| ARPSLYRYDGAWFAY |
| ARDYDYDLAWFAY |
| ARHRGATGRVFAY |
| ARDGTTPKNAMDY |
| ARHYYGSSYYAMDY |
| ARHYRYDDYYAMDY |
| ARHGYDDYWYFDV |
| ARHPQTLYDYDGGAWFAY |
| ARHEGRYDVFYAMDY |
| ARQCILRLQGYYAMDY |
| ARHYYGSSYYYAMDY |
| ADGSSWGYAMDY |

###### λ5ko Peritoneal Cavity B-1b

| ARHKAY |
| --- |
| ARRLKAY |
| ARHAY |
| ARHYFAY |
| ARSPFDY |
| ARPGPFAY |
| ARAGFAY |
| ASGVRFAY |
| ASKDRFAY |
| ARHKAYFDY |
| ARWPTGAMDY |
| ARGDYFDY |
| ARRAIGT |
| ASHPAWFAY |
| ARPTSPWYFDV |
| ARAWFAY |
| ARRGY |
| AREVAYAMDY |
| ARLYAMDY |
| ARFYDGYY |
| ARDNYAMDY |
| ARHGSMIFAY |
| ASSSTMITY |
| ARWYYAMDY |
| ASSYYAMDY |
| ARHGTPVRYYAMDY |
| ARIHYYAMDY |
| ARHDYYAMDY |
| ARSGPYYYAMDY |
| ARSRGLLAWFAY |
| ARPNYYGYAY |
| ARGYGNFDY |
| AAYGNFDY |
| ARGNYPYFDV |
| ARSRGYYYGSSDY |
| ARGEVTTVVDY |
| ARHPATAY |
| ARSGRSTMIPFAY |
| ARSIPNLMVNFDY |
| ARSIPNLMVNFGY |
| ARRGTGPWFAY |
| ARVLVWYLFDY |
| ARRLLWYYFDY |
| ARQARGRGFAY |
| ARQRGPGFAY |
| ARDWDYDYWFAY |
| ARGGDYVFAY |
| ARFYEGAFDD |
| ARHDYGFAY |
| ARGYYDYDFDY |
| ARSRDGYYFDY |
| ARWEVQFAY |
| ARGTARARFAY |
| AQTARSWFAY |
| ASTTVVATD |
| MMGSAMDY |
| ARQRDGLRGFAY |
| ARLGRDWFAY |
| ARHDYRYAWFAY |
| AREEGYGYPWFAY |
| ASNGNYVFAY |
| ARGYDAMDY |
| ARGGIYGKRAMDY |
| ASGNYDFDY |
| ARHKYGNYEFAY |
| ASYYGNRGFAY |
| ARRGDDYEFFDY |
| ARSGYRSPWFAY |
| ARKGISDYPWFAY |
| ARSEGYGNYVFDY |
| ARWDGLYAMDY |
| ARRITTAVYFDY |
| TREAMVNYAMDY |
| ARHWGVTPWFAY |
| ARHSMVTSWFAY |
| ARIDMIRGAMDY |
| ARGKELLLFAY |
| ARSGRGAMDY |
| ARHYGSSYFDY |
| ARGSSYVAY |
| ARYYGLDWFAY |
| ARRDYGNFWYFDV |
| ARHYGNRYAMDY |
| ARDRSYGNLAWFAY |
| ARDLGYGNLAWFAY |
| ARSGNGYALYFDY |
| ARSYSMVTSYYFDY |
| ARLWLPPYAMDY |
| ALLRRYAMDY |
| ARDYYGSYWYFDV |
| ARDTALGNYPAWFAY |
| AANYDGAWFAY |
| ARHPLYDGYYVGAMDY |
| ARNYRSHYYAMDY |
| ARHGGGTTDDGGAY |
| ARMVKRVAWFAY |
| ARRIGTTRVAMDY |
| ARLRRRAWFAY |
| ARHGGLRRSYAMDY |
| ARHGGLRRSYAMGY |
| ARGGDHSRSYYFDY |
| ARRDDYDEKAWFAY |
| ARNYRYDGGYYFDY |
| ARATMVTTGYWYFDV |
| AKWLRRGYYAMDY |
| AREYGSSRGWYFDV |
| ARIEGYYYGSSLYYYAMDY |

### Counting from the amino terminus end.

##### WT

###### WT Bone Marrow Fraction E Amino terminus

**TRLLFTA**

**ASYYDFD**

**ARHAGRD**

**TRDFHYF**

**TRDFHYF**

**ARRGITG**

**ARPSITG**

**TSTTVVA**

**ARHEGLL**

**ARQNYYE**

**ASHRYGG**

**ARQENYP**

**ARSLRYA**

**ARALLHW**

**ARDHRYG**

**ARHYYGS**

**ARHALYY**

**ARGGVRP**

**ARQGLRL**

**ARHDYYG**

**ARHAYYY**

**ARRVYYY**

**ARTPLIT**

**AREGFTT**

**ATLLRPP**

**ARQEGGK**

**ARHYYGS**

**ARHDYYG**

**ARDYYGS**

**AGDYYGS**

**ARYHGSS**

**ARFYYYG**

**ARDCSSY**

**ARLLYGS**

**ARPSKFI**

**ARHHYST**

**AREATAT**

**ARHHYYG**

**ARQSYYY**

**ARHGPIY**

**ARDPYGS**

**ARHGSSP**

**ANYYGSS**

**ARSHYYG**

**ARTGSSF**

**ARGGYGS**

**ARGYYGY**

**ARGRVHY**

**AREFITT**

**ARGTTAT**

**ARVLRPS**

**ARHSLLR**

**ARDPNYY**

**ARQGHYY**

**AKPYYGS**

**ARDYYGS**

**ARANYYG**

**TRSYYGS**

**ARGLYYG**

**ASPHYYG**

**ARHYYGY**

**TLHYYGY**

**ARDRITT**

**AREALRS**

**ARREDYY**

**ARSRKID**

**ASGNYYG**

**ARDRRYG**

**ARDPYYG**

**ARHPHYY**

**ARSPYGS**

**ARDIYYY**

**AREGSYY**

**ARETSYY**

**ASWGRAY**

**AELGQDY**

**ARHGTGT**

**ARHENWE**

**ARHSNWD**

**ARRNWAY**

**ARLPICG**

**ARGAGTG**

**ARHGVGR**

**ARDTWEG**

**ARQRESG**

**TKQRTGT**

**ARGSGTG**

**ARHDWDL**

**ARQGIYY**

**ASLLLF-**

**ARLDYYL**

**ARDCSYY**

**ARGIYYG**

**ARPLDGY**

**AIHYDPF**

**ASREVYD**

**ARGKGYY**

**ARGPDSA**

**ARSMMVT**

**ASPSMIT**

**AREANLL**

**ARLDGYY**

**ARTDGFG**

**ARHEGNP**

**ARERDYY**

**ARHEGGN**

**ARHGGYD**

**GNDYGFA**

**TRDYYES**

**ARHGYGN**

**ARPEIST**

**ARHERWS**

**ARHEGWL**

**AREGWLH**

**ARDRRLL**

**ARLGWLR**

**AGTDYRY**

**ARSDGYY**

**ARQDGNR**

**ARHQIYY**

**ASIYYGN**

**ASLYGSH**

**ARDRYGY**

**AREYGYA**

**ARPHDGY**

**ARRGLIY**

**ARQRLYY**

**ARHDYRS**

**AARAYRY**

**ARCTRAM**

**ARSMITT**

**ARILMIT**

**APPTIGT**

**ARQGWLL**

**TRSVWSY**

**ARHGRYS**

**AREDYRY**

**ARRDGYS**

**ARDYDGY**

**ARHDGNY**

**ASRTDYG**

**ARLHYGN**

**ARHIGNH**

**ASLYYGN**

**ASGNPAW**

**ARGGDYG**

**ARQVGIY**

**ARGNYGY**

**ARHDGYG**

**ARHDYDY**

**ARYDYDW**

**ARGYDYD**

**ARHSYYD**

**ARVYDYA**

**ARERAYY**

**AREKYGN**

**ARDNYRY**

**ARLPRYD**

**ARQGYDG**

**ARERGPT**

**ARNDATM**

**ARGSTMI**

**ARHGRRD**

**ARGLRGF**

**ARAYDGY**

**ARAIDGY**

**ARHDYGN**

**ARRGNYP**

**ARHEGDY**

**ASYGNYS**

**ARHGNYP**

**ARQGDGN**

**ARHGRYY**

**ARSVYYG**

**ARHSPGT**

**ARHPPYG**

**ARCGYDG**

**TRVGDGY**

**ARDRGYD**

**ASLIYYD**

**ARDKDYR**

**ARWNRYD**

**ARGQXYR**

**ARWRYDV**

**ARRGITK**

**AKWLLRG**

**AREVRRD**

**ARDGYYD**

**ARPDGYY**

**ARQRGYD**

**ARFGYPR**

**ARLYYGY**

**ARYYDYD**

**ARDGDYD**

**ARGRAYG**

**ARIWYPG**

**ARGGSYY**

**AREKGNY**

**ASHYGYD**

**ASPAYYG**

**ARSPSSM**

**ARQRQLG**

**ARHSSGP**

**QMIH---**

**ARDLAPR**

**ARDPGKL**

**TRGYH--**

**ARHDRAY**

**ARHAAY-**

**ARTPSLA**

**ARGPWDY**

**ARHLPPD**

**ARTEDY-**

**ASGLVSP**

**ARQRDGL**

**ARQRGLD**

**ARKRVAL**

**ARDCFFA**

**ARHMDY-**

**ARPESFD**

**ARWQPRA**

**ARKVFDY**

**ARQNSRG**

**ARGEGFA**

**VRGWGFA**

**ARGEVGF**

**ARGFTMD**

**ARPADEG**

**ARYFDY-**

**ARRAMDY**

**ARKYFDV**

**ARTDDWF**

**ARHEGWF**

**AKGGYWF**

**ARGGSDA**

**ARDGVLF**

**ARHCPYY**

**ARQGGYA**

**ARGGYPY**

**ARLGAWF**

**ARRRGYG**

**ASDRYAM**

**ARQDWPY**

**ARHRGYS**

**ASQGGGY**

**ARSPGVR**

**ARHEGVP**

**AREGVNP**

**ARKTIPY**

**ARSPLYA**

**AGHWYFD**

**ARHGGGE**

**ARHAYYA**

**ARPDYYA**

**ARLGDYY**

###### WT Peritoneal Cavity B-2 Amino terminus

**ARLTTDY**

**ARSLGGY**

**ARHYYGR**

**ARITTVV**

**ASLLLRL**

**AREANYG**

**ARSPYYG**

**ARYYYGS**

**ARLYGSY**

**ARDTTVV**

**AREGLRL**

**AREGLRL**

**ARELLRS**

**ARHGDYY**

**ARDSSPF**

**ARHYGSS**

**ARHYYGY**

**AVANWAY**

**ARVTTAT**

**ARHTVVA**

**ARHEGSS**

**ARRDYYY**

**ARDYYGS**

**ARDHYYG**

**ARVYYYG**

**ARRGSSG**

**ARCGYYG**

**ARFYYGS**

**AYYYGSS**

**ARSTARN**

**ARGRTTA**

**ARHPYIT**

**ARLNSLI**

**ASDYGSS**

**ARDGTYY**

**ASPAYYY**

**ARPGSSY**

**ASYGSSY**

**ARDYGSS**

**ARSGYGS**

**ARRGTVV**

**ARGTTVV**

**ARQGIRR**

**ARRKPYG**

**ARNYGSS**

**ARQGITT**

**ARGITTV**

**ARQGEGS**

**ARHWNYG**

**ARMVATS**

**ARHPNWD**

**ARDSGTF**

**ARQGDWY**

**ARRDWDV**

**ARGGTHY**

**ATNWRTN**

**ARPDGAY**

**ASYYGNY**

**ARAYYGT**

**ARHDYAY**

**AREGMIA**

**ARAIEEA**

**ARHDYRY**

**ARGGNYD**

**ARQGYGN**

**ARHWDGN**

**ARYGYDY**

**ARYGNYG**

**ARHGLYD**

**ARHDGYY**

**ARRGSTM**

**ARLMTTT**

**ARGLRPH**

**ARGVLRY**

**ANLLWLE**

**ARHEDYR**

**ARERNDY**

**ARQRDYA**

**ARPWDYG**

**ARHYGNY**

**ARRGDYG**

**ARVDYDA**

**ARGNYYW**

**AAMITTA**

**ARHGRLP**

**ARQGDGN**

**ARHGGNY**

**ARYDYDV**

**ARGIGNY**

**ARREITG**

**ARRGYDR**

**ARDGYDP**

**ARHGYDD**

**ARRWLLR**

**ARQGLFD**

**ASLDSSG**

**ARQDSSG**

**ARVGLPF**

**ARASSGY**

**ARDGLDY**

**ARGGGFD**

**ARPLSPS**

**ARPSTRS**

**ARQRGAM**

**ARSQLYF**

**ARPAMDY**

**ARKKSDA**

**ARHPYYF**

**ARHSGRW**

**ARGPVYA**

**ARNWYFD**

**ARHAGSD**

**ARGYYAM**

**ARDPYPY**

###### WT Peritoneal Cavity B-1a Amino terminus

**ARHKSP-**

**ATTGF--**

**ARWDY--**

**ARAGSRP**

**ARDDY--**

**ARGFDY-**

**ARGFDY-**

**ANLFAY-**

**ARHLFAY**

**ARQRGTG**

**ARGFAY-**

**ATGFAY-**

**ASIRAMD**

**AGAMDY-**

**ANWDY--**

**ASNYFDY**

**AREDAMD**

**ARSARYF**

**ARLTGAY**

**ARAWFAY**

**ARGEGLR**

**ATWYFDV**

**ARSKQYY**

**ARTGFDY**

**ARLTGFA**

**ARRRYWY**

**ARHHGLA**

**ARHYGYD**

**ARSARAD**

**ARHDYDY**

**ARHDYDY**

**ARRGSNW**

**ARRNYYA**

**ARHDYYA**

**ARHNPYG**

**ARSYYGV**

**ARWRDYF**

**ARSWDYF**

**ARHRTTA**

**ARHGNYD**

**ARGNFAY**

**ARLTSAY**

**ASGTYFD**

**ARGLRFA**

**ARLVWYY**

**ARQLGRA**

**AIYYYGS**

**ARPLLHY**

**ARYDYDF**

**AIYYGYA**

**ARHDGYY**

**ARRLFTT**

**ARHGNAM**

**ALSTMIT**

**ARDTGSY**

**ARTGTYY**

**ARHYYGY**

**ARSELLR**

**ARHGEVR**

**ARLGYWY**

**ARQDGYY**

**ARSGRGF**

**ARGYGSR**

**ARQSYGS**

**ARRYGNS**

**ARQDRYD**

**ARYRYDA**

**ARHGNYA**

**ARGDGLT**

**ARHDYDY**

**ARQRGNA**

**ARHDGYY**

**ARDWDNN**

**ARQGGWG**

**ARWDTTV**

**ARHTTVV**

**ARHGNWY**

**ARHGNYA**

**ARRGTNY**

**ARHDGSS**

**ARHYYGY**

**ARPNYYG**

**ARYYGSS**

**ARGAGSY**

**ARDREYG**

**ARHYYRY**

**ARHYYRY**

**ARDYYRY**

**ARHGNYW**

**ARYDYDY**

**ARGGYGN**

**ARHDGYY**

**ARNYGNY**

**ARGDGYD**

**ARYDYAM**

**ARPGNYY**

**ARDRGLY**

**ARWYGNY**

**ARHAYYG**

**ARGYDGY**

**ARSGYPY**

**ARHAARA**

**ARHYDYA**

**ARHGYDY**

**ARAVRNY**

**ARHGNYW**

**ARHGNYW**

**ARHGNYY**

**ARSTMIT**

**ARRISTM**

**ARVGLRR**

**ARHYYGS**

**ARRYYYG**

**ARYYYGS**

**ARFYYGS**

**ARDFIYY**

**ARGKRNP**

**ARWAKYG**

**ARLYYGN**

**ARHDGNL**

**ARHYYGS**

**ARHYYGS**

**TRDYYGS**

**ARDYYGS**

**ARHYYGS**

**ARHYYGS**

**ARHYGSS**

**ARHYRYD**

**AMTTATG**

**AVITTVV**

**ARKLGPG**

**ARPYYYG**

**ARQAYYG**

**ARGYGSS**

**ARGYYGS**

**ARYGNSR**

**AGQKYGN**

**AREDYDY**

**ARHYDGY**

**ARHYYGS**

**ARRITTV**

**ARRITTV**

**ARRITTV**

**ARHGTTV**

**ARLITTV**

**ARQDWLL**

**ARDRGSR**

**ARLVYYY**

**ARGYGNY**

**ARHYYGS**

**ARGYYDV**

###### WT Peritoneal Cavity B-1b Amino terminus

**ARHRTY-**

**AKGVLNY**

**ARLDY--**

**AEGFAY-**

**ARSRSFD**

**ASFDY--**

**ARHRFAY**

**ARHPGFA**

**ARHRPYF**

**ALPRAMD**

**ARYDGY-**

**ASWDY--**

**ARHAMDY**

**ARDGTY-**

**ARLAWFA**

**ARFFPGG**

**ARRESYY**

**ARRNWYL**

**ARLNWDG**

**ARRGDYY**

**ARPGSDY**

**ARPGHYY**

**ARPGSSY**

**ARRELGT**

**AGDRGYD**

**ARRGNGY**

**ARQGRYD**

**ARSYYGN**

**ARHDGYF**

**ARHRGRT**

**ARDGPTG**

**ARLGRGF**

**ARWYGNY**

**ARSYRYA**

**ARFGSYG**

**ARPGVYG**

**ARGGIYR**

**ASLYGYH**

**ARVYDYY**

**ARHGYYF**

**ARDGYSF**

**ARGGNWD**

**ARITTAY**

**ARQGITT**

**ARRTMIR**

**ARGTGWY**

**ARHELRL**

**AKDSSGY**

**ARPKGWA**

**ARRENYG**

**ARRYYGS**

**ARGDYGS**

**ARFYYGS**

**ARQGRYD**

**ARGYRYD**

**AIYYGNY**

**ARGGNYG**

**ARQDRYV**

**ARSGYYP**

**ASGNYAM**

**ARLYGNY**

**ARSGYYL**

**ARHRYDA**

**ASNYYFD**

**ARHEAMV**

**AREVRQA**

**AREGSGI**

**ARDHYYG**

**ARRYGSS**

**ARDYGSS**

**ARHYYGS**

**ARPLLSG**

**ARRDRSY**

**ARQGNYD**

**AREDYDY**

**ARHGYDW**

**ARDGAYY**

**ARAVYDY**

**ARSVYGN**

**ARQGAAR**

**ARHWATG**

**TMITTYA**

**TMITTYA**

**ARRLEYF**

**ARKFTTV**

**ARRGWLR**

**ARHSSGY**

**ARDQDYG**

**ARDYYGS**

**ARQTYYY**

**ANYYGSY**

**ARKDYGS**

**ASSSYAM**

**ARGAYYG**

**ARQDGNY**

**ARRGYRS**

**ARELYGN**

**ARELYGN**

**ARHDGYY**

**ARHATVV**

**ARLTTVV**

**ARDAMIT**

**ARHYYGY**

**AGDYYGS**

**ARDYYGS**

**ARDYGSS**

**ARHPHYY**

**ARPYGSS**

**ARFYDGY**

**ARHGYYD**

**SRHYGNY**

**ARSITTV**

**AGLITTV**

**ARLITTV**

**ARRGSYD**

**ARPYGSS**

**ARDRGSS**

**ARDYGSS**

##### λ5ko

###### λ5ko Bone Marrow Fraction E Amino terminus

**AREEENS**

**ARLTTVV**

**ARHEILR**

**ARHEVAD**

**ARVPDYY**

**AHITTAT**

**ARHDYYG**

**ARSFYYG**

**ARQRHSL**

**ARAITTV**

**AREDYGS**

**ARDPCYG**

**ARDGTTA**

**ARHNRGS**

**RGSSPSY**

**ARHGGRD**

**SRRLTGT**

**ARRLTGT**

**SRRLTGT**

**SRRLTGA**

**ARHLRDG**

**ARHPTRD**

**ARDGNY-**

**AREGAMI**

**ARQVITT**

**ARHLLYF**

**ARQGPYY**

**ARLDDYA**

**ARIFYDG**

**ARQGMVT**

**ARGGPTM**

**ARDGDYG**

**ARDPGGN**

**ARHGVRD**

**ARSMVTT**

**ARHEGVR**

**ARQGYDY**

**ARHGDYG**

**ARGGIYG**

**ARSPLYD**

**ARDALYG**

**ARDPTIG**

**AFMVTEG**

**ARLGITS**

**AGVMITT**

**ARGVRDS**

**ARGWSGY**

**ARRDGGL**

**ASHDGGF**

**ARGAYYR**

**ARTPYGN**

**ARTADLG**

**ARPMMVT**

**ARGAMIT**

**ARGRGLR**

**ARDGGLR**

**ARHRGLR**

**ARRGLRY**

**ARRGLPY**

**ARAVRAY**

**ARDGRDY**

**ARGGTPY**

**ARQEVTT**

**ARQGVTT**

**ARQGVTT**

**ARSMVTG**

**ARHPARA**

**ARHGGLL**

**ARGSRYY**

**ARPIPPG**

**ATLTPY-**

**ARVFIGF**

**ASMDY--**

**ARRLSTW**

**ARHKAYF**

**ARLPSSW**

**ARQDWYF**

**ARDNYAM**

**ARHGGGN**

**ARRFLYY**

**ARQPNYY**

###### λ5ko Peritoneal Cavity B-2 Amino terminus

**ARHRTY-**

**AKGVLNY**

**ARLDY--**

**AEGFAY-**

**ARSRSFD**

**ASFDY--**

**ARHRFAY**

**ARHPGFA**

**ARHRPYF**

**ALPRAMD**

**ARYDGY-**

**ASWDY--**

**ARHAMDY**

**ARDGTY-**

**ARLAWFA**

**ARFFPGG**

**ARRESYY**

**ARRNWYL**

**ARLNWDG**

**ARRGDYY**

**ARPGSDY**

**ARPGHYY**

**ARPGSSY**

**ARRELGT**

**AGDRGYD**

**ARRGNGY**

**ARQGRYD**

**ARSYYGN**

**ARHDGYF**

**ARHRGRT**

**ARDGPTG**

**ARLGRGF**

**ARWYGNY**

**ARSYRYA**

**ARFGSYG**

**ARPGVYG**

**ARGGIYR**

**ASLYGYH**

**ARVYDYY**

**ARHGYYF**

**ARDGYSF**

**ARGGNWD**

**ARITTAY**

**ARQGITT**

**ARRTMIR**

**ARGTGWY**

**ARHELRL**

**AKDSSGY**

**ARPKGWA**

**ARRENYG**

**ARRYYGS**

**ARGDYGS**

**ARFYYGS**

**ARQGRYD**

**ARGYRYD**

**AIYYGNY**

**ARGGNYG**

**ARQDRYV**

**ARSGYYP**

**ASGNYAM**

**ARLYGNY**

**ARSGYYL**

**ARHRYDA**

**ASNYYFD**

**ARHEAMV**

**AREVRQA**

**AREGSGI**

**ARDHYYG**

**ARRYGSS**

**ARDYGSS**

**ARHYYGS**

**ARPLLSG**

**ARRDRSY**

**ARQGNYD**

**AREDYDY**

**ARHGYDW**

**ARDGAYY**

**ARAVYDY**

**ARSVYGN**

**ARQGAAR**

**ARHWATG**

**TMITTYA**

**TMITTYA**

**ARRLEYF**

**ARKFTTV**

**ARRGWLR**

**ARHSSGY**

**ARDQDYG**

**ARDYYGS**

**ARQTYYY**

**ANYYGSY**

**ARKDYGS**

**ASSSYAM**

**ARGAYYG**

**ARQDGNY**

**ARRGYRS**

**ARELYGN**

**ARELYGN**

**ARHDGYY**

**ARHATVV**

**ARLTTVV**

**ARDAMIT**

**ARHYYGY**

**AGDYYGS**

**ARDYYGS**

**ARDYGSS**

**ARHPHYY**

**ARPYGSS**

**ARFYDGY**

**ARHGYYD**

**SRHYGNY**

**ARSITTV**

**AGLITTV**

**ARLITTV**

**ARRGSYD**

**ARPYGSS**

**ARDRGSS**

**ARDYGSS**

###### λ5ko Peritoneal Cavity B-1a Amino terminus

**ARHFRV-**

**ASGLF--**

**ARHGKFD**

**ARQLY--**

**GFAY---**

**ARQRERG**

**ARHRYFD**

**ARQRPWF**

**ASGYAMD**

**ARHGNYA**

**ARSTMET**

**ARLTGFA**

**ARLYYAM**

**ARHGNYY**

**ARLNWEI**

**ARGGYWY**

**ARHDYYA**

**ARLYYYA**

**ASVYYAM**

**ARHGNFA**

**ARHYFYA**

**ARLMITT**

**ARDIGTF**

**ASLGTGA**

**ARLLWFA**

**ARWLLRA**

**ARLGRYF**

**ARELGRR**

**ARQRGSS**

**ARDLHYY**

**ARHGNAM**

**ARGYYDY**

**ASPWGDY**

**ARGDGYL**

**ARGSYYG**

**ARHWDYA**

**ARHITTV**

**ARHRGIT**

**ARDSMVT**

**ARHMVTT**

**ARSTPSM**

**MVTTFAY**

**ARTIGTT**

**ARQLTGT**

**ARSQLGL**

**ARHGLRR**

**ARPRPFA**

**ARWLLTM**

**ARLGPAW**

**ARDYYGY**

**ARHYYGS**

**ARHYYGS**

**ARDYYGY**

**ARDYYGS**

**ARDGYYA**

**ARGGIYG**

**ARRYGKR**

**ARHDYDW**

**ARQRGNY**

**ARDDGYY**

**ARETNYY**

**ARSMIYY**

**ARHIITT**

**ARPEMVT**

**ARHVVTT**

**ARHRGIY**

**ARSMVTA**

**ARHARLR**

**ARHGRLR**

**ARLGRYY**

**ARQEEDS**

**ARHGNYY**

**ARHYGYD**

**ARDGNYW**

**ARVYDGY**

**ARHANDY**

**ARLHYDY**

**ARDRYDY**

**ARYRYDY**

**ARHRYDY**

**ARHYRYY**

**ARHGNYW**

**ARHGYDW**

**ARHGNYW**

**ARPPYYD**

**ATYYGNY**

**ARGDGYY**

**ARYGYDW**

**ARDGNFA**

**ARHTTAT**

**ARHTTAT**

**ARLTTVV**

**ARVTTVV**

**ARHTMVT**

**ARQTIGT**

**ARSTMIT**

**ARHTRVR**

**ARHYGSS**

**ARHYYGS**

**ARHPPYY**

**ARHYYYG**

**ARHYYGS**

**ARYAFAY**

**ARHERYD**

**ARPSLYR**

**ARDYDYD**

**ARHRGAT**

**ARDGTTP**

**ARHYYGS**

**ARHYRYD**

**ARHGYDD**

**ARHPQTL**

**ARHEGRY**

**ARQCILR**

**ARHYYGS**

**ADGSSWG**

###### λ5ko Peritoneal Cavity B-1b Amino terminus

**ARHKAY-**

**ARRLKAY**

**ARHAY--**

**ARHYFAY**

**ARSPFDY**

**ARPGPFA**

**ARAGFAY**

**ASGVRFA**

**ASKDRFA**

**ARHKAYF**

**ARWPTGA**

**ARGDYFD**

**ARRAIGT**

**ASHPAWF**

**ARPTSPW**

**ARAWFAY**

**ARRGY--**

**AREVAYA**

**ARLYAMD**

**ARFYDGY**

**ARDNYAM**

**ARHGSMI**

**ASSSTMI**

**ARWYYAM**

**ASSYYAM**

**ARHGTPV**

**ARIHYYA**

**ARHDYYA**

**ARSGPYY**

**ARSRGLL**

**ARPNYYG**

**ARGYGNF**

**AAYGNFD**

**ARGNYPY**

**ARSRGYY**

**ARGEVTT**

**ARHPATA**

**ARSGRST**

**ARSIPNL**

**ARSIPNL**

**ARRGTGP**

**ARVLVWY**

**ARRLLWY**

**ARQARGR**

**ARQRGPG**

**ARDWDYD**

**ARGGDYV**

**ARFYEGA**

**ARHDYGF**

**ARGYYDY**

**ARSRDGY**

**ARWEVQF**

**ARGTARA**

**AQTARSW**

**ASTTVVA**

**MMGSAMD**

**ARQRDGL**

**ARLGRDW**

**ARHDYRY**

**AREEGYG**

**ASNGNYV**

**ARGYDAM**

**ARGGIYG**

**ASGNYDF**

**ARHKYGN**

**ASYYGNR**

**ARRGDDY**

**ARSGYRS**

**ARKGISD**

**ARSEGYG**

**ARWDGLY**

**ARRITTA**

**TREAMVN**

**ARHWGVT**

**ARHSMVT**

**ARIDMIR**

**ARGKELL**

**ARSGRGA**

**ARHYGSS**

**ARGSSYV**

**ARYYGLD**

**ARRDYGN**

**ARHYGNR**

**ARDRSYG**

**ARDLGYG**

**ARSGNGY**

**ARSYSMV**

**ARLWLPP**

**ALLRRYA**

**ARDYYGS**

**ARDTALG**

**AANYDGA**

**ARHPLYD**

**ARNYRSH**

**ARHGGGT**

**ARMVKRV**

**ARRIGTT**

**ARLRRRA**

**ARHGGLR**

**ARHGGLR**

**ARGGDHS**

**ARRDDYD**

**ARNYRYD**

**ARATMVT**

**AKWLRRG**

**AREYGSS**

**ARIEGYY**

### Counting from the carboxy terminus end.

##### WT

###### WT Bone Marrow Fraction E Carboxy terminus

**LLFTADV**

**SYYDFDY**

**RHAGRDY**

**DFHYFAY**

**DFHYFAY**

**ITGYFDY**

**ITGAMDY**

**STTVVAP**

**LLRPFAY**

**QNYYEDY**

**RYGGFAY**

**YPYAMDY**

**LRYAMDY**

**LHWYFDV**

**YGYAMDY**

**YGSSFDY**

**YGYAMDY**

**GVRPFDY**

**GLRLFDY**

**GSFSMDY**

**GSSPPDY**

**GSSYFDY**

**TPPLFDY**

**VVAKMDY**

**LRPPFAY**

**GKGYFDY**

**SSYWFDY**

**SSYAMDY**

**SPAWFAY**

**SSYAMDY**

**SSYAMDY**

**PYWYFDV**

**SSYGFAY**

**SSYGGDY**

**VARGFAY**

**TATAMDY**

**TATAMDY**

**INWYFDV**

**SPAWFAY**

**TPWNFDV**

**SYGAMDY**

**SPYAMDY**

**SPGGFAY**

**SLYAMDY**

**SFYAMDY**

**SLLYFGV**

**GYWYFDV**

**GCYAMDY**

**ADWYFDV**

**ATYYFDY**

**PSDYFDY**

**EGLAMDY**

**YPPWFAY**

**WDWYFDV**

**DYYAMDY**

**GYYAMDY**

**YNWYFDV**

**PYWYFDV**

**YLYAMDY**

**RYYAMDY**

**YGDAMDY**

**YVCAMDY**

**RDWYFDV**

**SDYAMDY**

**DYYAMDY**

**PYWYFDV**

**DYYAMDY**

**VGYAMDY**

**VDWYFDV**

**LYWYFDV**

**ELYYLDY**

**SYYAMDY**

**VNWYFDV**

**LYYAMDY**

**ASWGRAY**

**AELGQDY**

**GTGTWAY**

**NWEPFAY**

**NWDGFDY**

**NWAYFDV**

**GRDYFDY**

**AGTGFAY**

**VGRYFDY**

**WEGAMDY**

**GITWFAY**

**GTGAMDY**

**GTGAMDY**

**DLYAMDY**

**IYYAMDY**

**-ASLLLF**

**LDYYLDY**

**YYGPFAY**

**YYGNYPN**

**LDGYYAY**

**HYDPFAY**

**VYDYFAY**

**YYCAMDY**

**PDSAFAY**

**MMVTIDY**

**SMITTAY**

**LPYAMDY**

**DGYYGFY**

**DGFGFAY**

**EGNPFAY**

**YGNSFAY**

**GGNWFAY**

**GGYDGTY**

**NDYGFAY**

**YESAMDH**

**YGNYEAY**

**MITTSDY**

**WSYYFDY**

**WLRDFDY**

**WLHAMDY**

**RLLLFAY**

**WLRRRNY**

**RYDGFAY**

**GYYDFDV**

**GNRGFAY**

**GNPWFAY**

**GNYAVDY**

**GSHAMDY**

**GYDGFAY**

**GYAWFAY**

**GYYYFDY**

**GYPWFAY**

**DYDVFDY**

**RSSWFAY**

**RYDYFDY**

**CTRAMDY**

**ITTGFAY**

**ITYAMDY**

**GTTQFAY**

**LLRAMDY**

**SYHAMDY**

**YSYAMDN**

**YEGAMDY**

**YSWYFDV**

**YLYAMDY**

**NYPWFAY**

**NTGAMDY**

**NPPYFDY**

**NHWYFDV**

**NYEGVDY**

**NPAWFAY**

**NFYAMDY**

**YDNAMDY**

**YLYAMDY**

**YGYAMDY**

**YDEWFAY**

**YDWYFDV**

**YDAWFAY**

**YDAAMDY**

**YADYFDY**

**NYEGVAY**

**NFEYFDV**

**YDYAMDY**

**YDNAMDY**

**YDGAMDY**

**NYYAMDY**

**TGYAMDY**

**TLYAMDY**

**RRDAMDY**

**RGFAMDY**

**GGWYFDV**

**YEAWFAY**

**YNWYFDV**

**YPWYFDV**

**SFPWSAY**

**YSYYFDY**

**YPAWFAY**

**YVSWFAY**

**YVYAMDY**

**YEWYFDV**

**AHYAMDY**

**DGAHFDY**

**DGWYFDV**

**EGYAMDY**

**DEVWFAY**

**DVAWFAY**

**DDYAMDY**

**DVKGSDY**

**PYXAMDY**

**DVGYFDV**

**KGYAMDY**

**RGYAMDY**

**RDGAFDY**

**DPAWFAY**

**VDWYFDV**

**ERHYFDY**

**RYWYFDV**

**DYYAMDY**

**DYYAMDY**

**GGSLMDY**

**DYYAMDY**

**DYYAMDY**

**YYYAMDY**

**FFYAMDY**

**GYYATDY**

**PYYAMDY**

**SPSSMDY**

**QLGLFDY**

**GPAWFAY**

**---QMIH**

**RDLAPRY**

**DPGKLYY**

**---RGYH**

**ARHDRAY**

**-ARHAAY**

**RTPSLAY**

**ARGPWDY**

**RHLPPDY**

**-ARTEDY**

**LVSPSDY**

**QRDGLAY**

**RQRGLDY**

**KRVALDY**

**RDCFFAY**

**-ARHMDY**

**RPESFDY**

**PRAGMDY**

**ARKVFDY**

**NSRGFAY**

**RGEGFAY**

**RGWGFAY**

**GEVGFAY**

**RGFTMDY**

**DEGYFDY**

**-ARYFDY**

**ARRAMDY**

**ARKYFDV**

**TDDWFAY**

**HEGWFAY**

**GGYWFAY**

**GSDATDY**

**LFPWFAY**

**CPYYFDY**

**GGYAMDY**

**YPYAMDY**

**LGAWFAY**

**YGWYFDV**

**DRYAMDY**

**WPYAMDY**

**YSYAMDY**

**GGYYFDY**

**RGYYFDY**

**VPYAMDY**

**NPAWFAY**

**IPYAMDY**

**PLYAMDY**

**GHWYFDV**

**GEYYFDY**

**AYYAMDY**

**DYYAMDY**

**DYYAMDY**

###### WT Peritoneal Cavity B-2 Carboxy terminus

**ARLTTDY**

**GYSPFDY**

**HYYGRDY**

**TTVVFDY**

**LRLRLDY**

**YGSSYDY**

**YGSSPDY**

**YGSSFDY**

**YGSYFDY**

**TVVAFDY**

**RLPYFDV**

**RLRAMDY**

**RSYAMDY**

**GSSDFDY**

**DSSPFDY**

**GSSYFDY**

**GYWYFDV**

**AVANWAY**

**ATEAMDY**

**VVATEDY**

**SSSGFAC**

**SSYAMDY**

**SSWYFDV**

**SSYAMDY**

**SRRAMDY**

**SSGAMDY**

**SPYYFDY**

**SSWYFDV**

**SSCAMDY**

**RNYAMDY**

**YYYAMDY**

**VAEYFDV**

**VANAMDY**

**SHWYFDV**

**SSYYFDY**

**RGNAMDY**

**SYWYFDV**

**SYWYFDV**

**SYVYFDY**

**SYVNFDV**

**ATRYFDY**

**AHWYFDV**

**SFRYFDV**

**YVGAMDY**

**YVPYFDY**

**TRDAMDY**

**TMYYFDY**

**LRWYFDV**

**VGGYFDY**

**GRSYFDY**

**PNWDFDY**

**DSGTFDY**

**GDWYFDY**

**DVKAMDY**

**THYAMDY**

**WRTWFAY**

**ARPDGAY**

**ASYYGNY**

**AYYGTAY**

**ARHDYAY**

**GMIAMDY**

**IEEAMDY**

**RYAWFAY**

**GNYDGDY**

**GNWYFDV**

**GNYAMDY**

**GYDYFDY**

**GNYGLDY**

**DYDGFAY**

**GYYAMDY**

**MEWYFDV**

**TTTVMDY**

**LRPHFDY**

**VLRYFDV**

**LEYAMDY**

**YDDGFDY**

**YDDAMDY**

**YAYAMDY**

**NYYAMDY**

**NYYAMDY**

**NFAWFAY**

**YDAWFAY**

**SLYAMDY**

**TTAWFAY**

**PYYAMDY**

**YGWYFDV**

**YGYAMDY**

**DVGYFDV**

**YVRAMDY**

**GDYAMDY**

**RAYAMDY**

**PYYAMDY**

**DAAWFAY**

**GNYAMDY**

**RQGLFDY**

**SSGYLAY**

**GYDYFDY**

**PFYAMDY**

**VDYAMDY**

**ARDGLDY**

**RGGGFDY**

**PSQAMDY**

**RSQYFDV**

**QRGAMDY**

**SQLYFDY**

**ARPAMDY**

**KSDAMDY**

**HPYYFDY**

**GRWYFDV**

**PVYAMDY**

**RNWYFDV**

**SDWYFDV**

**GYYAMDY**

**YYYAMDY**

###### WT Peritoneal Cavity B-1a Carboxy terminus

**-ARHKSP**

**--ATTGF**

**--ARWDY**

**AGSRPDY**

**--ARDDY**

**-ARGFDY**

**-ARGFDY**

**-ANLFAY**

**ARHLFAY**

**RGTGFAY**

**-ARGFAY**

**-ATGFAY**

**SIRAMDY**

**-AGAMDY**

**--ANWDY**

**ASNYFDY**

**REDAMDY**

**SARYFDV**

**ARLTGAY**

**ARAWFAY**

**EGLRFAY**

**ATWYFDV**

**KQYYFDY**

**ARTGFDY**

**RLTGFAY**

**RYWYFDV**

**RHHGLAY**

**RHYGYDY**

**RSARADY**

**ARHDYDY**

**ARHDYDY**

**SNWDQDY**

**NYYAMDY**

**DYYAMDY**

**PYGNSDY**

**YYGVFDY**

**WRDYFDY**

**SWDYFDY**

**RTTAFDY**

**RHGNYDY**

**ARGNFAY**

**ARLTSAY**

**SGTYFDV**

**RGLRFAY**

**VWYYFDY**

**LGRAMDY**

**YYGSSYY**

**YYGWFAY**

**YDYDFDY**

**YGYAMDY**

**DGYYFDY**

**TTVAFDY**

**HGNAMDY**

**MITPFDY**

**GSYYFDY**

**GTYYFDY**

**YYGYVDY**

**LRLAGSY**

**EVRWFAY**

**GYWYFDV**

**DGYYFDY**

**SGRGFAY**

**YGSRFDY**

**YGSLFDY**

**GNSRMDY**

**RYDAMDY**

**RYDAMDY**

**GNYAMDY**

**GLTWFAY**

**DYDYFDY**

**GNAWFAY**

**GYYAMDY**

**DNNYFDY**

**VRNYFDY**

**TVVAMDY**

**TVVAMDY**

**GNWYFDV**

**GNYAMDY**

**TNYAMDY**

**GSSPLDY**

**GYWYFDV**

**GSSYFAY**

**GSSYFDY**

**GSSPFDY**

**NYWYFDV**

**YNWYFDV**

**YDWYFDV**

**YDYAMDY**

**NYWYFDV**

**YDYAMDY**

**NYDWFAY**

**YYYAMDY**

**NYAWFAY**

**YDYAMDY**

**YDYAMDY**

**NYYAMDY**

**YDGAMDY**

**NYYAMDY**

**NYYAMDY**

**YYYAMDY**

**YPYAMDY**

**ATYYFDY**

**YDYAMDY**

**YDYAMDY**

**VRNYFDY**

**NYWYFDV**

**NYWYFGV**

**NYYAMDY**

**TYYAMDY**

**TTGAMDY**

**RRKDFDY**

**SSYYFDY**

**SSYAMDY**

**SSYYFDY**

**SSYAMDY**

**SSYAMDY**

**PSRAMNY**

**YEDAMDY**

**YYWYFDV**

**LFYAMDY**

**SSYYFDY**

**SSYYFDY**

**SSWYFDV**

**SSWYFDV**

**SSWYFDV**

**SSYAMDY**

**SSYAMDY**

**DYYAMDY**

**TGYYFDY**

**VAGAMDY**

**NYYAMDY**

**SYWYFDV**

**SYWYFDV**

**SYSYFDY**

**SLYAMDY**

**RYWYFDV**

**YYYAMDY**

**YYYAMDY**

**GYYAMDY**

**SYYAMDY**

**ATRAMDY**

**ATRAMDY**

**ATRAMDY**

**IYYAMDY**

**ATRAMDY**

**GVSYFDY**

**TSYYFDY**

**YYYAMDY**

**GYYAMDY**

**YYYAMDY**

**GYYAMDY**

###### WT Peritoneal Cavity B-1b Carboxy terminus

**-ARHRTY**

**AKGVLNY**

**--ARLDY**

**-AEGFAY**

**RSRSFDY**

**--ASFDY**

**ARHRFAY**

**RHPGFAY**

**HRPYFDY**

**LPRAMDY**

**-ARYDGY**

**--ASWDY**

**ARHAMDY**

**-ARDGTY**

**RLAWFAY**

**GGYYFDY**

**SYYAMDY**

**RNWYLDY**

**LNWDGDY**

**DYYAMDY**

**DYYAMDY**

**HYYAMDY**

**GSSYFDY**

**ELGTMDY**

**GYDYFDY**

**GNGYFAY**

**RYDAMDY**

**YYGNFDY**

**HDGYFDY**

**TMITFAY**

**TGTVSSY**

**LGRGFAY**

**YGNYFDY**

**YRYAMDY**

**YGNYFDY**

**YGNPMDY**

**YRYYFDY**

**YGYHFDY**

**DYYAMDY**

**HGYYFDY**

**DGYSFAY**

**WDGYFDV**

**TTAYFDY**

**TTVVADY**

**MIRAMDY**

**TGWYFDV**

**LRLRFDY**

**SGYYFDY**

**KGWAMDY**

**YGHAMDY**

**YGSSFAY**

**YGSTGGY**

**YGSSFDY**

**RYDGFDY**

**RYDWFAY**

**GNYWFAY**

**GNYGFDY**

**RYVAMDY**

**GYYPMDY**

**GNYAMDY**

**GNYYFDY**

**GYYLMDY**

**RYDAMDY**

**SNYYFDY**

**VTAWFAY**

**VRQAMDY**

**GIYAMDY**

**GSTWFAY**

**GSSYFDY**

**GSSYFDY**

**GSSYFDY**

**GSGGFAY**

**SYWYFDV**

**NYDWFAY**

**YDYAMDY**

**YDWYFDV**

**YDGAMDY**

**YPAWFAY**

**NFYAMDY**

**ATYYFDY**

**ATGGFDY**

**TTYAMDY**

**TTYAMDH**

**RLEYFDY**

**VDWYFDV**

**RDYAMDY**

**YYYAMDY**

**SSYWFAY**

**SSWYFDV**

**SYWYFDV**

**SYWYFDV**

**SVYAMDY**

**SSYAMDY**

**LYYAMDY**

**YGGYFDV**

**GYYAMDY**

**YYYAMDY**

**YYYAMDN**

**YGSWFAY**

**VAYYFDY**

**VATPFDY**

**TGNYFDY**

**YVGYFDY**

**SYWYFDV**

**SYWYFDV**

**SYWYFDV**

**SYYAMDY**

**SSYAMDY**

**VGYAMDY**

**DYWYFDV**

**VEYAMDY**

**DYYAMDY**

**ATRAMDY**

**ATRAMDY**

**YDYAMDY**

**GYWYFDV**

**RDYAMDY**

**YYYAMDY**

##### λ5ko

###### λ5ko Bone Marrow Fraction E Carboxy terminus

**NSFHFDY**

**LTTVVDY**

**ILRAMDY**

**RHEVADY**

**YDYAMDY**

**TATWFAY**

**GSSYFAY**

**GSSWFAY**

**LYWYFDV**

**VDAWFAY**

**SYVNFGV**

**SGYAMDY**

**SYGYFDV**

**SYYAMDY**

**SYWYFDV**

**GGRDFAY**

**HYNAMDY**

**HYYAMDY**

**HYYAMDY**

**HYYAMDY**

**RHLRDGY**

**RHPTRDY**

**-ARDGNY**

**EGAMIGY**

**ARQVITT**

**HLLYFDY**

**YYGNLDY**

**LDDYAYY**

**YDGYCYY**

**GMVTTDY**

**TMITFAY**

**YGNYGAY**

**GGNYPSY**

**VRDYLDY**

**MVTTFDY**

**RLLAMDY**

**DYAWFAY**

**DYGAMDY**

**GKRAMDY**

**LYDAMDY**

**GYDVIAY**

**GTAWFAY**

**VTEGFAY**

**ITSAMDY**

**ITTAMDY**

**VRDSMDY**

**SGYAMDY**

**LLLWFAY**

**HDGGFAY**

**YDGAMDY**

**NYGAMDY**

**YPYAMDY**

**THWYFDV**

**TTAWFAY**

**RRDWFAY**

**RRWYFDV**

**RPPAMDY**

**YYYAMDY**

**YYYAMDY**

**AYYAMDY**

**RAAWFAY**

**YYYAMDY**

**GYYAMDY**

**GYYAMDY**

**GCYAMDY**

**GYYAMDY**

**RARAMDY**

**LLYAMDY**

**YYYAMDY**

**ARPIPPG**

**-ATLTPY**

**VFIGFDY**

**--ASMDY**

**STWGFAY**

**HKAYFDY**

**PSSWFAY**

**QDWYFDV**

**DNYAMDY**

**GNWYFDV**

**LYYAMDY**

**NYYAMDY**

###### λ5ko Peritoneal Cavity B-2 Carboxy terminus

**-ARHRTY**

**AKGVLNY**

**--ARLDY**

**-AEGFAY**

**RSRSFDY**

**--ASFDY**

**ARHRFAY**

**RHPGFAY**

**HRPYFDY**

**LPRAMDY**

**-ARYDGY**

**--ASWDY**

**ARHAMDY**

**-ARDGTY**

**RLAWFAY**

**GGYYFDY**

**SYYAMDY**

**RNWYLDY**

**LNWDGDY**

**DYYAMDY**

**DYYAMDY**

**HYYAMDY**

**GSSYFDY**

**ELGTMDY**

**GYDYFDY**

**GNGYFAY**

**RYDAMDY**

**YYGNFDY**

**HDGYFDY**

**TMITFAY**

**TGTVSSY**

**LGRGFAY**

**YGNYFDY**

**YRYAMDY**

**YGNYFDY**

**YGNPMDY**

**YRYYFDY**

**YGYHFDY**

**DYYAMDY**

**HGYYFDY**

**DGYSFAY**

**WDGYFDV**

**TTAYFDY**

**TTVVADY**

**MIRAMDY**

**TGWYFDV**

**LRLRFDY**

**SGYYFDY**

**KGWAMDY**

**YGHAMDY**

**YGSSFAY**

**YGSTGGY**

**YGSSFDY**

**RYDGFDY**

**RYDWFAY**

**GNYWFAY**

**GNYGFDY**

**RYVAMDY**

**GYYPMDY**

**GNYAMDY**

**GNYYFDY**

**GYYLMDY**

**RYDAMDY**

**SNYYFDY**

**VTAWFAY**

**VRQAMDY**

**GIYAMDY**

**GSTWFAY**

**GSSYFDY**

**GSSYFDY**

**GSSYFDY**

**GSGGFAY**

**SYWYFDV**

**NYDWFAY**

**YDYAMDY**

**YDWYFDV**

**YDGAMDY**

**YPAWFAY**

**NFYAMDY**

**ATYYFDY**

**ATGGFDY**

**TTYAMDY**

**TTYAMDH**

**RLEYFDY**

**VDWYFDV**

**RDYAMDY**

**YYYAMDY**

**SSYWFAY**

**SSWYFDV**

**SYWYFDV**

**SYWYFDV**

**SVYAMDY**

**SSYAMDY**

**LYYAMDY**

**YGGYFDV**

**GYYAMDY**

**YYYAMDY**

**YYYAMDN**

**YGSWFAY**

**VAYYFDY**

**VATPFDY**

**TGNYFDY**

**YVGYFDY**

**SYWYFDV**

**SYWYFDV**

**SYWYFDV**

**SYYAMDY**

**SSYAMDY**

**VGYAMDY**

**DYWYFDV**

**VEYAMDY**

**DYYAMDY**

**ATRAMDY**

**ATRAMDY**

**YDYAMDY**

**GYWYFDV**

**RDYAMDY**

**YYYAMDY**

###### λ5ko Peritoneal Cavity B-1a Carboxy terminus

**-ARHFRV**

**--ASGLF**

**RHGKFDY**

**--ARQLY**

**---GFAY**

**RERGFAY**

**RHRYFDY**

**QRPWFAY**

**SGYAMDY**

**GNYAMDY**

**RSTMETY**

**RLTGFAY**

**LYYAMDY**

**ARHGNYY**

**LNWEIFY**

**GYWYFDV**

**DYYAMDY**

**YYYAMDY**

**VYYAMDY**

**RHGNFAY**

**YFYAMDY**

**LMITTPY**

**DIGTFDY**

**SLGTGAY**

**RLLWFAY**

**RWLLRAY**

**LGRYFDV**

**LGRRFAY**

**SSGYEAY**

**YYGYFDY**

**HGNAMDY**

**YDYDFDY**

**GDYAFAY**

**DGYLLDY**

**YGNYVAY**

**WDYAMDY**

**TTVVVDY**

**TTVVFAY**

**MVTTMDY**

**MVTTFAY**

**MVIYFDY**

**MVTTFAY**

**IGTTFAY**

**GTWYFDV**

**LGLRFAY**

**GLRRFAY**

**RPRPFAY**

**WLLTMDY**

**GPAWFAY**

**YGYAMDY**

**YGSAMDY**

**YGSSFDY**

**YGYWFAY**

**YGSSFDY**

**GYYAMDY**

**GKRAMDY**

**GKRAMDY**

**DYDWFAY**

**GNYAFAY**

**GYYAMDY**

**FLYAMDY**

**DYSWFAY**

**TVVGFDY**

**VTAWFAY**

**VTTAMDY**

**GIYAMDY**

**VTAWFAY**

**LRQGFAY**

**LRRAFAY**

**RYYAMDY**

**GYYAMDY**

**NYYAMDY**

**YDYAMDY**

**NYWYFDV**

**YGAWFAY**

**YDYAMDY**

**YDYAMDY**

**YDYAMDY**

**YDYAMDY**

**YDYAMDY**

**YYYAMDY**

**NYWYFDV**

**YDWYFDV**

**NYWYFDV**

**YLAWFAY**

**NYYAMDY**

**YYYAMDY**

**YDWCFDV**

**NFAWFAY**

**ATYYFDY**

**ATAWFAY**

**VVAWFAY**

**VVATFAY**

**TTYYFDY**

**TTRAMDY**

**TTYYFDY**

**RYYAMDY**

**SSYYFDY**

**SSYYFDY**

**SSYAMDY**

**SSYAMDY**

**SSYAMDY**

**DGGYFDY**

**DEAWFAY**

**DGAWFAY**

**DLAWFAY**

**TGRVFAY**

**PKNAMDY**

**SYYAMDY**

**DYYAMDY**

**DYWYFDV**

**GGAWFAY**

**VFYAMDY**

**GYYAMDY**

**YYYAMDY**

**WGYAMDY**

###### λ5ko Peritoneal Cavity B-1b Carboxy terminus

**-ARHKAY**

**ARRLKAY**

**--ARHAY**

**ARHYFAY**

**ARSPFDY**

**RPGPFAY**

**ARAGFAY**

**SGVRFAY**

**SKDRFAY**

**HKAYFDY**

**PTGAMDY**

**RGDYFDY**

**ARRAIGT**

**HPAWFAY**

**SPWYFDV**

**ARAWFAY**

**--ARRGY**

**VAYAMDY**

**RLYAMDY**

**RFYDGYY**

**DNYAMDY**

**GSMIFAY**

**SSTMITY**

**WYYAMDY**

**SYYAMDY**

**RYYAMDY**

**HYYAMDY**

**DYYAMDY**

**YYYAMDY**

**LLAWFAY**

**NYYGYAY**

**GYGNFDY**

**AYGNFDY**

**NYPYFDV**

**YYGSSDY**

**VTTVVDY**

**RHPATAY**

**TMIPFAY**

**LMVNFDY**

**LMVNFGY**

**TGPWFAY**

**VWYLFDY**

**LWYYFDY**

**RGRGFAY**

**RGPGFAY**

**YDYWFAY**

**GDYVFAY**

**YEGAFDD**

**HDYGFAY**

**YDYDFDY**

**DGYYFDY**

**WEVQFAY**

**ARARFAY**

**ARSWFAY**

**TTVVATD**

**MGSAMDY**

**GLRGFAY**

**GRDWFAY**

**RYAWFAY**

**GYPWFAY**

**GNYVFAY**

**GYDAMDY**

**GKRAMDY**

**GNYDFDY**

**GNYEFAY**

**GNRGFAY**

**DYEFFDY**

**RSPWFAY**

**DYPWFAY**

**GNYVFDY**

**GLYAMDY**

**TAVYFDY**

**VNYAMDY**

**VTPWFAY**

**VTSWFAY**

**IRGAMDY**

**ELLLFAY**

**GRGAMDY**

**GSSYFDY**

**GSSYVAY**

**GLDWFAY**

**NFWYFDV**

**NRYAMDY**

**NLAWFAY**

**NLAWFAY**

**YALYFDY**

**TSYYFDY**

**PPYAMDY**

**RRYAMDY**

**SYWYFDV**

**YPAWFAY**

**DGAWFAY**

**YVGAMDY**

**HYYAMDY**

**TDDGGAY**

**RVAWFAY**

**TRVAMDY**

**RRAWFAY**

**RSYAMDY**

**RSYAMGY**

**RSYYFDY**

**EKAWFAY**

**GGYYFDY**

**GYWYFDV**

**GYYAMDY**

**RGWYFDV**

**YYYAMDY**

## DFL-Containing CDR-H3

### Raw Sequences

##### WT

###### WT DFL Bone Marrow Fraction E

| TRLLFTADV |
| --- |
| ASYYDFDY |
| ARHAGRDY |
| TRDFHYFAY |
| TRDFHYFAY |
| ARRGITGYFDY |
| ARPSITGAMDY |
| TSTTVVAP |
| ARHEGLLRPFAY |
| ARQNYYEDY |
| ASHRYGGFAY |
| ARQENYPYAMDY |
| ARSLRYAMDY |
| ARALLHWYFDV |
| ARDHRYGYAMDY |
| ARHYYGSSFDY |
| ARHALYYGYAMDY |
| ARGGVRPFDY |
| ARQGLRLFDY |
| ARHDYYGSFSMDY |
| ARHAYYYGSSPPDY |
| ARRVYYYGSSYFDY |
| ARTPLITKTPPLFDY |
| AREGFTTVVAKMDY |
| ATLLRPPFAY |
| ARQEGGKGYFDY |
| ARHYYGSSYWFDY |
| ARHDYYGSSYAMDY |
| ARDYYGSPAWFAY |
| AGDYYGSSYAMDY |
| ARYHGSSYAMDY |
| ARFYYYGPYWYFDV |
| ARDCSSYGFAY |
| ARLLYGSSYGGDY |
| ARPSKFITTVVARGFAY |
| ARHHYSTATAMDY |
| AREATATAMDY |
| ARHHYYGSINWYFDV |
| ARQSYYYGSSPAWFAY |
| ARHGPIYYGSTPWNFDV |
| ARDPYGSSYGAMDY |
| ARHGSSPYAMDY |
| ANYYGSSPGGFAY |
| ARSHYYGSSLYAMDY |
| ARTGSSFYAMDY |
| ARGGYGSSLLYFGV |
| ARGYYGYWYFDV |
| ARGRVHYYGCYAMDY |
| AREFITTVVADWYFDV |
| ARGTTATYYFDY |
| ARVLRPSDYFDY |
| ARHSLLREGLAMDY |
| ARDPNYYGSSYPPWFAY |
| ARQGHYYGSSWDWYFDV |
| AKPYYGSRDYYAMDY |
| ARDYYGSRGYYAMDY |
| ARANYYGSSYNWYFDV |
| TRSYYGSSPYWYFDV |
| ARGLYYGSSYLYAMDY |
| ASPHYYGRYYAMDY |
| ARHYYGYGDAMDY |
| TLHYYGYVCAMDY |
| ARDRITTARDWYFDV |
| AREALRSSDYAMDY |
| ARREDYYGSQRDYYAMDY |
| ARSRKIDYYGSRAPYWYFDV |
| ASGNYYGSSYDYYAMDY |
| ARDRRYGSSYVGYAMDY |
| ARDPYYGSSYVDWYFDV |
| ARHPHYYGSSSLYWYFDV |
| ARSPYGSSPELYYLDY |
| ARDIYYYGSSSSYYAMDY |
| AREGSYYGYVNWYFDV |
| ARETSYYYGSSYSLYYAMDY |

###### WT DFL Peritoneal Cavity B-2

| ARLTTDY |
| --- |
| ARSLGGYSPFDY |
| ARHYYGRDY |
| ARITTVVFDY |
| ASLLLRLRLDY |
| AREANYGSSYDY |
| ARSPYYGSSPDY |
| ARYYYGSSFDY |
| ARLYGSYFDY |
| ARDTTVVAFDY |
| AREGLRLPYFDV |
| AREGLRLRAMDY |
| ARELLRSYAMDY |
| ARHGDYYGSSDFDY |
| ARDSSPFDY |
| ARHYGSSYFDY |
| ARHYYGYWYFDV |
| AVANWAY |
| ARVTTATEAMDY |
| ARHTVVATEDY |
| ARHEGSSSGFAC |
| ARRDYYYYGSSYAMDY |
| ARDYYGSSWYFDV |
| ARDHYYGSSYAMDY |
| ARVYYYGSRRAMDY |
| ARRGSSGAMDY |
| ARCGYYGSPYYFDY |
| ARFYYGSSWYFDV |
| AYYYGSSCAMDY |
| ARSTARNYAMDY |
| ARGRTTAYYYAMDY |
| ARHPYITTVVAEYFDV |
| ARLNSLITTVVANAMDY |
| ASDYGSSHWYFDV |
| ARDGTYYYGSSSYYFDY |
| ASPAYYYGSRGNAMDY |
| ARPGSSYWYFDV |
| ASYGSSYWYFDV |
| ARDYGSSYVYFDY |
| ARSGYGSSYVNFDV |
| ARRGTVVATRYFDY |
| ARGTTVVAHWYFDV |
| ARQGIRRNHSRSFRYFDV |
| ARRKPYGSSYVGAMDY |
| ARNYGSSYVPYFDY |
| ARQGITTVVATRDAMDY |
| ARGITTVVATMYYFDY |
| ARQGEGSSYLRWYFDV |
| ARHWNYGSSYVGGYFDY |
| ARMVATSGRSYFDY |

###### WT DFL Peritoneal Cavity B-1a

| ARHRTTAFDY |
| --- |
| AIYYYGSSYY |
| ARPLLHYYGWFAY |
| ARRLFTTVAFDY |
| ARHYYGYVDY |
| ARSELLRLAGSY |
| ARQDGYYFDY |
| ARSGRGFAY |
| ARGYGSRFDY |
| ARQSYGSLFDY |
| ARQGGWGIITVRNYFDY |
| ARWDTTVVAMDY |
| ARHTTVVAMDY |
| ARHDGSSPLDY |
| ARHYYGYWYFDV |
| ARPNYYGSSYFAY |
| ARYYGSSYFDY |
| ARGAGSYGSSPFDY |
| ARAVRNYFDY |
| ARHYYGSSYYFDY |
| ARRYYYGSSYAMDY |
| ARYYYGSSYYFDY |
| ARFYYGSSYAMDY |
| ARDFIYYYGSSYAMDY |
| ARHYYGSSYYFDY |
| ARHYYGSSYYFDY |
| TRDYYGSSWYFDV |
| ARDYYGSSWYFDV |
| ARHYYGSSWYFDV |
| ARHYYGSSYAMDY |
| ARHYGSSYAMDY |
| AMTTATGYYFDY |
| AVITTVVAGAMDY |
| ARPYYYGSSYWYFDV |
| ARQAYYGSSYWYFDV |
| ARGYGSSYSYFDY |
| ARGYYGSSLYAMDY |
| ARHYYGSSYYAMDY |
| ARRITTVVATRAMDY |
| ARRITTVVATRAMDY |
| ARRITTVVATRAMDY |
| ARHGTTVVIYYAMDY |
| ARLITTVVATRAMDY |
| ARDRGSRTSYYFDY |
| ARLVYYYGSSYYYAMDY |
| ARHYYGSSYYYAMDY |

###### WT DFL Peritoneal Cavity B-1b

| ARITTAYFDY |
| --- |
| ARQGITTVVADY |
| ARHELRLRFDY |
| ARRENYGHAMDY |
| ARRYYGSSFAY |
| ARGDYGSTGGY |
| ARFYYGSSFDY |
| ARDHYYGSTWFAY |
| ARRYGSSYFDY |
| ARDYGSSYFDY |
| ARHYYGSSYFDY |
| ARPLLSGSGGFAY |
| ARRLEYFDY |
| ARKFTTVDWYFDV |
| ARDQDYGSSYWFAY |
| ARDYYGSSWYFDV |
| ARQTYYYGSYWYFDV |
| ANYYGSYWYFDV |
| ARKDYGSVYAMDY |
| ASSSYAMDY |
| ARGAYYGLYYAMDY |
| ARHATVVAYYFDY |
| ARLTTVVATPFDY |
| ARHYYGYVGYFDY |
| AGDYYGSSYWYFDV |
| ARDYYGSSYWYFDV |
| ARDYGSSYWYFDV |
| ARHPHYYGSSYYAMDY |
| ARPYGSSSYAMDY |
| ARSITTVVDYYAMDY |
| AGLITTVVATRAMDY |
| ARLITTVVATRAMDY |
| ARRGSYDYAMDY |
| ARPYGSSGYWYFDV |
| ARDRGSSQRDYAMDY |
| ARDYGSSYVYYYAMDY |

##### λ5ko

###### λ5ko DFL Bone Marrow Fraction E

| AREEENSFHFDY |
| --- |
| ARLTTVVDY |
| ARHEILRAMDY |
| ARHEVADY |
| ARVPDYYDYAMDY |
| AHITTATWFAY |
| ARHDYYGSSYFAY |
| ARSFYYGSSWFAY |
| ARQRHSLLRLYWYFDV |
| ARAITTVVDAWFAY |
| AREDYGSSYVNFGV |
| ARDPCYGSSGYAMDY |
| ARDGTTAVSYGYFDV |
| ARHNRGSSSYYAMDY |
| RGSSPSYWYFDV |

###### λ5ko DFL Peritoneal Cavity B-2

| ARSPLYYYGFDY |
| --- |
| ARRLRSMDY |
| ARERGSLLRTQAY |
| VYGSEAY |
| ARYYGYWFAY |
| ARRITTAVYFDY |
| ARREPGATVVSMDY |
| ASSNSLLRPSWFAY |
| AREATGRGDY |
| ARRDTTVVDAMDY |
| AITTATYWFAY |
| ARAPGSRDAMDY |
| ARGYDYGSLAWFAY |
| ARHPYGYDYAMDY |
| ARHYYYGSSYAMDY |
| ARGITTVVATNFDY |
| ARSPFITTVVGAWFAY |
| ARASLLRLPTLYFDY |
| ARGGDHSRSYYFDY |
| ARSFYYYGSSYWYFDV |
| ARLGAVVATDYAMDY |
| ARQGTVVEGYYYAMDY |
| ARRXXLPPLWPMDY |
| ARRATTVVATFDYYAMDY |

###### λ5ko DFL Peritoneal Cavity B-1a

| ASVYYAMDY |
| --- |
| ARLLWFAY |
| ARDLHYYGYFDY |
| ARHITTVVVDY |
| ARHRGITTVVFAY |
| ARDYYGYAMDY |
| ARHYYGSAMDY |
| ARHYYGSSFDY |
| ARDYYGYWFAY |
| ARDYYGSSFDY |
| ARHIITTVVGFDY |
| ARHTTATYYFDY |
| ARHTTATAWFAY |
| ARLTTVVAWFAY |
| ARVTTVVATFAY |
| ARHYGSSYYFDY |
| ARHYYGSSYYFDY |
| ARHPPYYYGSSYAMDY |
| ARHYYYGSSYAMDY |
| ARHYYGSSYAMDY |
| ARHYYGSSYYAMDY |
| ARQCILRLQGYYAMDY |
| ARHYYGSSYYYAMDY |
| ADGSSWGYAMDY |

###### λ5ko DFL Peritoneal Cavity B-1b

| ARSRGLLAWFAY |
| --- |
| ARPNYYGYAY |
| ARGEVTTVVDY |
| ARHPATAY |
| ASTTVVATD |
| ARRITTAVYFDY |
| ARHYGSSYFDY |
| ARGSSYVAY |
| ARYYGLDWFAY |
| ARDYYGSYWYFDV |
| ARGGDHSRSYYFDY |
| AREYGSSRGWYFDV |
| ARIEGYYYGSSLYYYAMDY |

### Counting from the amino terminus end.

###### WT DFL Bone Marrow Fraction E Amino terminus

**TRLLFTA**

**ASYYDFD**

**ARHAGRD**

**TRDFHYF**

**TRDFHYF**

**ARRGITG**

**ARPSITG**

**TSTTVVA**

**ARHEGLL**

**ARQNYYE**

**ASHRYGG**

**ARQENYP**

**ARSLRYA**

**ARALLHW**

**ARDHRYG**

**ARHYYGS**

**ARHALYY**

**ARGGVRP**

**ARQGLRL**

**ARHDYYG**

**ARHAYYY**

**ARRVYYY**

**ARTPLIT**

**AREGFTT**

**ATLLRPP**

**ARQEGGK**

**ARHYYGS**

**ARHDYYG**

**ARDYYGS**

**AGDYYGS**

**ARYHGSS**

**ARFYYYG**

**ARDCSSY**

**ARLLYGS**

**ARPSKFI**

**ARHHYST**

**AREATAT**

**ARHHYYG**

**ARQSYYY**

**ARHGPIY**

**ARDPYGS**

**ARHGSSP**

**ANYYGSS**

**ARSHYYG**

**ARTGSSF**

**ARGGYGS**

**ARGYYGY**

**ARGRVHY**

**AREFITT**

**ARGTTAT**

**ARVLRPS**

**ARHSLLR**

**ARDPNYY**

**ARQGHYY**

**AKPYYGS**

**ARDYYGS**

**ARANYYG**

**TRSYYGS**

**ARGLYYG**

**ASPHYYG**

**ARHYYGY**

**TLHYYGY**

**ARDRITT**

**AREALRS**

**ARREDYY**

**ARSRKID**

**ASGNYYG**

**ARDRRYG**

**ARDPYYG**

**ARHPHYY**

**ARSPYGS**

**ARDIYYY**

**AREGSYY**

**ARETSYY**

###### WT DFL Peritoneal Cavity B-2 Amino terminus

**ARLTTDY**

**ARSLGGY**

**ARHYYGR**

**ARITTVV**

**ASLLLRL**

**AREANYG**

**ARSPYYG**

**ARYYYGS**

**ARLYGSY**

**ARDTTVV**

**AREGLRL**

**AREGLRL**

**ARELLRS**

**ARHGDYY**

**ARDSSPF**

**ARHYGSS**

**ARHYYGY**

**AVANWAY**

**ARVTTAT**

**ARHTVVA**

**ARHEGSS**

**ARRDYYY**

**ARDYYGS**

**ARDHYYG**

**ARVYYYG**

**ARRGSSG**

**ARCGYYG**

**ARFYYGS**

**AYYYGSS**

**ARSTARN**

**ARGRTTA**

**ARHPYIT**

**ARLNSLI**

**ASDYGSS**

**ARDGTYY**

**ASPAYYY**

**ARPGSSY**

**ASYGSSY**

**ARDYGSS**

**ARSGYGS**

**ARRGTVV**

**ARGTTVV**

**ARQGIRR**

**ARRKPYG**

**ARNYGSS**

**ARQGITT**

**ARGITTV**

**ARQGEGS**

**ARHWNYG**

**ARMVATS**

###### WT DFL Peritoneal Cavity B-1a Amino terminus

**ARHRTTA**

**AIYYYGS**

**ARPLLHY**

**ARRLFTT**

**ARHYYGY**

**ARSELLR**

**ARQDGYY**

**ARSGRGF**

**ARGYGSR**

**ARQSYGS**

**ARQGGWG**

**ARWDTTV**

**ARHTTVV**

**ARHDGSS**

**ARHYYGY**

**ARPNYYG**

**ARYYGSS**

**ARGAGSY**

**ARAVRNY**

**ARHYYGS**

**ARRYYYG**

**ARYYYGS**

**ARFYYGS**

**ARDFIYY**

**ARHYYGS**

**ARHYYGS**

**TRDYYGS**

**ARDYYGS**

**ARHYYGS**

**ARHYYGS**

**ARHYGSS**

**AMTTATG**

**AVITTVV**

**ARPYYYG**

**ARQAYYG**

**ARGYGSS**

**ARGYYGS**

**ARHYYGS**

**ARRITTV**

**ARRITTV**

**ARRITTV**

**ARHGTTV**

**ARLITTV**

**ARDRGSR**

**ARLVYYY**

**ARHYYGS**

###### WT DFL Peritoneal Cavity B-1b Amino terminus

**ARITTAY**

**ARQGITT**

**ARHELRL**

**ARRENYG**

**ARRYYGS**

**ARGDYGS**

**ARFYYGS**

**ARDHYYG**

**ARRYGSS**

**ARDYGSS**

**ARHYYGS**

**ARPLLSG**

**ARRLEYF**

**ARKFTTV**

**ARDQDYG**

**ARDYYGS**

**ARQTYYY**

**ANYYGSY**

**ARKDYGS**

**ASSSYAM**

**ARGAYYG**

**ARHATVV**

**ARLTTVV**

**ARHYYGY**

**AGDYYGS**

**ARDYYGS**

**ARDYGSS**

**ARHPHYY**

**ARPYGSS**

**ARSITTV**

**AGLITTV**

**ARLITTV**

**ARRGSYD**

**ARPYGSS**

**ARDRGSS**

**ARDYGSS**

##### λ5ko

###### λ5ko DFL Bone Marrow Fraction E Amino terminus

**AREEENS**

**ARLTTVV**

**ARHEILR**

**ARHEVAD**

**ARVPDYY**

**AHITTAT**

**ARHDYYG**

**ARSFYYG**

**ARQRHSL**

**ARAITTV**

**AREDYGS**

**ARDPCYG**

**ARDGTTA**

**ARHNRGS**

**RGSSPSY**

###### λ5ko DFL Peritoneal Cavity B-2 Amino terminus

**ARSPLYY**

**ARRLRSM**

**ARERGSL**

**VYGSEAY**

**ARYYGYW**

**ARRITTA**

**ARREPGA**

**ASSNSLL**

**AREATGR**

**ARRDTTV**

**AITTATY**

**ARAPGSR**

**ARGYDYG**

**ARHPYGY**

**ARHYYYG**

**ARGITTV**

**ARSPFIT**

**ARASLLR**

**ARGGDHS**

**ARSFYYY**

**ARLGAVV**

**ARQGTVV**

**ARRXXLP**

**ARRATTV**

###### λ5ko DFL Peritoneal Cavity B-1a Amino terminus

**ASVYYAM**

**ARLLWFA**

**ARDLHYY**

**ARHITTV**

**ARHRGIT**

**ARDYYGY**

**ARHYYGS**

**ARHYYGS**

**ARDYYGY**

**ARDYYGS**

**ARHIITT**

**ARHTTAT**

**ARHTTAT**

**ARLTTVV**

**ARVTTVV**

**ARHYGSS**

**ARHYYGS**

**ARHPPYY**

**ARHYYYG**

**ARHYYGS**

**ARHYYGS**

**ARQCILR**

**ARHYYGS**

**ADGSSWG**

###### λ5ko DFL Peritoneal Cavity B-1b Amino terminus

**ARSRGLL**

**ARPNYYG**

**ARGEVTT**

**ARHPATA**

**ASTTVVA**

**ARRITTA**

**ARHYGSS**

**ARGSSYV**

**ARYYGLD**

**ARDYYGS**

**ARGGDHS**

**AREYGSS**

**ARIEGYY**

### Counting from the carboxy terminus end.

###### WT DFL Bone Marrow Fraction E Carboxy terminus

**LLFTADV**

**SYYDFDY**

**RHAGRDY**

**DFHYFAY**

**DFHYFAY**

**ITGYFDY**

**ITGAMDY**

**STTVVAP**

**LLRPFAY**

**QNYYEDY**

**RYGGFAY**

**YPYAMDY**

**LRYAMDY**

**LHWYFDV**

**YGYAMDY**

**YGSSFDY**

**YGYAMDY**

**GVRPFDY**

**GLRLFDY**

**GSFSMDY**

**GSSPPDY**

**GSSYFDY**

**TPPLFDY**

**VVAKMDY**

**LRPPFAY**

**GKGYFDY**

**SSYWFDY**

**SSYAMDY**

**SPAWFAY**

**SSYAMDY**

**SSYAMDY**

**PYWYFDV**

**SSYGFAY**

**SSYGGDY**

**VARGFAY**

**TATAMDY**

**TATAMDY**

**INWYFDV**

**SPAWFAY**

**TPWNFDV**

**SYGAMDY**

**SPYAMDY**

**SPGGFAY**

**SLYAMDY**

**SFYAMDY**

**SLLYFGV**

**GYWYFDV**

**GCYAMDY**

**ADWYFDV**

**ATYYFDY**

**PSDYFDY**

**EGLAMDY**

**YPPWFAY**

**WDWYFDV**

**DYYAMDY**

**GYYAMDY**

**YNWYFDV**

**PYWYFDV**

**YLYAMDY**

**RYYAMDY**

**YGDAMDY**

**YVCAMDY**

**RDWYFDV**

**SDYAMDY**

**DYYAMDY**

**PYWYFDV**

**DYYAMDY**

**VGYAMDY**

**VDWYFDV**

**LYWYFDV**

**ELYYLDY**

**SYYAMDY**

**VNWYFDV**

**LYYAMDY**

###### WT DFL Peritoneal Cavity B-2 Carboxy terminus

**ARLTTDY**

**GYSPFDY**

**HYYGRDY**

**TTVVFDY**

**LRLRLDY**

**YGSSYDY**

**YGSSPDY**

**YGSSFDY**

**YGSYFDY**

**TVVAFDY**

**RLPYFDV**

**RLRAMDY**

**RSYAMDY**

**GSSDFDY**

**DSSPFDY**

**GSSYFDY**

**GYWYFDV**

**AVANWAY**

**ATEAMDY**

**VVATEDY**

**SSSGFAC**

**SSYAMDY**

**SSWYFDV**

**SSYAMDY**

**SRRAMDY**

**SSGAMDY**

**SPYYFDY**

**SSWYFDV**

**SSCAMDY**

**RNYAMDY**

**YYYAMDY**

**VAEYFDV**

**VANAMDY**

**SHWYFDV**

**SSYYFDY**

**RGNAMDY**

**SYWYFDV**

**SYWYFDV**

**SYVYFDY**

**SYVNFDV**

**ATRYFDY**

**AHWYFDV**

**SFRYFDV**

**YVGAMDY**

**YVPYFDY**

**TRDAMDY**

**TMYYFDY**

**LRWYFDV**

**VGGYFDY**

**GRSYFDY**

###### WT DFL Peritoneal Cavity B-1a Carboxy terminus

**RTTAFDY**

**YYGSSYY**

**YYGWFAY**

**TTVAFDY**

**YYGYVDY**

**LRLAGSY**

**DGYYFDY**

**SGRGFAY**

**YGSRFDY**

**YGSLFDY**

**VRNYFDY**

**TVVAMDY**

**TVVAMDY**

**GSSPLDY**

**GYWYFDV**

**GSSYFAY**

**GSSYFDY**

**GSSPFDY**

**VRNYFDY**

**SSYYFDY**

**SSYAMDY**

**SSYYFDY**

**SSYAMDY**

**SSYAMDY**

**SSYYFDY**

**SSYYFDY**

**SSWYFDV**

**SSWYFDV**

**SSWYFDV**

**SSYAMDY**

**SSYAMDY**

**TGYYFDY**

**VAGAMDY**

**SYWYFDV**

**SYWYFDV**

**SYSYFDY**

**SLYAMDY**

**SYYAMDY**

**ATRAMDY**

**ATRAMDY**

**ATRAMDY**

**IYYAMDY**

**ATRAMDY**

**TSYYFDY**

**YYYAMDY**

**YYYAMDY**

###### WT DFL Peritoneal Cavity B-1b Carboxy terminus

**TTAYFDY**

**TTVVADY**

**LRLRFDY**

**YGHAMDY**

**YGSSFAY**

**YGSTGGY**

**YGSSFDY**

**GSTWFAY**

**GSSYFDY**

**GSSYFDY**

**GSSYFDY**

**GSGGFAY**

**RLEYFDY**

**VDWYFDV**

**SSYWFAY**

**SSWYFDV**

**SYWYFDV**

**SYWYFDV**

**SVYAMDY**

**SSYAMDY**

**LYYAMDY**

**VAYYFDY**

**VATPFDY**

**YVGYFDY**

**SYWYFDV**

**SYWYFDV**

**SYWYFDV**

**SYYAMDY**

**SSYAMDY**

**DYYAMDY**

**ATRAMDY**

**ATRAMDY**

**YDYAMDY**

**GYWYFDV**

**RDYAMDY**

**YYYAMDY**

##### λ5ko

###### λ5ko DFL Bone Marrow Fraction E Carboxy terminus

**NSFHFDY**

**LTTVVDY**

**ILRAMDY**

**RHEVADY**

**YDYAMDY**

**TATWFAY**

**GSSYFAY**

**GSSWFAY**

**LYWYFDV**

**VDAWFAY**

**SYVNFGV**

**SGYAMDY**

**SYGYFDV**

**SYYAMDY**

**SYWYFDV**

###### λ5ko DFL Peritoneal Cavity B-2 Carboxy terminus

**YYYGFDY**

**RLRSMDY**

**LLRTQAY**

**VYGSEAY**

**YGYWFAY**

**TAVYFDY**

**TVVSMDY**

**RPSWFAY**

**ATGRGDY**

**VVDAMDY**

**ATYWFAY**

**SRDAMDY**

**SLAWFAY**

**YDYAMDY**

**SSYAMDY**

**VATNFDY**

**VGAWFAY**

**PTLYFDY**

**RSYYFDY**

**SYWYFDV**

**TDYAMDY**

**YYYAMDY**

**PLWPMDY**

**DYYAMDY**

###### λ5ko DFL Peritoneal Cavity B-1a Carboxy terminus

**VYYAMDY**

**RLLWFAY**

**YYGYFDY**

**TTVVVDY**

**TTVVFAY**

**YGYAMDY**

**YGSAMDY**

**YGSSFDY**

**YGYWFAY**

**YGSSFDY**

**TVVGFDY**

**ATYYFDY**

**ATAWFAY**

**VVAWFAY**

**VVATFAY**

**SSYYFDY**

**SSYYFDY**

**SSYAMDY**

**SSYAMDY**

**SSYAMDY**

**SYYAMDY**

**GYYAMDY**

**YYYAMDY**

**WGYAMDY**

###### λ5ko DFL Peritoneal Cavity B-1b Carboxy terminus

**LLAWFAY**

**NYYGYAY**

**VTTVVDY**

**RHPATAY**

**TTVVATD**

**TAVYFDY**

**GSSYFDY**

**GSSYVAY**

**GLDWFAY**

**SYWYFDV**

**RSYYFDY**

**RGWYFDV**

**YYYAMDY**

## DSP-Containing CDR-H3

### Raw Sequences

##### WT

###### WT DSP Bone Marrow Fraction E

| **ASLLLF** |
| --- |
| **ARLDYYLDY** |
| **ARDCSYYGPFAY** |
| **ARGIYYGNYPN** |
| **ARPLDGYYAY** |
| **AIHYDPFAY** |
| **ASREVYDYFAY** |
| **ARGKGYYCAMDY** |
| **ARGPDSAFAY** |
| **ARSMMVTIDY** |
| **ASPSMITTAY** |
| **AREANLLPYAMDY** |
| **ARLDGYYGFY** |
| **ARTDGFGFAY** |
| **ARHEGNPFAY** |
| **ARERDYYGNSFAY** |
| **ARHEGGNWFAY** |
| **ARHGGYDGTY** |
| **GNDYGFAY** |
| **TRDYYESAMDH** |
| **ARHGYGNYEAY** |
| **ARPEISTMITTSDY** |
| **ARHERWSYYFDY** |
| **ARHEGWLRDFDY** |
| **AREGWLHAMDY** |
| **ARDRRLLLFAY** |
| **ARLGWLRRRNY** |
| **AGTDYRYDGFAY** |
| **ARSDGYYDFDV** |
| **ARQDGNRGFAY** |
| **ARHQIYYGNPWFAY** |
| **ASIYYGNYAVDY** |
| **ASLYGSHAMDY** |
| **ARDRYGYDGFAY** |
| **AREYGYAWFAY** |
| **ARPHDGYYYFDY** |
| **ARRGLIYDGYPWFAY** |
| **ARQRLYYDYDVFDY** |
| **ARHDYRSSWFAY** |
| **AARAYRYDYFDY** |
| **ARCTRAMDY** |
| **ARSMITTGFAY** |
| **ARILMITYAMDY** |
| **APPTIGTTQFAY** |
| **ARQGWLLRAMDY** |
| **TRSVWSYHAMDY** |
| **ARHGRYSYAMDN** |
| **AREDYRYEGAMDY** |
| **ARRDGYSWYFDV** |
| **ARDYDGYLYAMDY** |
| **ARHDGNYPWFAY** |
| **ASRTDYGNTGAMDY** |
| **ARLHYGNPPYFDY** |
| **ARHIGNHWYFDV** |
| **ASLYYGNYEGVDY** |
| **ASGNPAWFAY** |
| **ARGGDYGNFYAMDY** |
| **ARQVGIYYGYDNAMDY** |
| **ARGNYGYLYAMDY** |
| **ARHDGYGYAMDY** |
| **ARHDYDYDEWFAY** |
| **ARYDYDWYFDV** |
| **ARGYDYDAWFAY** |
| **ARHSYYDYDAAMDY** |
| **ARVYDYADYFDY** |
| **ARERAYYGNYEGVAY** |
| **AREKYGNFEYFDV** |
| **ARDNYRYDYAMDY** |
| **ARLPRYDNAMDY** |
| **ARQGYDGAMDY** |
| **ARERGPTMINYYAMDY** |
| **ARNDATMITGYAMDY** |
| **ARGSTMITLYAMDY** |
| **ARHGRRDAMDY** |
| **ARGLRGFAMDY** |
| **ARAYDGYGGWYFDV** |
| **ARAIDGYYEAWFAY** |
| **ARHDYGNYNWYFDV** |
| **ARRGNYPWYFDV** |
| **ARHEGDYGNSFPWSAY** |
| **ASYGNYSYYFDY** |
| **ARHGNYPAWFAY** |
| **ARQGDGNYVSWFAY** |
| **ARHGRYYGNYVYAMDY** |
| **ARSVYYGNYEWYFDV** |
| **ARHSPGTAHYAMDY** |
| **ARHPPYGYDGAHFDY** |
| **ARCGYDGWYFDV** |
| **TRVGDGYEGYAMDY** |
| **ARDRGYDEVWFAY** |
| **ASLIYYDYDVAWFAY** |
| **ARDKDYRYDDYAMDY** |
| **ARWNRYDVKGSDY** |
| **ARGQXYRYPYXAMDY** |
| **ARWRYDVGYFDV** |
| **ARRGITKGYAMDY** |
| **AKWLLRGYAMDY** |
| **AREVRRDGAFDY** |
| **ARDGYYDPAWFAY** |
| **ARPDGYYVDWYFDV** |
| **ARQRGYDERHYFDY** |
| **ARFGYPRYWYFDV** |
| **ARLYYGYVDYYAMDY** |
| **ARYYDYDDYYAMDY** |
| **ARDGDYDGGSLMDY** |
| **ARGRAYGNYDYYAMDY** |
| **ARIWYPGDYYAMDY** |
| **ARGGSYYGNPYYYAMDY** |
| **AREKGNYGFFYAMDY** |
| **ASHYGYDEGYYATDY** |
| **ASPAYYGNYTSPYYAMDY** |

###### WT DSP Peritoneal Cavity B-2

| ARPDGAY |
| --- |
| ASYYGNY |
| ARAYYGTAY |
| ARHDYAY |
| AREGMIAMDY |
| ARAIEEAMDY |
| ARHDYRYAWFAY |
| ARGGNYDGDY |
| ARQGYGNWYFDV |
| ARHWDGNYAMDY |
| ARYGYDYFDY |
| ARYGNYGLDY |
| ARHGLYDYDGFAY |
| ARHDGYYAMDY |
| ARRGSTMMEWYFDV |
| ARLMTTTVMDY |
| ARGLRPHFDY |
| ARGVLRYFDV |
| ANLLWLEYAMDY |
| ARHEDYRYDDGFDY |
| ARERNDYDDAMDY |
| ARQRDYAYAMDY |
| ARPWDYGNYYAMDY |
| ARHYGNYYAMDY |
| ARRGDYGNFAWFAY |
| ARVDYDAWFAY |
| ARGNYYWSLYAMDY |
| AAMITTAWFAY |
| ARHGRLPYYAMDY |
| ARQGDGNYGWYFDV |
| ARHGGNYGYAMDY |
| ARYDYDVGYFDV |
| ARGIGNYVRAMDY |
| ARREITGDYAMDY |
| ARRGYDRAYAMDY |
| ARDGYDPYYAMDY |
| ARHGYDDAAWFAY |
| ARRWLLRGNYAMDY |

###### WT DSP Peritoneal Cavity B-1a

| ARGEGLRFAY |
| --- |
| ARHHGLAY |
| ARHYGYDY |
| ARHDYDY |
| ARHDYDY |
| ARHNPYGNSDY |
| ARSYYGVFDY |
| ARHGNYDY |
| ARGNFAY |
| ARLTSAY |
| ARGLRFAY |
| ARLVWYYFDY |
| ARYDYDFDY |
| AIYYGYAMDY |
| ARHDGYYFDY |
| ARHGNAMDY |
| ALSTMITPFDY |
| ARHGEVRWFAY |
| ARRYGNSRMDY |
| ARQDRYDAMDY |
| ARYRYDAMDY |
| ARHGNYAMDY |
| ARGDGLTWFAY |
| ARHDYDYFDY |
| ARQRGNAWFAY |
| ARHDGYYAMDY |
| ARHGNWYFDV |
| ARHGNYAMDY |
| ARDREYGNYWYFDV |
| ARHYYRYNWYFDV |
| ARHYYRYDWYFDV |
| ARDYYRYDYAMDY |
| ARHGNYWYFDV |
| ARYDYDYAMDY |
| ARGGYGNYDWFAY |
| ARHDGYYYAMDY |
| ARNYGNYAWFAY |
| ARGDGYDYAMDY |
| ARYDYAMDY |
| ARPGNYYAMDY |
| ARDRGLYYGYDGAMDY |
| ARWYGNYYAMDY |
| ARHAYYGNYYAMDY |
| ARGYDGYYYAMDY |
| ARSGYPYAMDY |
| ARHYDYAMDY |
| ARHGYDYAMDY |
| ARHGNYWYFDV |
| ARHGNYWYFGV |
| ARHGNYYAMDY |
| ARSTMITYYAMDY |
| ARRISTMITTGAMDY |
| ARVGLRRKDFDY |
| ARGKRNPSRAMNY |
| ARWAKYGNYEDAMDY |
| ARLYYGNYYWYFDV |
| ARHDGNLFYAMDY |
| ARHYRYDYYAMDY |
| ARYGNSRYWYFDV |
| AGQKYGNPYYYAMDY |
| AREDYDYFYYYAMDY |
| ARHYDGYYGYYAMDY |
| ARQDWLLWLRRGVSYFDY |
| ARGYGNYVGYYAMDY |
| ARGYYDVWGYYAMDY |

###### WT DSP Peritoneal Cavity B-1b

| ARYDGY |
| --- |
| AGDRGYDYFDY |
| ARRGNGYFAY |
| ARQGRYDAMDY |
| ARSYYGNFDY |
| ARHDGYFDY |
| ARHRGRTMITFAY |
| ARWYGNYFDY |
| ARSYRYAMDY |
| ARFGSYGNYFDY |
| ARPGVYGNPMDY |
| ARGGIYRYYFDY |
| ASLYGYHFDY |
| ARVYDYYAMDY |
| ARHGYYFDY |
| ARDGYSFAY |
| ARRTMIRAMDY |
| ARQGRYDGFDY |
| ARGYRYDWFAY |
| AIYYGNYWFAY |
| ARGGNYGFDY |
| ARQDRYVAMDY |
| ARSGYYPMDY |
| ASGNYAMDY |
| ARLYGNYYFDY |
| ARSGYYLMDY |
| ARHRYDAMDY |
| ASNYYFDY |
| ARHEAMVTAWFAY |
| AREVRQAMDY |
| ARRDRSYWYFDV |
| ARQGNYDWFAY |
| AREDYDYAMDY |
| ARHGYDWYFDV |
| ARDGAYYRYDGAMDY |
| ARAVYDYPAWFAY |
| ARSVYGNFYAMDY |
| TMITTYAMDY |
| TMITTYAMDH |
| ARRGWLRDYAMDY |
| ARQDGNYGGYFDV |
| ARRGYRSGYYAMDY |
| ARELYGNYYYAMDY |
| ARELYGNYYYAMDN |
| ARHDGYYGSWFAY |
| ARDAMITTGNYFDY |
| ARFYDGYYVGYAMDY |
| ARHGYYDYWYFDV |
| SRHYGNYVEYAMDY |

##### λ5ko

###### λ5ko DSP Bone Marrow Fraction E

| ARHLRDGY |
| --- |
| ARHPTRDY |
| ARDGNY |
| AREGAMIGY |
| ARQVITT |
| ARHLLYFDY |
| ARQGPYYGNLDY |
| ARLDDYAYY |
| ARIFYDGYCYY |
| ARQGMVTTDY |
| ARGGPTMITFAY |
| ARDGDYGNYGAY |
| ARDPGGNYPSY |
| ARHGVRDYLDY |
| ARSMVTTFDY |
| ARHEGVRVRLLAMDY |
| ARQGYDYAWFAY |
| ARHGDYGAMDY |
| ARGGIYGKRAMDY |
| ARSPLYDAMDY |
| ARDALYGYDVIAY |
| ARDPTIGTAWFAY |
| AFMVTEGFAY |
| ARLGITSAMDY |
| AGVMITTAMDY |
| ARGVRDSMDY |
| ARGWSGYAMDY |
| ARRDGGLLLWFAY |
| ASHDGGFAY |
| ARGAYYRYDGAMDY |
| ARTPYGNYGAMDY |
| ARTADLGDYPYAMDY |
| ARPMMVTHWYFDV |
| ARGAMITTAWFAY |
| ARGRGLRRDWFAY |
| ARDGGLRRWYFDV |
| ARHRGLRRPPAMDY |
| ARRGLRYYYAMDY |
| ARRGLPYYYAMDY |
| ARAVRAYYAMDY |
| ARDGRDYDRAAWFAY |
| ARGGTPYYYAMDY |
| ARQEVTTGYYAMDY |
| ARQGVTTGYYAMDY |
| ARQGVTTGCYAMDY |
| ARSMVTGGYYAMDY |

###### λ5ko DSP Peritoneal Cavity B-2

| ARQAIYYGN |
| --- |
| ARHDRFAY |
| ARHGGPTIGFDY |
| ARGGSLVWSFAY |
| ARDAYYGNLAY |
| ARQEGMVITFVY |
| ARSTPAWFAY |
| ARHGDYDAVY |
| ARGHYGNYEDY |
| ARRRDGKGLAY |
| ARGDYRYDFDY |
| ARTIYDGYILDY |
| ARGTMIFWFAY |
| ARLWXLNFDY |
| ARRWLRRFDY |
| AREVWPAWFAY |
| ARRLYGYSWFAY |
| ARHRYDAMDY |
| ARQDYGNYGFAY |
| ARAYYRYDGFAY |
| ARGNGKDAMDY |
| ARGRGYGNFWFAY |
| ARHGNYAMDY |
| ASRYRRYAMDY |
| ARRGDYPLFAY |
| ARTGHYDYVYFDY |
| ARDPTIGTAWFAY |
| ARDLKSMVTGGFAY |
| ARASTMITGWFAY |
| ARGITNYFDY |
| ARVVRGYFDV |
| ARHVGKGAWFAY |
| ARHDYGNAMDY |
| ARRYDYAMDY |
| ARHNRYDDAMDY |
| ARHERSGDYDYAMDY |
| ARRGNYRYDGAMDY |
| ARHGYDWYFDV |
| ARPLLRYGGWFAY |
| ARGGKDYAMDY |
| ARDNYRYAWYFHV |
| ARNGNYPWFAY |
| ARSYYRYDGGFAY |
| ARRGPTMVTPYAMDY |
| ARGRSTMITTGVSDY |
| ARHRTMITAGAMDY |
| ARDHKYGNYGKRFAY |
| ARSNRYDEAWFAY |
| AGRDYRYDGAWFAY |
| ARGRWYDHGGFAY |
| ARGDDYDVGAMDY |
| ARSIGTRGYYFDY |
| ARSLRRGYAMDY |
| ARDDYDRHFAMDY |
| ARDDYDRHFAMDY |

###### λ5ko DSP Peritoneal Cavity B-1a

| ARSTMETY |
| --- |
| ARHGNYY |
| ARHGNFAY |
| ARHYFYAMDY |
| ARLMITTPY |
| ARDIGTFDY |
| ARWLLRAY |
| ARHGNAMDY |
| ARGYYDYDFDY |
| ASPWGDYAFAY |
| ARGDGYLLDY |
| ARGSYYGNYVAY |
| ARDSMVTTMDY |
| ARHMVTTFAY |
| ARSTPSMVIYFDY |
| MVTTFAY |
| ARTIGTTFAY |
| ARHGLRRFAY |
| ARPRPFAY |
| ARWLLTMDY |
| ARDGYYAMDY |
| ARGGIYGKRAMDY |
| ARRYGKRAMDY |
| ARHDYDWFAY |
| ARQRGNYAFAY |
| ARDDGYYAMDY |
| ARETNYYFLYAMDY |
| ARSMIYYDYSWFAY |
| ARPEMVTAWFAY |
| ARHVVTTAMDY |
| ARHRGIYAMDY |
| ARSMVTAWFAY |
| ARHARLRQGFAY |
| ARHGRLRRAFAY |
| ARHGNYYAMDY |
| ARHYGYDYAMDY |
| ARDGNYWYFDV |
| ARVYDGYGAWFAY |
| ARHANDYDYAMDY |
| ARLHYDYDYAMDY |
| ARDRYDYAMDY |
| ARYRYDYAMDY |
| ARHRYDYAMDY |
| ARHYRYYYAMDY |
| ARHGNYWYFDV |
| ARHGYDWYFDV |
| ARHGNYWYFDV |
| ARPPYYDYLAWFAY |
| ATYYGNYYAMDY |
| ARGDGYYYAMDY |
| ARYGYDWCFDV |
| ARDGNFAWFAY |
| ARHTMVTTYYFDY |
| ARQTIGTTRAMDY |
| ARSTMITTYYFDY |
| ARHTRVRYYAMDY |
| ARYAFAYYRYDGGYFDY |
| ARHERYDEAWFAY |
| ARPSLYRYDGAWFAY |
| ARDYDYDLAWFAY |
| ARHYRYDDYYAMDY |
| ARHGYDDYWYFDV |
| ARHPQTLYDYDGGAWFAY |
| ARHEGRYDVFYAMDY |

###### λ5ko DSP Peritoneal Cavity B-1b

| ARFYDGYY |
| --- |
| ARHGSMIFAY |
| ASSSTMITY |
| ARGYGNFDY |
| AAYGNFDY |
| ARGNYPYFDV |
| ARSRGYYYGSSDY |
| ARSGRSTMIPFAY |
| ARSIPNLMVNFDY |
| ARSIPNLMVNFGY |
| ARVLVWYLFDY |
| ARRLLWYYFDY |
| ARDWDYDYWFAY |
| ARGGDYVFAY |
| ARFYEGAFDD |
| ARHDYGFAY |
| ARGYYDYDFDY |
| ARSRDGYYFDY |
| MMGSAMDY |
| ARQRDGLRGFAY |
| ARHDYRYAWFAY |
| AREEGYGYPWFAY |
| ASNGNYVFAY |
| ARGYDAMDY |
| ARGGIYGKRAMDY |
| ASGNYDFDY |
| ARHKYGNYEFAY |
| ASYYGNRGFAY |
| ARRGDDYEFFDY |
| ARSGYRSPWFAY |
| ARKGISDYPWFAY |
| ARSEGYGNYVFDY |
| ARWDGLYAMDY |
| TREAMVNYAMDY |
| ARHWGVTPWFAY |
| ARHSMVTSWFAY |
| ARIDMIRGAMDY |
| ARGKELLLFAY |
| ARRDYGNFWYFDV |
| ARHYGNRYAMDY |
| ARDRSYGNLAWFAY |
| ARDLGYGNLAWFAY |
| ARSGNGYALYFDY |
| ARSYSMVTSYYFDY |
| ARLWLPPYAMDY |
| ALLRRYAMDY |
| ARDTALGNYPAWFAY |
| AANYDGAWFAY |
| ARHPLYDGYYVGAMDY |
| ARNYRSHYYAMDY |
| ARHGGGTTDDGGAY |
| ARMVKRVAWFAY |
| ARRIGTTRVAMDY |
| ARLRRRAWFAY |
| ARHGGLRRSYAMDY |
| ARHGGLRRSYAMGY |
| ARRDDYDEKAWFAY |
| ARNYRYDGGYYFDY |
| ARATMVTTGYWYFDV |
| AKWLRRGYYAMDY |

### Counting from the amino terminus end.

##### WT

###### WT DSP Bone Marrow Fraction E Amino Terminus

**ASLLLF-**

**ARLDYYL**

**ARDCSYY**

**ARGIYYG**

**ARPLDGY**

**AIHYDPF**

**ASREVYD**

**ARGKGYY**

**ARGPDSA**

**ARSMMVT**

**ASPSMIT**

**AREANLL**

**ARLDGYY**

**ARTDGFG**

**ARHEGNP**

**ARERDYY**

**ARHEGGN**

**ARHGGYD**

**GNDYGFA**

**TRDYYES**

**ARHGYGN**

**ARPEIST**

**ARHERWS**

**ARHEGWL**

**AREGWLH**

**ARDRRLL**

**ARLGWLR**

**AGTDYRY**

**ARSDGYY**

**ARQDGNR**

**ARHQIYY**

**ASIYYGN**

**ASLYGSH**

**ARDRYGY**

**AREYGYA**

**ARPHDGY**

**ARRGLIY**

**ARQRLYY**

**ARHDYRS**

**AARAYRY**

**ARCTRAM**

**ARSMITT**

**ARILMIT**

**APPTIGT**

**ARQGWLL**

**TRSVWSY**

**ARHGRYS**

**AREDYRY**

**ARRDGYS**

**ARDYDGY**

**ARHDGNY**

**ASRTDYG**

**ARLHYGN**

**ARHIGNH**

**ASLYYGN**

**ASGNPAW**

**ARGGDYG**

**ARQVGIY**

**ARGNYGY**

**ARHDGYG**

**ARHDYDY**

**ARYDYDW**

**ARGYDYD**

**ARHSYYD**

**ARVYDYA**

**ARERAYY**

**AREKYGN**

**ARDNYRY**

**ARLPRYD**

**ARQGYDG**

**ARERGPT**

**ARNDATM**

**ARGSTMI**

**ARHGRRD**

**ARGLRGF**

**ARAYDGY**

**ARAIDGY**

**ARHDYGN**

**ARRGNYP**

**ARHEGDY**

**ASYGNYS**

**ARHGNYP**

**ARQGDGN**

**ARHGRYY**

**ARSVYYG**

**ARHSPGT**

**ARHPPYG**

**ARCGYDG**

**TRVGDGY**

**ARDRGYD**

**ASLIYYD**

**ARDKDYR**

**ARWNRYD**

**ARGQXYR**

**ARWRYDV**

**ARRGITK**

**AKWLLRG**

**AREVRRD**

**ARDGYYD**

**ARPDGYY**

**ARQRGYD**

**ARFGYPR**

**ARLYYGY**

**ARYYDYD**

**ARDGDYD**

**ARGRAYG**

**ARIWYPG**

**ARGGSYY**

**AREKGNY**

**ASHYGYD**

**ASPAYYG**

###### WT DSP Peritoneal Cavity B-2 Amino Terminus

**ARPDGAY**

**ASYYGNY**

**ARAYYGT**

**ARHDYAY**

**AREGMIA**

**ARAIEEA**

**ARHDYRY**

**ARGGNYD**

**ARQGYGN**

**ARHWDGN**

**ARYGYDY**

**ARYGNYG**

**ARHGLYD**

**ARHDGYY**

**ARRGSTM**

**ARLMTTT**

**ARGLRPH**

**ARGVLRY**

**ANLLWLE**

**ARHEDYR**

**ARERNDY**

**ARQRDYA**

**ARPWDYG**

**ARHYGNY**

**ARRGDYG**

**ARVDYDA**

**ARGNYYW**

**AAMITTA**

**ARHGRLP**

**ARQGDGN**

**ARHGGNY**

**ARYDYDV**

**ARGIGNY**

**ARREITG**

**ARRGYDR**

**ARDGYDP**

**ARHGYDD**

**ARRWLLR**

###### WT DSP Peritoneal Cavity B-1a Amino Terminus

**ARGEGLR**

**ARHHGLA**

**ARHYGYD**

**ARHDYDY**

**ARHDYDY**

**ARHNPYG**

**ARSYYGV**

**ARHGNYD**

**ARGNFAY**

**ARLTSAY**

**ARGLRFA**

**ARLVWYY**

**ARYDYDF**

**AIYYGYA**

**ARHDGYY**

**ARHGNAM**

**ALSTMIT**

**ARHGEVR**

**ARRYGNS**

**ARQDRYD**

**ARYRYDA**

**ARHGNYA**

**ARGDGLT**

**ARHDYDY**

**ARQRGNA**

**ARHDGYY**

**ARHGNWY**

**ARHGNYA**

**ARDREYG**

**ARHYYRY**

**ARHYYRY**

**ARDYYRY**

**ARHGNYW**

**ARYDYDY**

**ARGGYGN**

**ARHDGYY**

**ARNYGNY**

**ARGDGYD**

**ARYDYAM**

**ARPGNYY**

**ARDRGLY**

**ARWYGNY**

**ARHAYYG**

**ARGYDGY**

**ARSGYPY**

**ARHYDYA**

**ARHGYDY**

**ARHGNYW**

**ARHGNYW**

**ARHGNYY**

**ARSTMIT**

**ARRISTM**

**ARVGLRR**

**ARGKRNP**

**ARWAKYG**

**ARLYYGN**

**ARHDGNL**

**ARHYRYD**

**ARYGNSR**

**AGQKYGN**

**AREDYDY**

**ARHYDGY**

**ARQDWLL**

**ARGYGNY**

**ARGYYDV**

###### WT DSP Peritoneal Cavity B-1b Amino Terminus

**ARYDGY-**

**AGDRGYD**

**ARRGNGY**

**ARQGRYD**

**ARSYYGN**

**ARHDGYF**

**ARHRGRT**

**ARWYGNY**

**ARSYRYA**

**ARFGSYG**

**ARPGVYG**

**ARGGIYR**

**ASLYGYH**

**ARVYDYY**

**ARHGYYF**

**ARDGYSF**

**ARRTMIR**

**ARQGRYD**

**ARGYRYD**

**AIYYGNY**

**ARGGNYG**

**ARQDRYV**

**ARSGYYP**

**ASGNYAM**

**ARLYGNY**

**ARSGYYL**

**ARHRYDA**

**ASNYYFD**

**ARHEAMV**

**AREVRQA**

**ARRDRSY**

**ARQGNYD**

**AREDYDY**

**ARHGYDW**

**ARDGAYY**

**ARAVYDY**

**ARSVYGN**

**TMITTYA**

**TMITTYA**

**ARRGWLR**

**ARQDGNY**

**ARRGYRS**

**ARELYGN**

**ARELYGN**

**ARHDGYY**

**ARDAMIT**

**ARFYDGY**

**ARHGYYD**

**SRHYGNY**

##### λ5ko

###### λ5ko DSP Bone Marrow Fraction E Amino Terminus

**ARHLRDG**

**ARHPTRD**

**ARDGNY-**

**AREGAMI**

**ARQVITT**

**ARHLLYF**

**ARQGPYY**

**ARLDDYA**

**ARIFYDG**

**ARQGMVT**

**ARGGPTM**

**ARDGDYG**

**ARDPGGN**

**ARHGVRD**

**ARSMVTT**

**ARHEGVR**

**ARQGYDY**

**ARHGDYG**

**ARGGIYG**

**ARSPLYD**

**ARDALYG**

**ARDPTIG**

**AFMVTEG**

**ARLGITS**

**AGVMITT**

**ARGVRDS**

**ARGWSGY**

**ARRDGGL**

**ASHDGGF**

**ARGAYYR**

**ARTPYGN**

**ARTADLG**

**ARPMMVT**

**ARGAMIT**

**ARGRGLR**

**ARDGGLR**

**ARHRGLR**

**ARRGLRY**

**ARRGLPY**

**ARAVRAY**

**ARDGRDY**

**ARGGTPY**

**ARQEVTT**

**ARQGVTT**

**ARQGVTT**

**ARSMVTG**

###### λ5ko DSP Peritoneal Cavity B-2 Amino Terminus

**ARQAIYY**

**ARHDRFA**

**ARHGGPT**

**ARGGSLV**

**ARDAYYG**

**ARQEGMV**

**ARSTPAW**

**ARHGDYD**

**ARGHYGN**

**ARRRDGK**

**ARGDYRY**

**ARTIYDG**

**ARGTMIF**

**ARLWXLN**

**ARRWLRR**

**AREVWPA**

**ARRLYGY**

**ARHRYDA**

**ARQDYGN**

**ARAYYRY**

**ARGNGKD**

**ARGRGYG**

**ARHGNYA**

**ASRYRRY**

**ARRGDYP**

**ARTGHYD**

**ARDPTIG**

**ARDLKSM**

**ARASTMI**

**ARGITNY**

**ARVVRGY**

**ARHVGKG**

**ARHDYGN**

**ARRYDYA**

**ARHNRYD**

**ARHERSG**

**ARRGNYR**

**ARHGYDW**

**ARPLLRY**

**ARGGKDY**

**ARDNYRY**

**ARNGNYP**

**ARSYYRY**

**ARRGPTM**

**ARGRSTM**

**ARHRTMI**

**ARDHKYG**

**ARSNRYD**

**AGRDYRY**

**ARGRWYD**

**ARGDDYD**

**ARSIGTR**

**ARSLRRG**

**ARDDYDR**

**ARDDYDR**

###### λ5ko DSP Peritoneal Cavity B-1a Amino Terminus

**ARSTMET**

**ARHGNYY**

**ARHGNFA**

**ARHYFYA**

**ARLMITT**

**ARDIGTF**

**ARWLLRA**

**ARHGNAM**

**ARGYYDY**

**ASPWGDY**

**ARGDGYL**

**ARGSYYG**

**ARDSMVT**

**ARHMVTT**

**ARSTPSM**

**MVTTFAY**

**ARTIGTT**

**ARHGLRR**

**ARPRPFA**

**ARWLLTM**

**ARDGYYA**

**ARGGIYG**

**ARRYGKR**

**ARHDYDW**

**ARQRGNY**

**ARDDGYY**

**ARETNYY**

**ARSMIYY**

**ARPEMVT**

**ARHVVTT**

**ARHRGIY**

**ARSMVTA**

**ARHARLR**

**ARHGRLR**

**ARHGNYY**

**ARHYGYD**

**ARDGNYW**

**ARVYDGY**

**ARHANDY**

**ARLHYDY**

**ARDRYDY**

**ARYRYDY**

**ARHRYDY**

**ARHYRYY**

**ARHGNYW**

**ARHGYDW**

**ARHGNYW**

**ARPPYYD**

**ATYYGNY**

**ARGDGYY**

**ARYGYDW**

**ARDGNFA**

**ARHTMVT**

**ARQTIGT**

**ARSTMIT**

**ARHTRVR**

**ARYAFAY**

**ARHERYD**

**ARPSLYR**

**ARDYDYD**

**ARHYRYD**

**ARHGYDD**

**ARHPQTL**

**ARHEGRY**

###### λ5ko DSP Peritoneal Cavity B-1b Amino Terminus

**ARFYDGY**

**ARHGSMI**

**ASSSTMI**

**ARGYGNF**

**AAYGNFD**

**ARGNYPY**

**ARSRGYY**

**ARSGRST**

**ARSIPNL**

**ARSIPNL**

**ARVLVWY**

**ARRLLWY**

**ARDWDYD**

**ARGGDYV**

**ARFYEGA**

**ARHDYGF**

**ARGYYDY**

**ARSRDGY**

**MMGSAMD**

**ARQRDGL**

**ARHDYRY**

**AREEGYG**

**ASNGNYV**

**ARGYDAM**

**ARGGIYG**

**ASGNYDF**

**ARHKYGN**

**ASYYGNR**

**ARRGDDY**

**ARSGYRS**

**ARKGISD**

**ARSEGYG**

**ARWDGLY**

**TREAMVN**

**ARHWGVT**

**ARHSMVT**

**ARIDMIR**

**ARGKELL**

**ARRDYGN**

**ARHYGNR**

**ARDRSYG**

**ARDLGYG**

**ARSGNGY**

**ARSYSMV**

**ARLWLPP**

**ALLRRYA**

**ARDTALG**

**AANYDGA**

**ARHPLYD**

**ARNYRSH**

**ARHGGGT**

**ARMVKRV**

**ARRIGTT**

**ARLRRRA**

**ARHGGLR**

**ARHGGLR**

**ARRDDYD**

**ARNYRYD**

**ARATMVT**

**AKWLRRG**

### Counting from the carboxy terminus end.

##### WT

###### WT DSP Bone Marrow Fraction E Carboxy terminus

**-ASLLLF**

**LDYYLDY**

**YYGPFAY**

**YYGNYPN**

**LDGYYAY**

**HYDPFAY**

**VYDYFAY**

**YYCAMDY**

**PDSAFAY**

**MMVTIDY**

**SMITTAY**

**LPYAMDY**

**DGYYGFY**

**DGFGFAY**

**EGNPFAY**

**YGNSFAY**

**GGNWFAY**

**GGYDGTY**

**NDYGFAY**

**YESAMDH**

**YGNYEAY**

**MITTSDY**

**WSYYFDY**

**WLRDFDY**

**WLHAMDY**

**RLLLFAY**

**WLRRRNY**

**RYDGFAY**

**GYYDFDV**

**GNRGFAY**

**GNPWFAY**

**GNYAVDY**

**GSHAMDY**

**GYDGFAY**

**GYAWFAY**

**GYYYFDY**

**GYPWFAY**

**DYDVFDY**

**RSSWFAY**

**RYDYFDY**

**CTRAMDY**

**ITTGFAY**

**ITYAMDY**

**GTTQFAY**

**LLRAMDY**

**SYHAMDY**

**YSYAMDN**

**YEGAMDY**

**YSWYFDV**

**YLYAMDY**

**NYPWFAY**

**NTGAMDY**

**NPPYFDY**

**NHWYFDV**

**NYEGVDY**

**NPAWFAY**

**NFYAMDY**

**YDNAMDY**

**YLYAMDY**

**YGYAMDY**

**YDEWFAY**

**YDWYFDV**

**YDAWFAY**

**YDAAMDY**

**YADYFDY**

**NYEGVAY**

**NFEYFDV**

**YDYAMDY**

**YDNAMDY**

**YDGAMDY**

**NYYAMDY**

**TGYAMDY**

**TLYAMDY**

**RRDAMDY**

**RGFAMDY**

**GGWYFDV**

**YEAWFAY**

**YNWYFDV**

**YPWYFDV**

**SFPWSAY**

**YSYYFDY**

**YPAWFAY**

**YVSWFAY**

**YVYAMDY**

**YEWYFDV**

**AHYAMDY**

**DGAHFDY**

**DGWYFDV**

**EGYAMDY**

**DEVWFAY**

**DVAWFAY**

**DDYAMDY**

**DVKGSDY**

**PYXAMDY**

**DVGYFDV**

**KGYAMDY**

**RGYAMDY**

**RDGAFDY**

**DPAWFAY**

**VDWYFDV**

**ERHYFDY**

**RYWYFDV**

**DYYAMDY**

**DYYAMDY**

**GGSLMDY**

**DYYAMDY**

**DYYAMDY**

**YYYAMDY**

**FFYAMDY**

**GYYATDY**

**PYYAMDY**

###### WT DSP Peritoneal Cavity B-2 Carboxy terminus

**ARPDGAY**

**ASYYGNY**

**AYYGTAY**

**ARHDYAY**

**GMIAMDY**

**IEEAMDY**

**RYAWFAY**

**GNYDGDY**

**GNWYFDV**

**GNYAMDY**

**GYDYFDY**

**GNYGLDY**

**DYDGFAY**

**GYYAMDY**

**MEWYFDV**

**TTTVMDY**

**LRPHFDY**

**VLRYFDV**

**LEYAMDY**

**YDDGFDY**

**YDDAMDY**

**YAYAMDY**

**NYYAMDY**

**NYYAMDY**

**NFAWFAY**

**YDAWFAY**

**SLYAMDY**

**TTAWFAY**

**PYYAMDY**

**YGWYFDV**

**YGYAMDY**

**DVGYFDV**

**YVRAMDY**

**GDYAMDY**

**RAYAMDY**

**PYYAMDY**

**DAAWFAY**

**GNYAMDY**

###### WT DSP Peritoneal Cavity B-1a Carboxy terminus

**EGLRFAY**

**RHHGLAY**

**RHYGYDY**

**ARHDYDY**

**ARHDYDY**

**PYGNSDY**

**YYGVFDY**

**RHGNYDY**

**ARGNFAY**

**ARLTSAY**

**RGLRFAY**

**VWYYFDY**

**YDYDFDY**

**YGYAMDY**

**DGYYFDY**

**HGNAMDY**

**MITPFDY**

**EVRWFAY**

**GNSRMDY**

**RYDAMDY**

**RYDAMDY**

**GNYAMDY**

**GLTWFAY**

**DYDYFDY**

**GNAWFAY**

**GYYAMDY**

**GNWYFDV**

**GNYAMDY**

**NYWYFDV**

**YNWYFDV**

**YDWYFDV**

**YDYAMDY**

**NYWYFDV**

**YDYAMDY**

**NYDWFAY**

**YYYAMDY**

**NYAWFAY**

**YDYAMDY**

**YDYAMDY**

**NYYAMDY**

**YDGAMDY**

**NYYAMDY**

**NYYAMDY**

**YYYAMDY**

**YPYAMDY**

**YDYAMDY**

**YDYAMDY**

**NYWYFDV**

**NYWYFGV**

**NYYAMDY**

**TYYAMDY**

**TTGAMDY**

**RRKDFDY**

**PSRAMNY**

**YEDAMDY**

**YYWYFDV**

**LFYAMDY**

**DYYAMDY**

**RYWYFDV**

**YYYAMDY**

**YYYAMDY**

**GYYAMDY**

**GVSYFDY**

**GYYAMDY**

**GYYAMDY**

###### WT DSP Peritoneal Cavity B-1b Carboxy terminus

**-ARYDGY**

**GYDYFDY**

**GNGYFAY**

**RYDAMDY**

**YYGNFDY**

**HDGYFDY**

**TMITFAY**

**YGNYFDY**

**YRYAMDY**

**YGNYFDY**

**YGNPMDY**

**YRYYFDY**

**YGYHFDY**

**DYYAMDY**

**HGYYFDY**

**DGYSFAY**

**MIRAMDY**

**RYDGFDY**

**RYDWFAY**

**GNYWFAY**

**GNYGFDY**

**RYVAMDY**

**GYYPMDY**

**GNYAMDY**

**GNYYFDY**

**GYYLMDY**

**RYDAMDY**

**SNYYFDY**

**VTAWFAY**

**VRQAMDY**

**SYWYFDV**

**NYDWFAY**

**YDYAMDY**

**YDWYFDV**

**YDGAMDY**

**YPAWFAY**

**NFYAMDY**

**TTYAMDY**

**TTYAMDH**

**RDYAMDY**

**YGGYFDV**

**GYYAMDY**

**YYYAMDY**

**YYYAMDN**

**YGSWFAY**

**TGNYFDY**

**VGYAMDY**

**DYWYFDV**

**VEYAMDY**

##### λ5ko

###### λ5ko DSP Bone Marrow Fraction E Carboxy terminus

**RHLRDGY**

**RHPTRDY**

**-ARDGNY**

**EGAMIGY**

**ARQVITT**

**HLLYFDY**

**YYGNLDY**

**LDDYAYY**

**YDGYCYY**

**GMVTTDY**

**TMITFAY**

**YGNYGAY**

**GGNYPSY**

**VRDYLDY**

**MVTTFDY**

**RLLAMDY**

**DYAWFAY**

**DYGAMDY**

**GKRAMDY**

**LYDAMDY**

**GYDVIAY**

**GTAWFAY**

**VTEGFAY**

**ITSAMDY**

**ITTAMDY**

**VRDSMDY**

**SGYAMDY**

**LLLWFAY**

**HDGGFAY**

**YDGAMDY**

**NYGAMDY**

**YPYAMDY**

**THWYFDV**

**TTAWFAY**

**RRDWFAY**

**RRWYFDV**

**RPPAMDY**

**YYYAMDY**

**YYYAMDY**

**AYYAMDY**

**RAAWFAY**

**YYYAMDY**

**GYYAMDY**

**GYYAMDY**

**GCYAMDY**

**GYYAMDY**

###### λ5ko DSP Peritoneal Cavity B-2 Carboxy terminus

**QAIYYGN**

**RHDRFAY**

**PTIGFDY**

**LVWSFAY**

**YYGNLAY**

**MVITFVY**

**TPAWFAY**

**GDYDAVY**

**YGNYEDY**

**DGKGLAY**

**YRYDFDY**

**DGYILDY**

**MIFWFAY**

**WXLNFDY**

**WLRRFDY**

**WPAWFAY**

**GYSWFAY**

**RYDAMDY**

**GNYGFAY**

**RYDGFAY**

**GKDAMDY**

**GNFWFAY**

**GNYAMDY**

**RRYAMDY**

**DYPLFAY**

**DYVYFDY**

**GTAWFAY**

**VTGGFAY**

**ITGWFAY**

**ITNYFDY**

**VRGYFDV**

**KGAWFAY**

**YGNAMDY**

**YDYAMDY**

**YDDAMDY**

**YDYAMDY**

**YDGAMDY**

**YDWYFDV**

**YGGWFAY**

**KDYAMDY**

**YAWYFHV**

**NYPWFAY**

**YDGGFAY**

**TPYAMDY**

**TTGVSDY**

**TAGAMDY**

**YGKRFAY**

**DEAWFAY**

**DGAWFAY**

**DHGGFAY**

**DVGAMDY**

**RGYYFDY**

**RGYAMDY**

**RHFAMDY**

**RHFAMDY**

###### λ5ko DSP Peritoneal Cavity B-1a Carboxy terminus

**RSTMETY**

**ARHGNYY**

**RHGNFAY**

**YFYAMDY**

**LMITTPY**

**DIGTFDY**

**RWLLRAY**

**HGNAMDY**

**YDYDFDY**

**GDYAFAY**

**DGYLLDY**

**YGNYVAY**

**MVTTMDY**

**MVTTFAY**

**MVIYFDY**

**MVTTFAY**

**IGTTFAY**

**GLRRFAY**

**RPRPFAY**

**WLLTMDY**

**GYYAMDY**

**GKRAMDY**

**GKRAMDY**

**DYDWFAY**

**GNYAFAY**

**GYYAMDY**

**FLYAMDY**

**DYSWFAY**

**VTAWFAY**

**VTTAMDY**

**GIYAMDY**

**VTAWFAY**

**LRQGFAY**

**LRRAFAY**

**NYYAMDY**

**YDYAMDY**

**NYWYFDV**

**YGAWFAY**

**YDYAMDY**

**YDYAMDY**

**YDYAMDY**

**YDYAMDY**

**YDYAMDY**

**YYYAMDY**

**NYWYFDV**

**YDWYFDV**

**NYWYFDV**

**YLAWFAY**

**NYYAMDY**

**YYYAMDY**

**YDWCFDV**

**NFAWFAY**

**TTYYFDY**

**TTRAMDY**

**TTYYFDY**

**RYYAMDY**

**DGGYFDY**

**DEAWFAY**

**DGAWFAY**

**DLAWFAY**

**DYYAMDY**

**DYWYFDV**

**GGAWFAY**

**VFYAMDY**

###### λ5ko DSP Peritoneal Cavity B-1b Carboxy terminus

**RFYDGYY**

**GSMIFAY**

**SSTMITY**

**GYGNFDY**

**AYGNFDY**

**NYPYFDV**

**YYGSSDY**

**TMIPFAY**

**LMVNFDY**

**LMVNFGY**

**VWYLFDY**

**LWYYFDY**

**YDYWFAY**

**GDYVFAY**

**YEGAFDD**

**HDYGFAY**

**YDYDFDY**

**DGYYFDY**

**MGSAMDY**

**GLRGFAY**

**RYAWFAY**

**GYPWFAY**

**GNYVFAY**

**GYDAMDY**

**GKRAMDY**

**GNYDFDY**

**GNYEFAY**

**GNRGFAY**

**DYEFFDY**

**RSPWFAY**

**DYPWFAY**

**GNYVFDY**

**GLYAMDY**

**VNYAMDY**

**VTPWFAY**

**VTSWFAY**

**IRGAMDY**

**ELLLFAY**

**NFWYFDV**

**NRYAMDY**

**NLAWFAY**

**NLAWFAY**

**YALYFDY**

**TSYYFDY**

**PPYAMDY**

**RRYAMDY**

**YPAWFAY**

**DGAWFAY**

**YVGAMDY**

**HYYAMDY**

**TDDGGAY**

**RVAWFAY**

**TRVAMDY**

**RRAWFAY**

**RSYAMDY**

**RSYAMGY**

**EKAWFAY**

**GGYYFDY**

**GYWYFDV**

**GYYAMDY**

# Figures

## All Sequences

### Counting from the amino terminus end.

**All Sequences**

***Amino Terminus of CDR-H3***

**Wild type λ5ko**

**Fxn E**


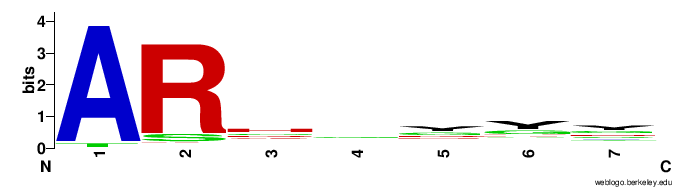

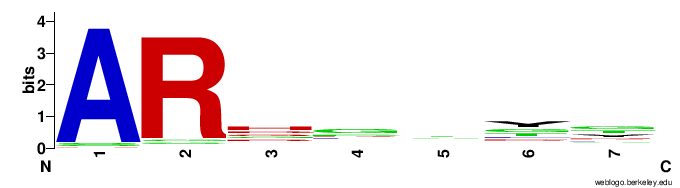


**B-2**


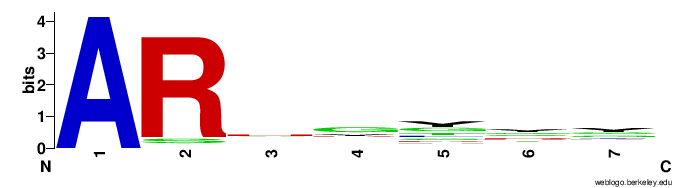

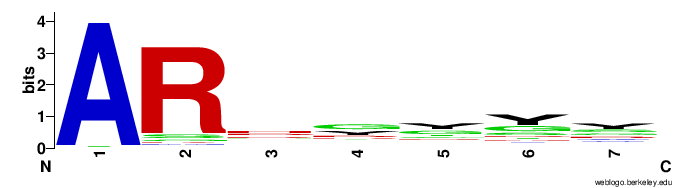


**B-1a**


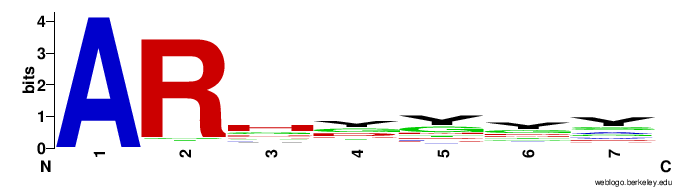

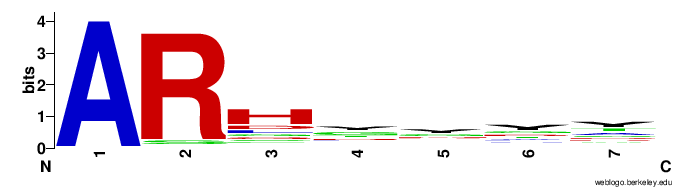


**B-1b**


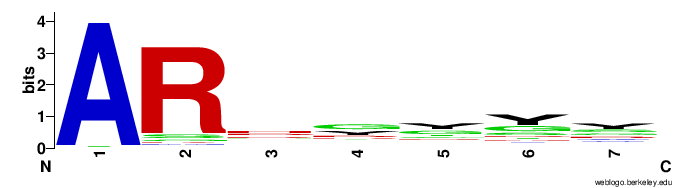

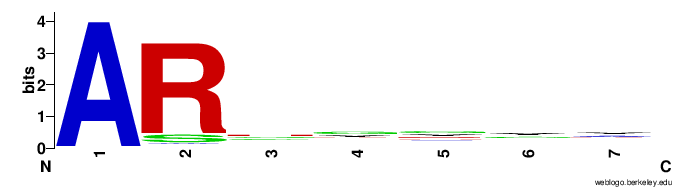


(Picture size 60% height, 45% width of original)

**All Sequences**

**Frequency Plot**

***Amino Terminus of CDR-H3***

**Wild type λ5ko**

**Fxn E**


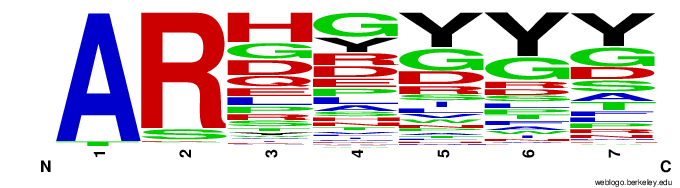

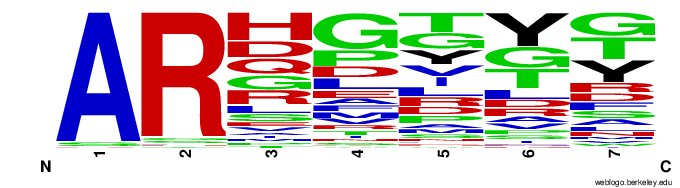


**B-2**


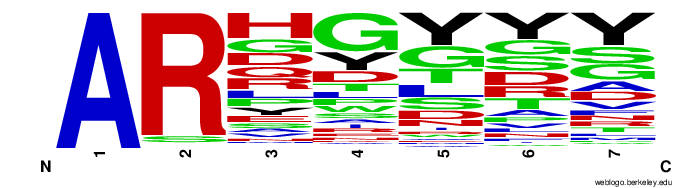

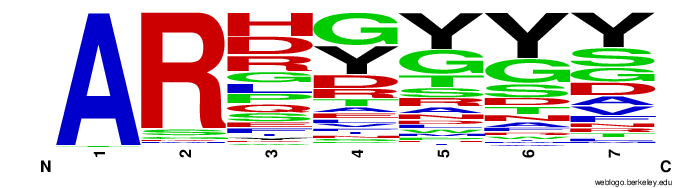


**B-1a**


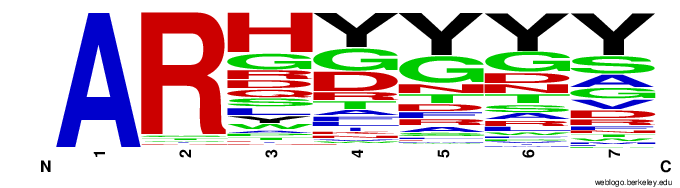

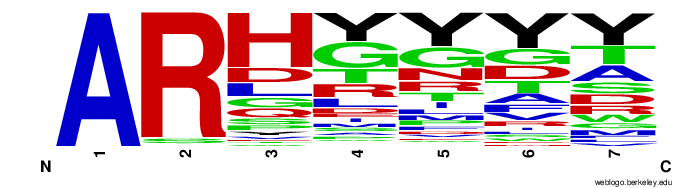


**B-1b**


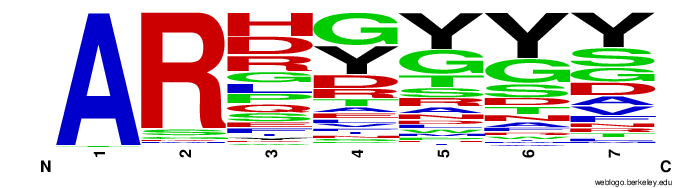

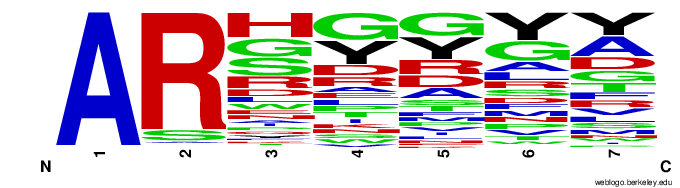


(Picture size 60% height, 45% width of original)

### Counting from the Carboxy terminus end.

**All Sequences**

***Carboxy Terminus of CDR-H3***

**Wild type λ5ko**

**Fxn E**


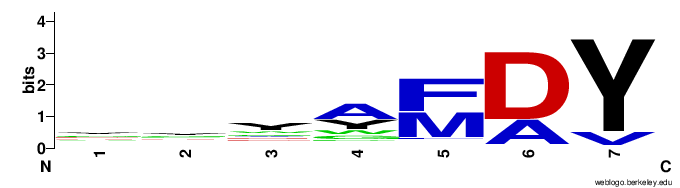

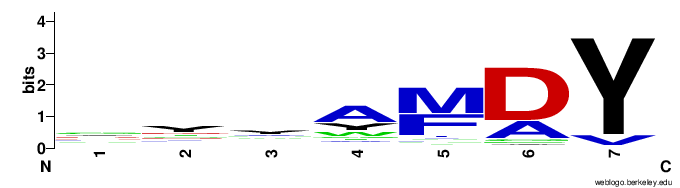


**B-2**


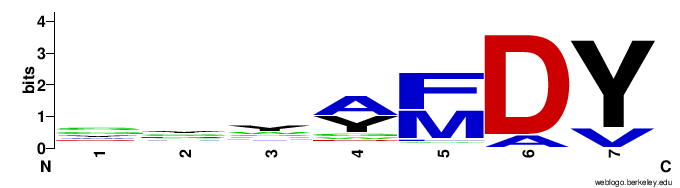

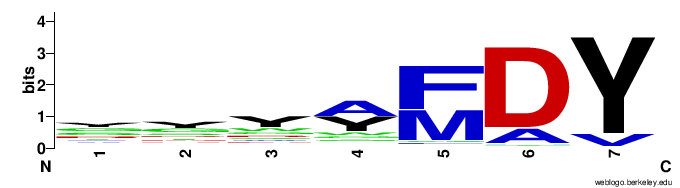


**B-1a**


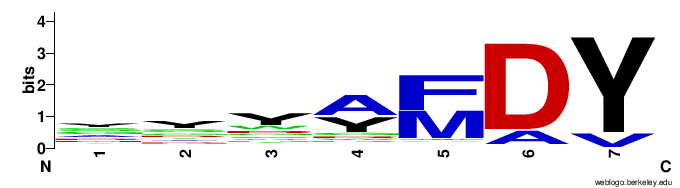

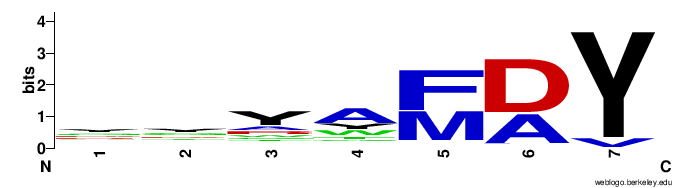


**B-1b**


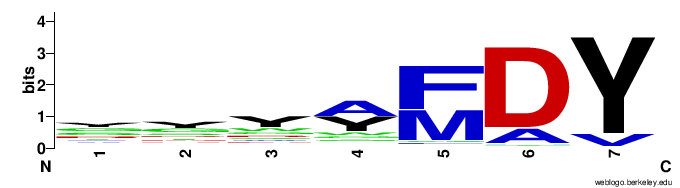

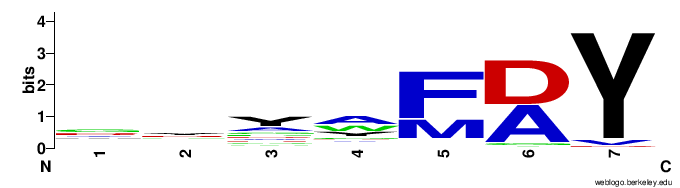


(Picture size 60% height, 45% width of original)

**All Sequences**

**Frequency Plot**

***Carboxy Terminus of CDR-H3***

**Wild type λ5ko**

**Fxn E**


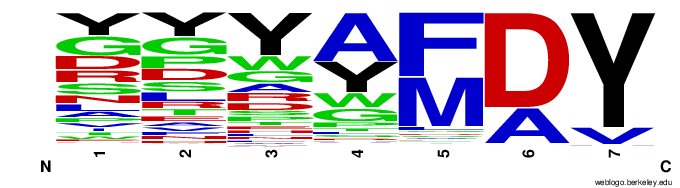

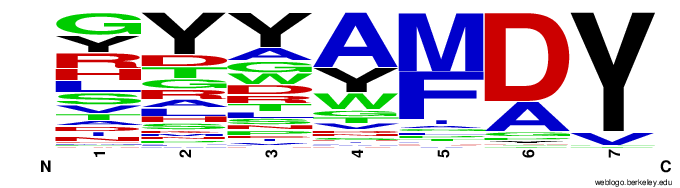


**B-2**


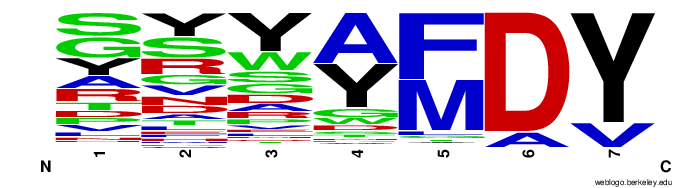

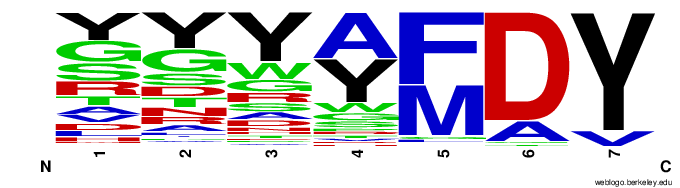


**B-1a**


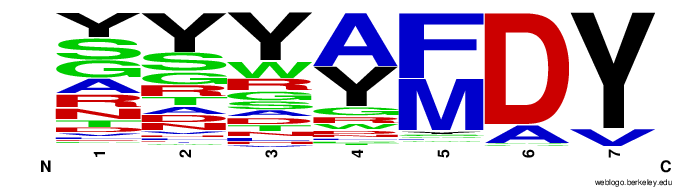

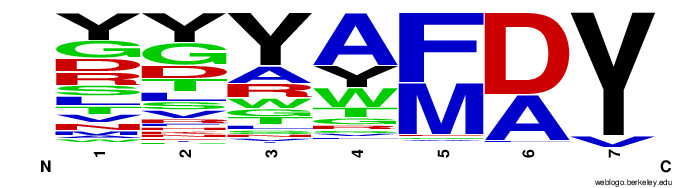


**B-1b**


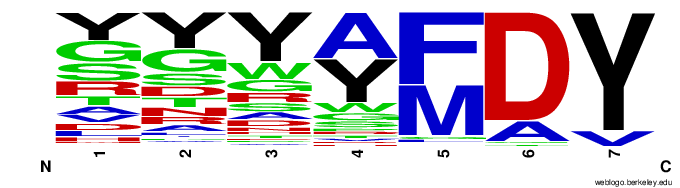

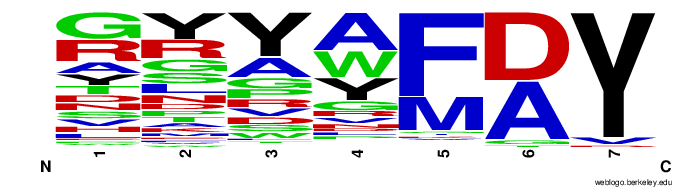


(Picture size 60% height, 45% width of original)

### WT sequences, Amino and Carboxy Termini.

**All WT Sequences**

**Frequency Plot**

**Amino Terminus Carboxy Terminus**

**Fxn E**


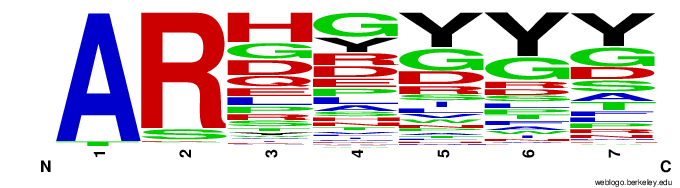

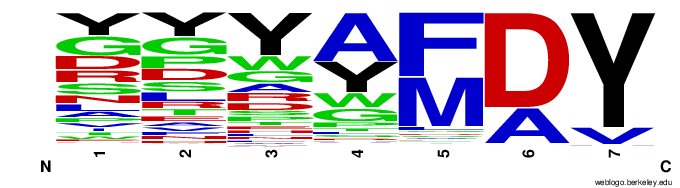


**B-2**


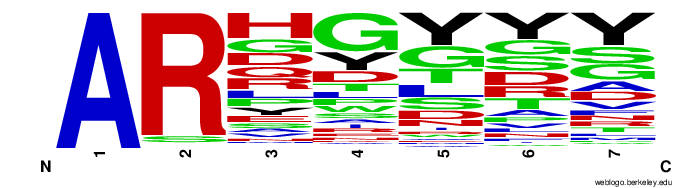

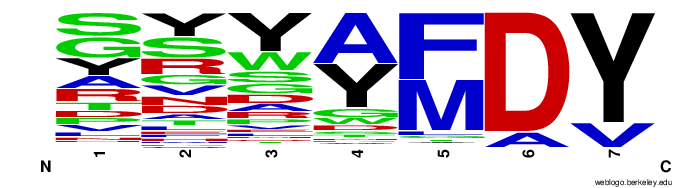


**B-1a**


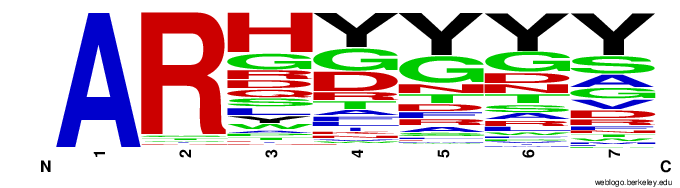

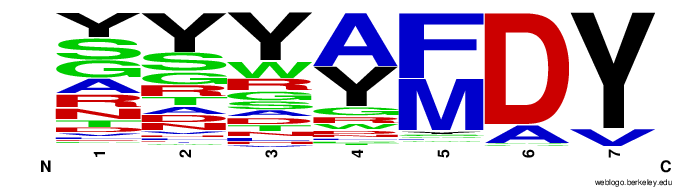


**B-1b**


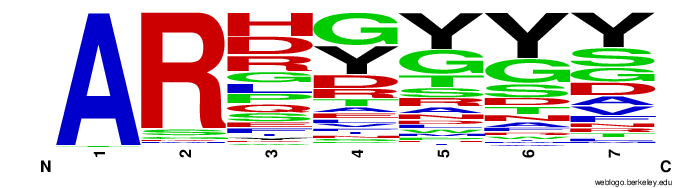

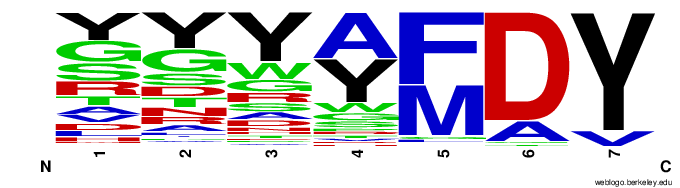


(Picture size 60% height, 45% width of original)

### λ5ko sequences, Amino and Carboxy Termini.

**All** **λ5ko Sequences**

**Frequency Plot**

**Amino Terminus Carboxy Terminus**

**Fxn E**


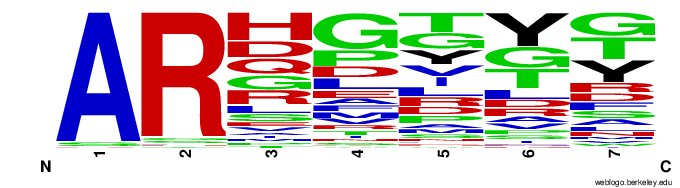

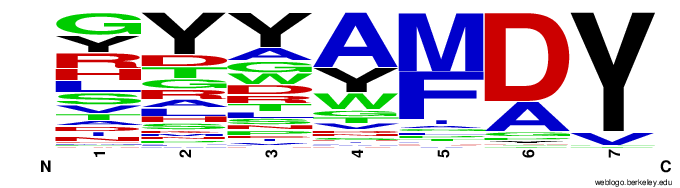


**B-2**


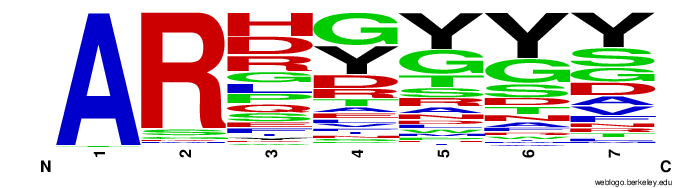

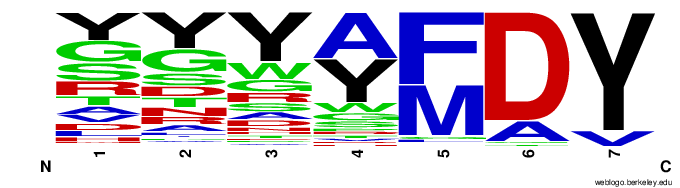


**B-1a**


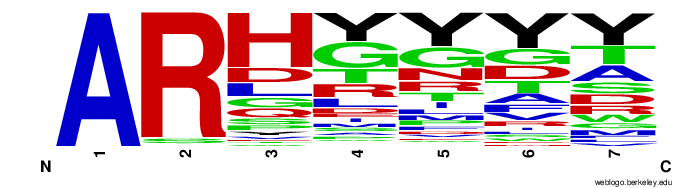

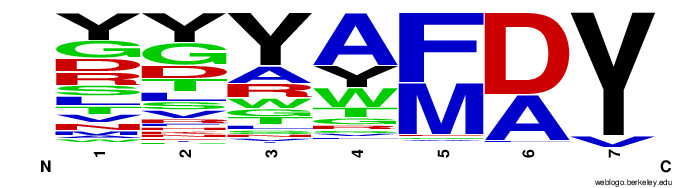


**B-1b**


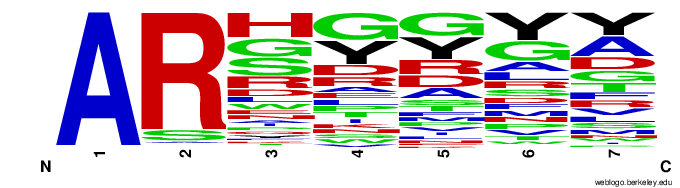

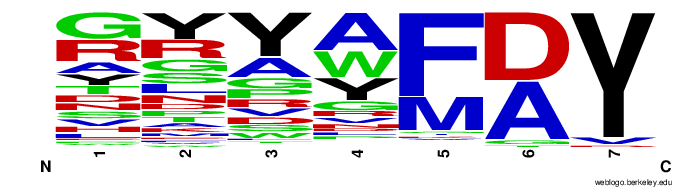


(Picture size 60% height, 45% width of original)

## DFL-Containing Sequences

### Counting from the amino terminus end.

**DFL-Containing Sequences**

***Amino Terminus of CDR-H3***

**Wild type λ5ko**

**Fxn E**


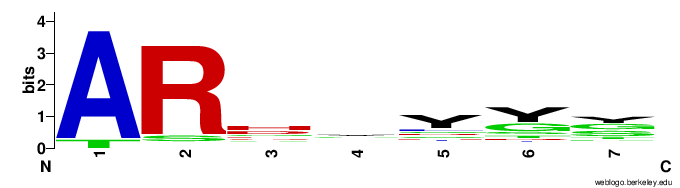

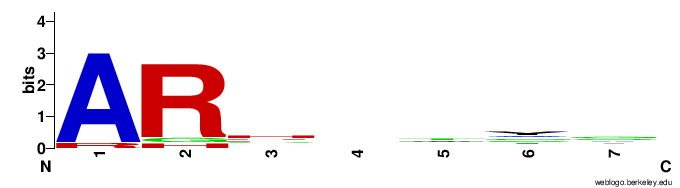


**B-2**


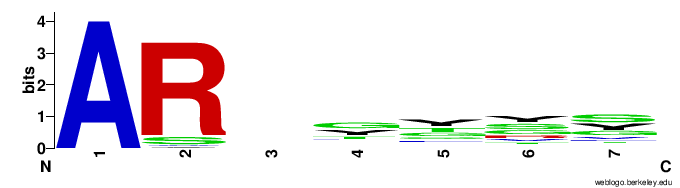

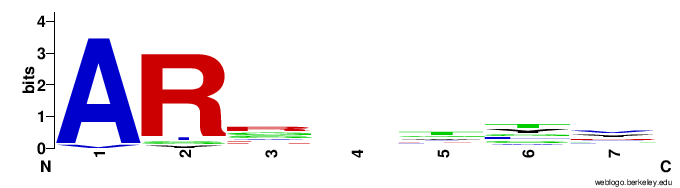


**B-1a**


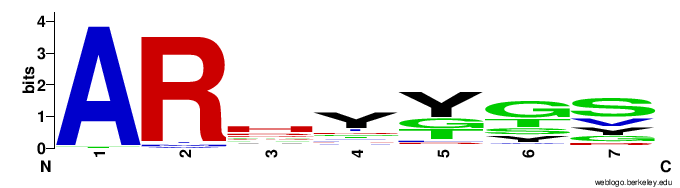

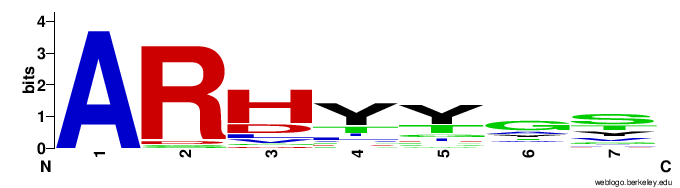


**B-1b**


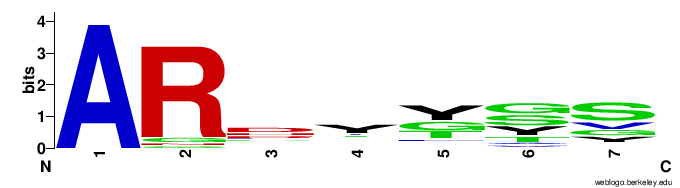

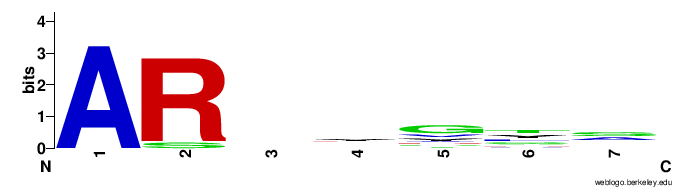


(Picture size 60% height, 45% width of original)

**DFL-Containing Sequences**

**Frequency Plot**

***Amino Terminus of CDR-H3***

**Wild type λ5ko**

**Fxn E**


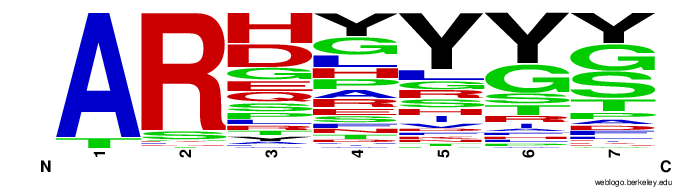

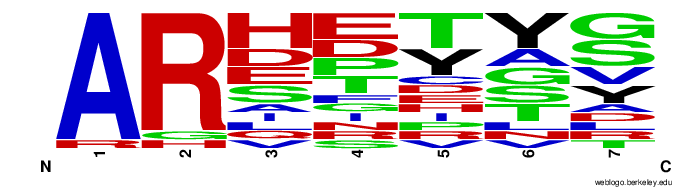


**B-2**


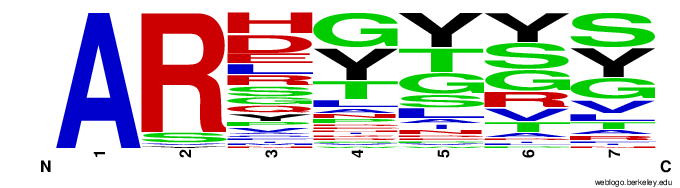

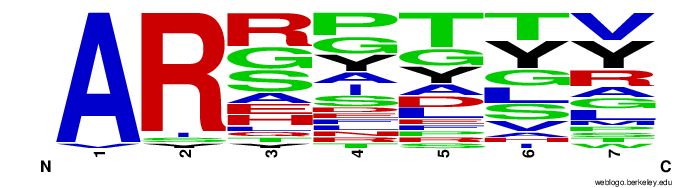


**B-1a**


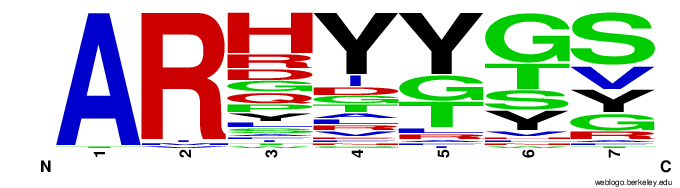

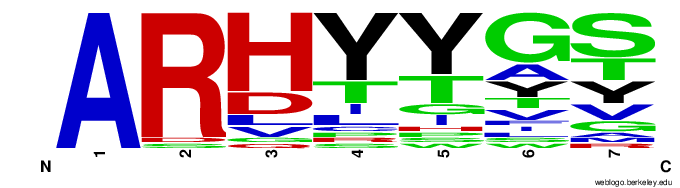


**B-1b**


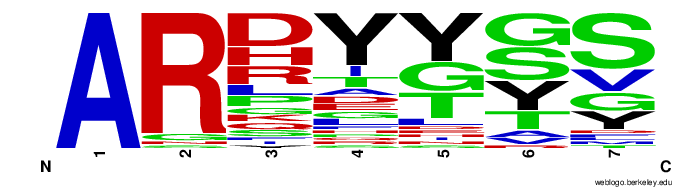

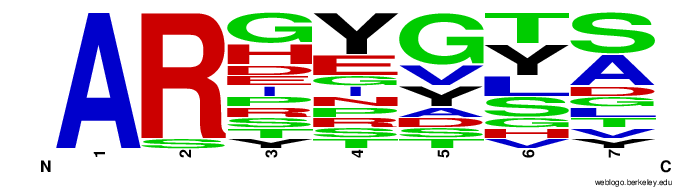


(Picture size 60% height, 45% width of original)

### Counting from the Carboxy terminus end.

**DFL-Containing Sequences**

***Carboxy Terminus of CDR-H3***

**Wild type λ5ko**

**Fxn E**


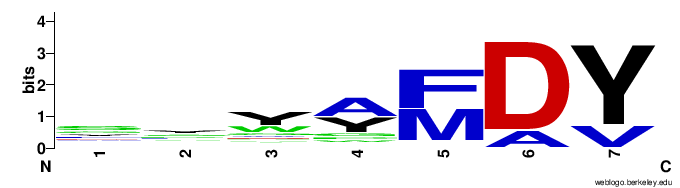

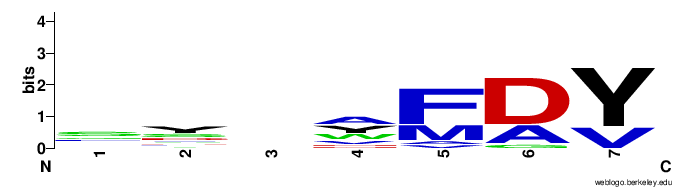


**B-2**


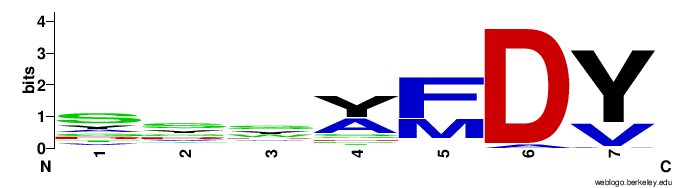

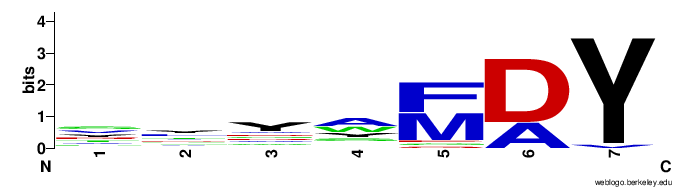


**B-1a**


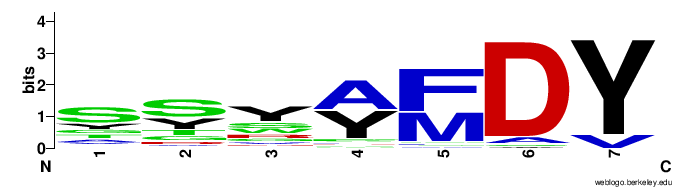

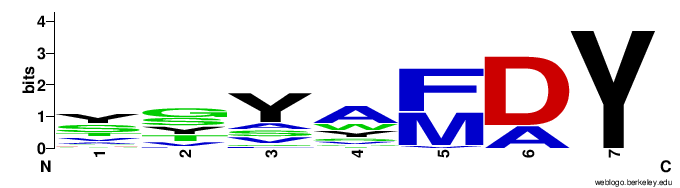


**B-1b**


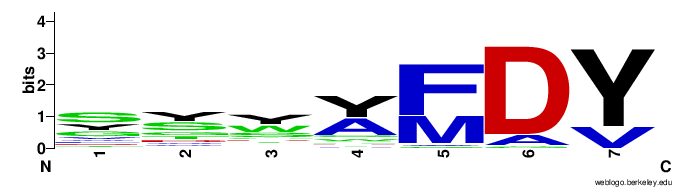

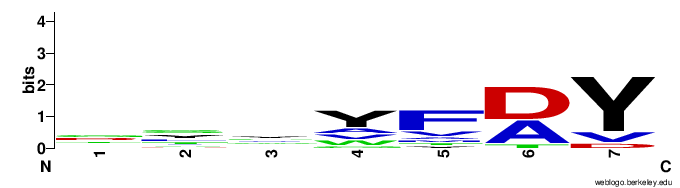


(Picture size 60% height, 45% width of original)

**DFL-Containing Sequences**

**Frequency Plot**

***Carboxy Terminus of CDR-H3***

**Wild type λ5ko**

**Fxn E**


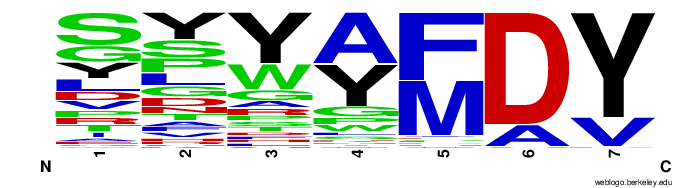

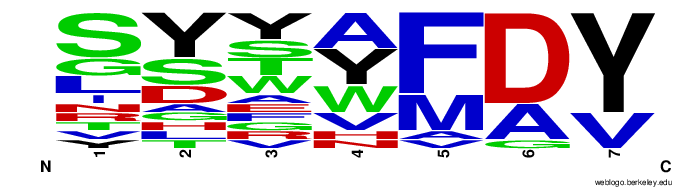


**B-2**


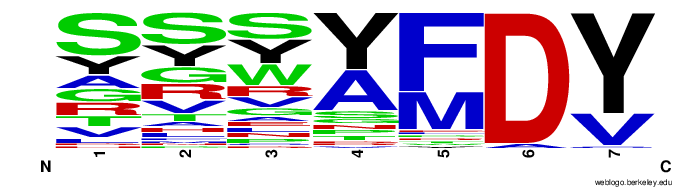

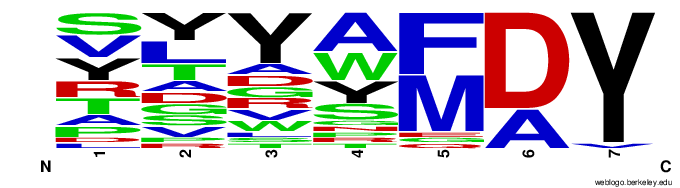


**B-1a**


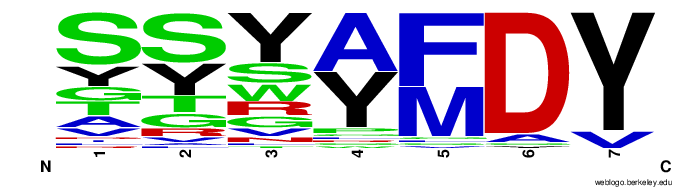

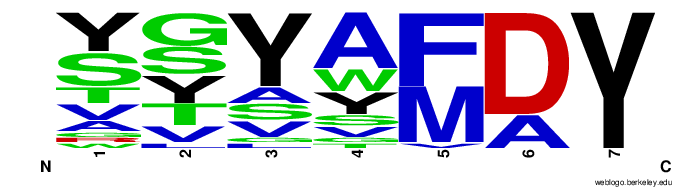


**B-1b**


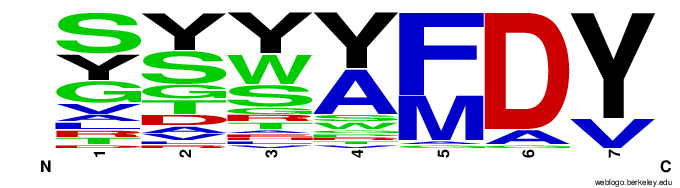

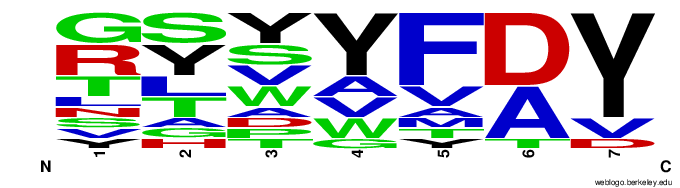
R

(Picture size 60% height, 45% width of original)

### WT DFL-Containing, Amino and Carboxy Termini.

**WT** **DFL-Containing Sequences**

**Frequency Plot**

**Amino Terminus Carboxy Terminus**

**Fxn E**


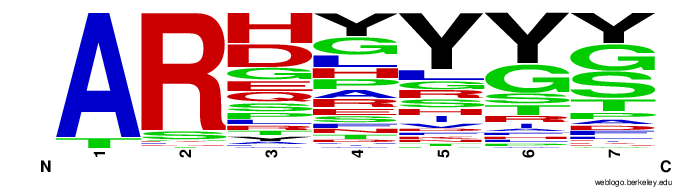

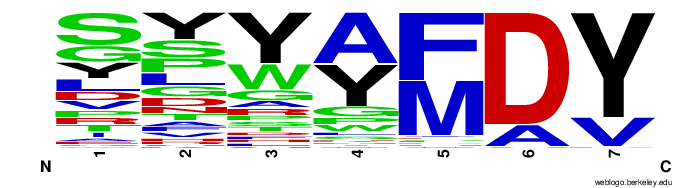


**B-2**


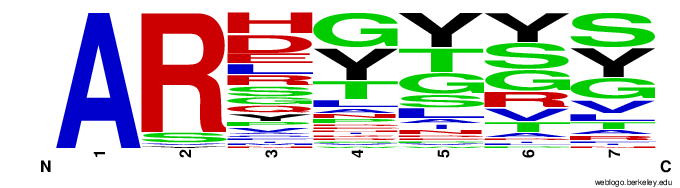

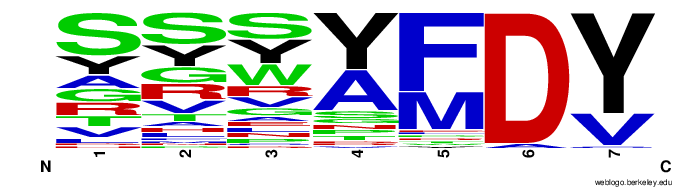


**B-1a**


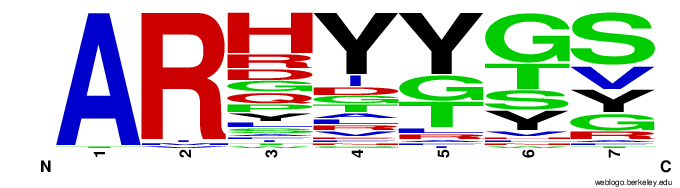

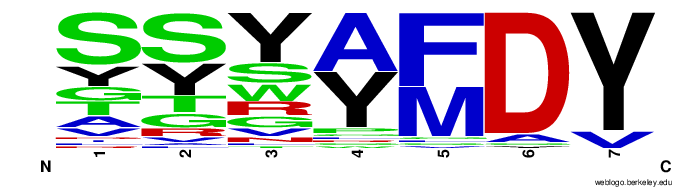


**B-1b**


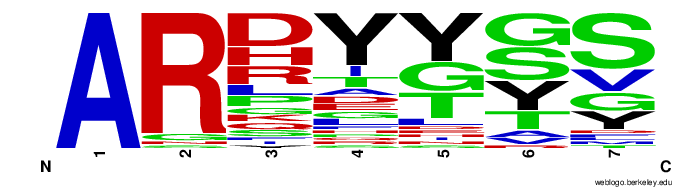

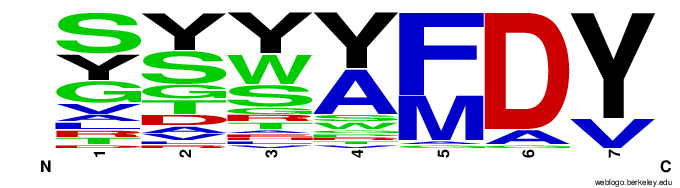


(Picture size 60% height, 45% width of original)

### λ5ko DFL-Containing, Amino and Carboxy Termini.

**λ5ko DFL-Containing Sequences**

**Frequency Plot**

**Amino Terminus Carboxy Terminus**

**Fxn E**


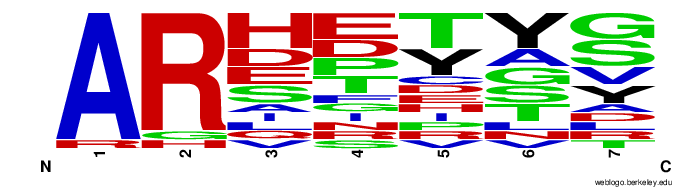

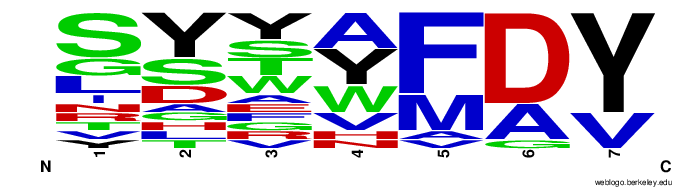


**B-2**


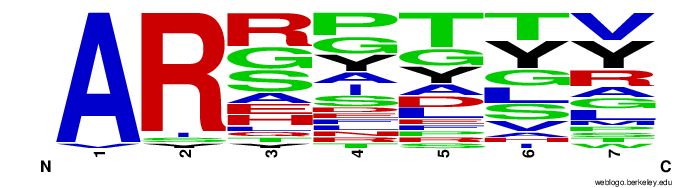

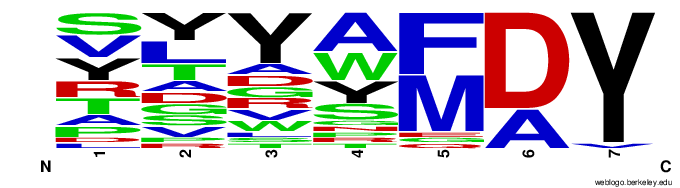


**B-1a**


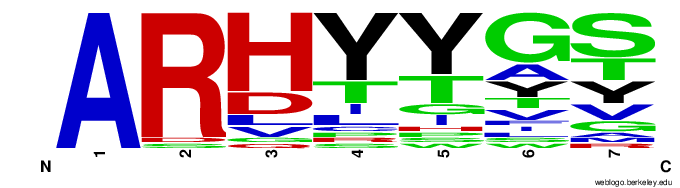

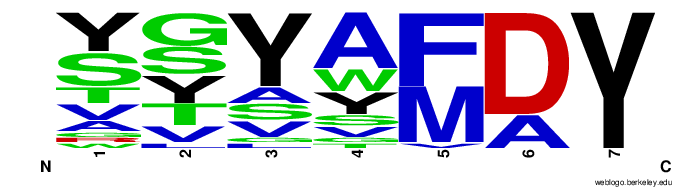


**B-1b**


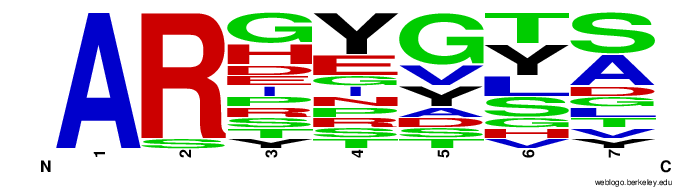

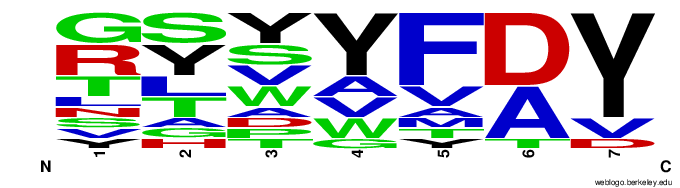


(Picture size 60% height, 45% width of original)

## DSP-Containing Sequences

### Counting from the amino terminus end.

**DSP-Containing Sequences**

***Amino Terminus of CDR-H3***

**Wild type λ5ko**

**Fxn E**


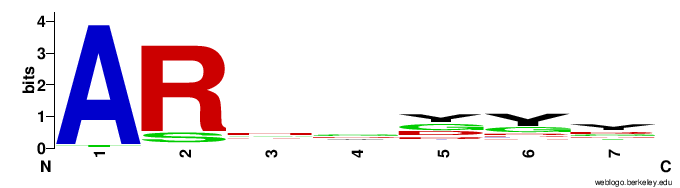

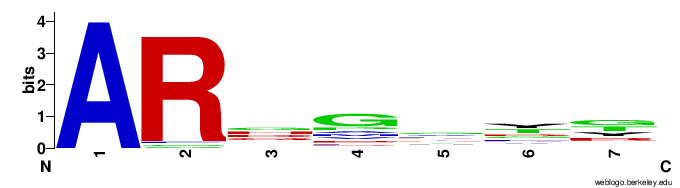


**B-2**


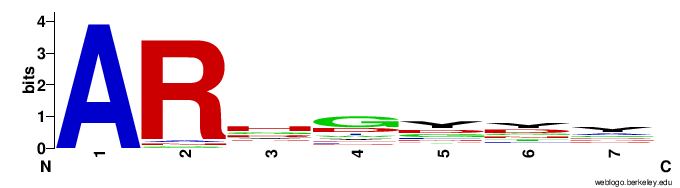


**B-1a**

**B-1b**

(Picture size 60% height, 45% width of original)

**DSP-Containing Sequences**

**Frequency Plot**

***Amino Terminus of CDR-H3***

**Wild type λ5ko**

**Fxn E**

**B-2**

**B-1a**

**B-1b**

(Picture size 60% height, 45% width of original)

### Counting from the Carboxy terminus end.

**DSP-Containing Sequences**

***Carboxy Terminus of CDR-H3***

**Wild type λ5ko**

**Fxn E**

**B-2**

**B-1a**

**B-1b**

(Picture size 60% height, 45% width of original)

**DSP Sequences**

**Frequency Plot**

***Carboxy Terminus of CDR-H3***

**Wild type λ5ko**

**Fxn E**

**B-2**

**B-1a**

**B-1b**

(Picture size 60% height, 45% width of original)

### WT DSP-Containing, Amino and Carboxy Termini.

**WT** **DSP-Containing Sequences**

**Frequency Plot**

**Amino Terminus Carboxy Terminus**

**Fxn E**

**B-2**

**B-1a**

**B-1b**

(Picture size 60% height, 45% width of original)

### λ5ko DSP-Containing, Amino and Carboxy Termini.

**λ5ko DSP-Containing Sequences**

**Frequency Plot**

**Amino Terminus Carboxy Terminus**

**Fxn E**

**B-2**

**B-1a**

**B-1b**

R

(Picture size 60% height, 45% width of original)
